# Merging Grubbs second-generation catalyst with photocatalysis enables *Z*-selective metathesis of olefins: scope, limitations, and mechanism†‡

**DOI:** 10.1039/d2sc03961c

**Published:** 2022-09-22

**Authors:** Saïf Eddine Chérif, Avisek Ghosh, Saloua Chelli, Isabelle M. Dixon, Jamil Kraiem, Sami Lakhdar

**Affiliations:** CNRS, Université Paul Sabatier, Laboratoire Hétérochimie Fondamentale et Appliquée (LHFA, UMR5069) 118 Route de Narbonne 31062 Cedex 09 Toulouse France sami.lakhdar@univ-tlse3.fr; Laboratoire de Développement Chimique, Galénique et Pharmacologique des Médicaments, Faculté de Pharmacie de Monastir, Université de Monastir Rue Avicenne 5000 Monastir Tunisia; Université de Toulouse, CNRS, Université Paul Sabatier, Laboratoire de Chimie et Physique Quantiques 118 route de Narbonne 31062 Toulouse France isabelle.dixon@irsamc.ups-tlse.fr

## Abstract

Olefin cross-metathesis is a cornerstone reaction in organic synthesis where stereoselectivity is typically governed by the structure of the catalyst. In this work, we show that merging Grubbs second generation catalyst, a classical *E*-selective catalyst, with a readily available photocatalyst, enables the exclusive formation of the contra-thermodynamic *Z*-isomer. The scope and limitations of this unprecedented approach are discussed based on both computational and experimental mechanistic data.

## Abstract 2

*Light is magic!* The combination of Grubbs second generation catalyst, a well-known catalyst for *E*-selective olefin metathesis, with a photosensitizer enables efficient access to the contra-thermodynamic *Z*-isomers.

## Graphical abstract

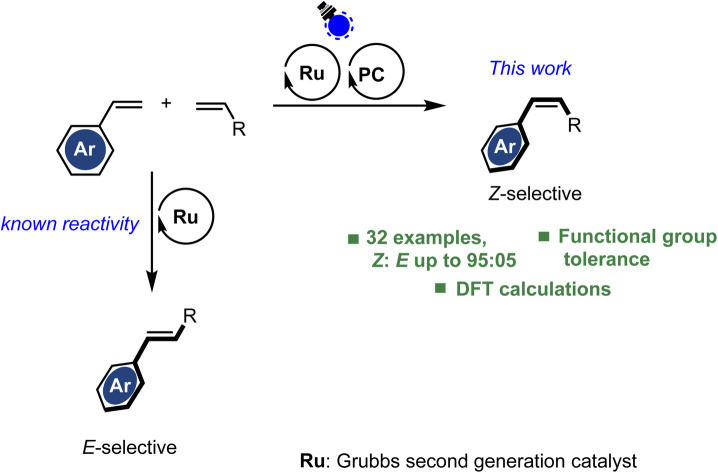

## Introduction

Olefin cross-metathesis is undoubtedly one of the most powerful methodologies for the formation of carbon–carbon double bonds.^1^ Thanks to the rational development of robust metathesis catalysts, this chemical transformation is now involved in several branches of science, including synthetic organic chemistry, material science, and biochemistry.^2^

**Scheme 1:**
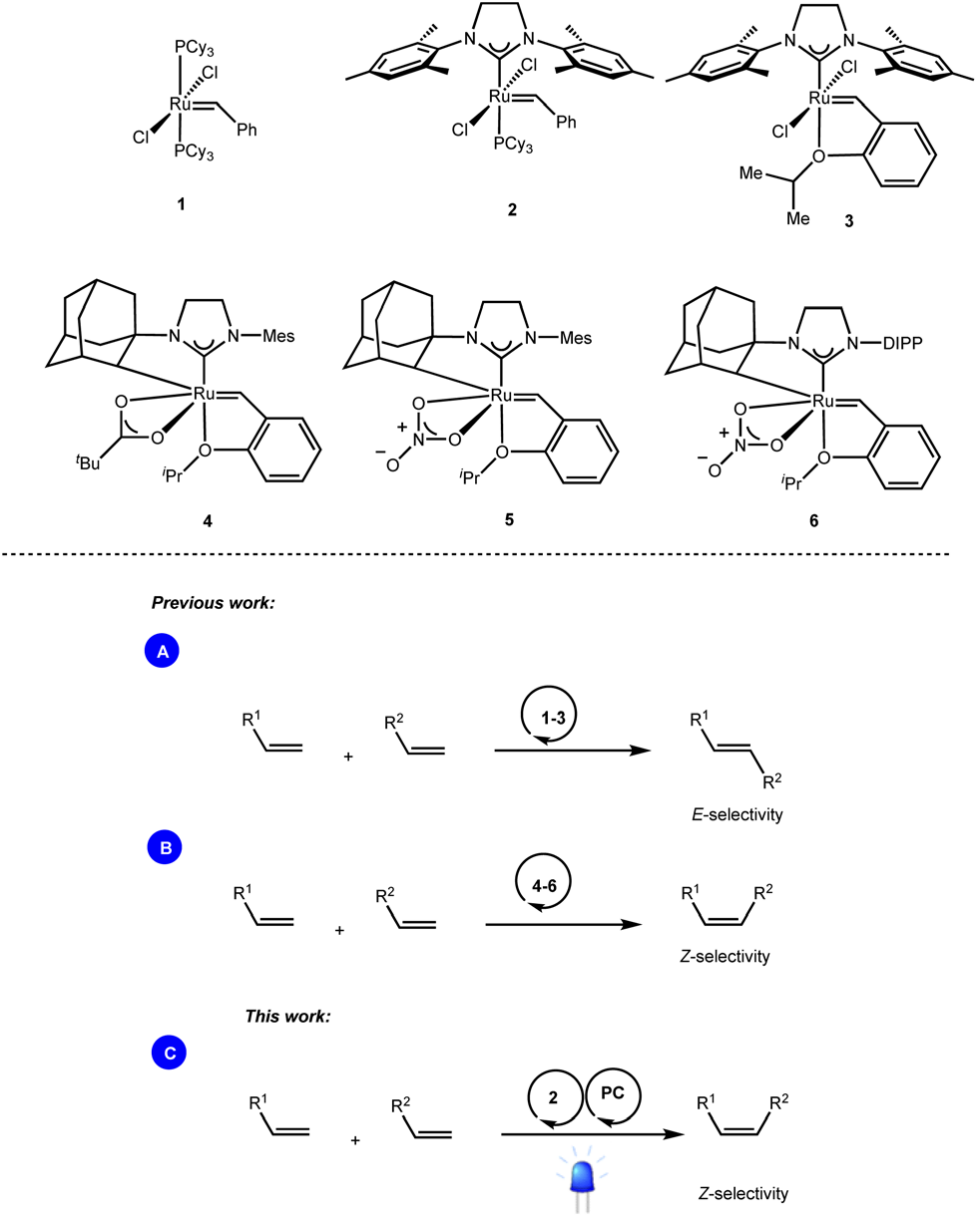
Ru-based catalysts enabling stereoselective olefin cross-metathesis reactions.

While bench-stable and easily accessible metathesis catalysts such as the well-known Grubbs (1, 2) and Grubbs–Hoveyda (3) catalysts enabled the exclusive formation of the thermodynamically favored *E*-olefins (Scheme 1A),^3^ access to the contra-thermodynamic *Z*-isomers required the use of sophisticated transition metal catalysts. For instance, while Hoveyda and Schrock developed robust monoaryloxide pyrrolide complexes of molybdenum,^4^ and tungsten,^5^ Grubbs and others disclosed ruthenium complexes containing cyclometalated NHC architectures (4–6) as powerful *Z*-selective cross metathesis catalysts (Scheme 1B).^6^

Based on the ability of visible light photocatalysis to promote *E* → *Z* isomerization of olefins,^7^ as demonstrated by the seminal works of Weaver,^8^ Gilmour^9^ and many others,^10^ we reasoned that the combination of this approach with standard ruthenium-based metathesis catalysts would provide practically simple one-pot access to *Z*-olefins. However, the feasibility of this dual catalytic (Ru/PC) system depends on the compatibility of the metathesis catalysts as well as the different intermediates generated during the metathesis process with the photocatalytic conditions (Scheme 1C).^11^

We report herein a different approach where the *E* : *Z* outcome of the metathesis reaction is not relevant anymore as we have coupled it with energy transfer that effectively drives the reaction towards the contra-thermodynamic *Z* isomer.

## Results and discussion

To test our hypothesis, we first selected homodimerization of styrene 7a in the presence of the Grubbs second-generation catalyst 2 with different photocatalysts. The choice of the catalyst 2 was motivated by a recent study by Erasmus *et al.*, who investigated the electrochemistry of this catalyst and showed that it follows an EC mechanism, where the first oxidation step is fast and reversible followed by a slow chemical step.^12^ This implies that 2 should remain stable in the presence of a photocatalyst if the back-electron event is fast.

As the metathesis reaction strongly depends on various parameters (solvent, stoichiometry, temperature…), we used conditions previously reported by Grubbs *et al.*, where the highest yields are attained when 2 is used as a catalyst and CH_2_Cl_2_ as the solvent.^13^ We thus focused on the optimization of the photocatalyst under 420 nm irradiation.

As shown in Table 1, different photocatalysts were tested in the homodimerization of styrene 7a, and both organometallic (PCa, entry 1) and organic (PCb–PCh, entries 2–8) dyes yielded the desired stilbene 8a in good to excellent yields (46 to 99%), thus demonstrating that neither photocatalyst nor blue light irradiation inhibited the metathesis process. Importantly, PCa and PCf–PCh possessing higher triplet energies gave decent *Z* : *E* ratios (80 : 20 to 90 : 10).^7^ These results are in good accordance with Zhang's report that cyanoarenes are competent catalysts for stilbene photoisomerization.^14^ The low *Z* : *E* ratio obtained when riboflavin (PCb, entry 2) is used as a photocatalyst is obviously due to its low solubility in dichloromethane.

**Table 1:** Screening of photocatalysts activity and *Z* : *E* selectivity

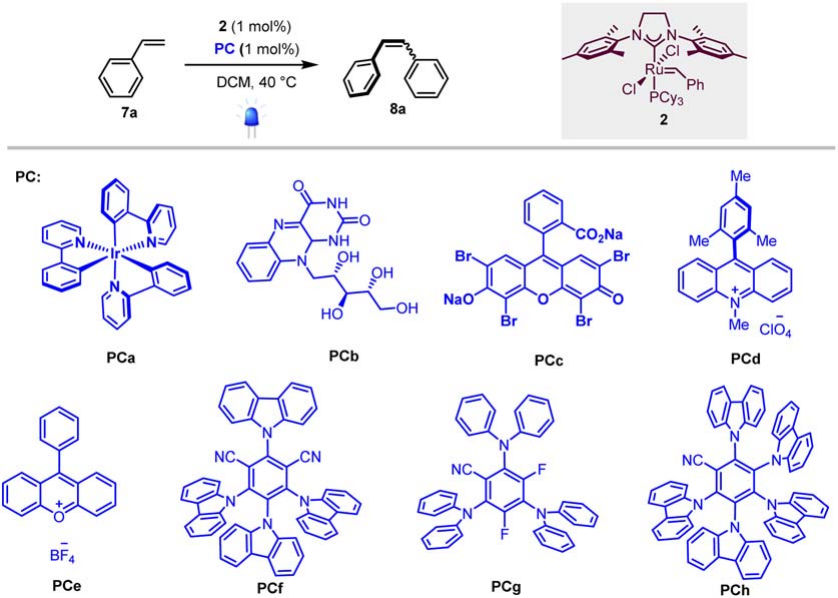			
Entry	Photocatalyst	Yield^a^ [%]	*Z* : *E* (ratio)
1	PCa	92	83 : 17
2	PCb	46	10 : 90
3	PCc	91	5 : 95
4	PCd	88	58 : 42
5	PCe	99	0 : 100
**6**	PCf	**99**	**90 : 10**
7	PCg	94	80 : 20
8	PCh	95	83 : 17
9	—	99	5 : 95
10	PCf^b^	—	—
11	PCf^c^	99	0 : 100
12	PCf^d^	64	87 : 13

a Combined yield of *E* and *Z* isomers of the stilbene determined by ^1^H NMR spectroscopy.

b In the absence of metathesis catalyst 2.

c In the dark.

d At room temperature.

Based on these results, photocatalyst PCf was selected for further optimization evaluations. Unsurprisingly, while the *E* isomer was obtained exclusively (*E* : *Z* = 95 : 5, entry 9) in the absence of photocatalyst, the reaction didn't proceed without Grubbs second-generation catalyst (2, entry 10). Finally, isomerization didn't proceed in the dark (entry 11), and yield and stereoselectivity dropped when the reaction was carried out at room temperature (entry 12).

With the optimized conditions in hand, we next evaluated the scope of the reaction by testing different styrenes bearing electron-donating or accepting groups at the para or the meta positions of the aromatic ring (Table 2). Homodimerization tolerates some functionalities (chloro, fluoro, and ester) and is scalable. Interestingly, *Z*-stilbenes were obtained in good to excellent conversions (66 to 99) and high selectivity (*Z* : *E* ranging from 87 : 13 (8e and 8f) to 91 : 9 (8a)) except in the case of naphthyl stilbene 8i (*Z* : *E* = 75 : 25).

**Table 2:** Scope of the metathesis reaction

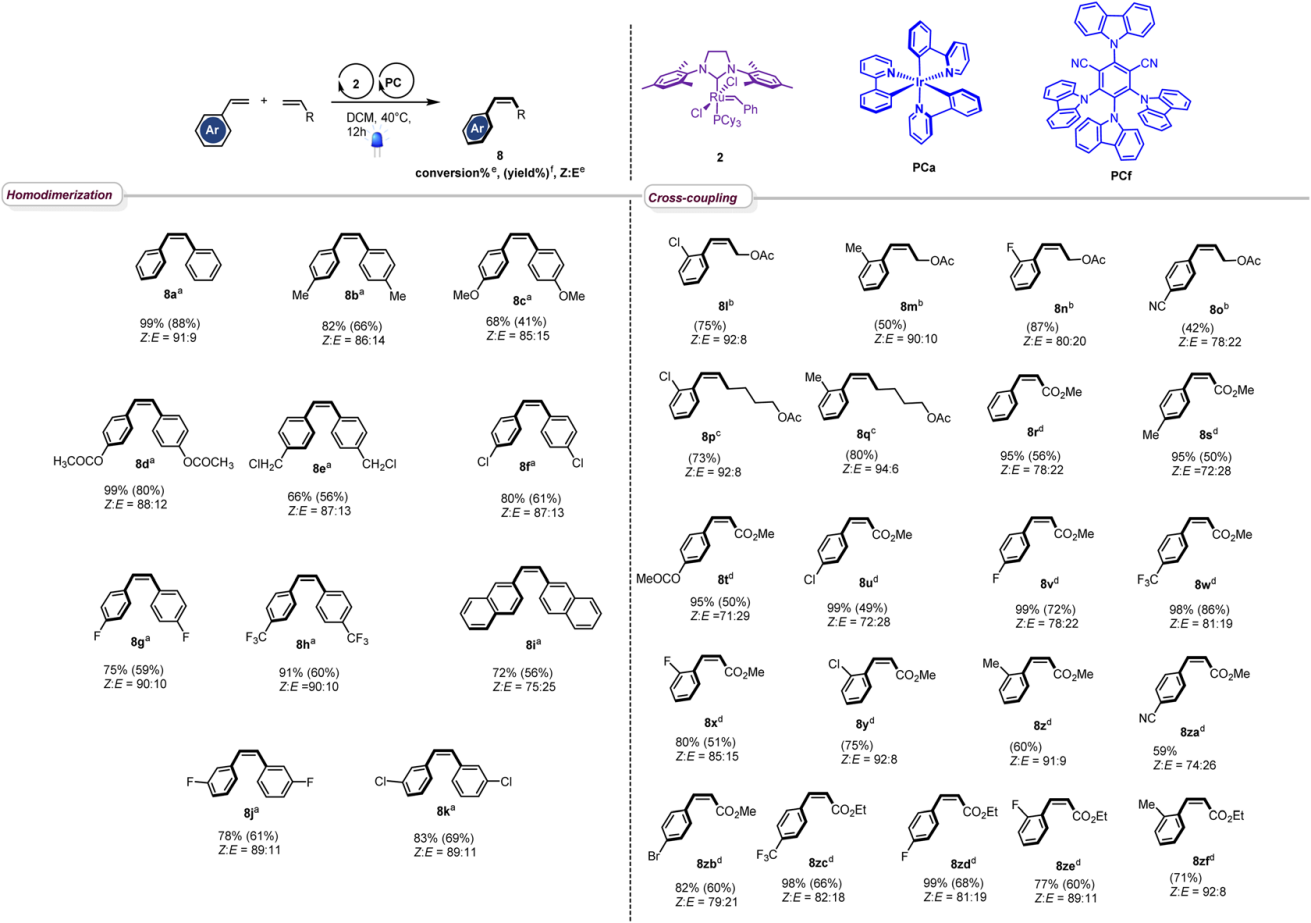

a Reaction conditions for homodimerization: styrene (1.0 eq, 0.10 M) in DCM, catalyst 2 (1 mol%) and catalyst PCf (1 mol%), 40 °C, 12 h, N_2_, 420 nm.

b Reaction conditions for cross-coupling reaction of styrene derivatives with *cis*-2-butene-1,4-diacetate: styrene (1.0 eq, 0.20 M) in DCM, *cis*-2-butene-1,4-diacetate (2.4 eq), catalyst 2 (5 mol%), catalyst PCa (5 mol%), 40 °C, 24 h, N_2_, 420 nm.

c Reaction conditions for cross-coupling of styrene derivates with 5-hexenyl-1-acetate: styrene (3.0 eq), 5-hexenyl-1-acetate (1.0 eq, 0.18 M) in DCM, catalyst PCa (5 mol%), catalyst 2 (5 mol%), 40 °C, 24 h, N_2_, 420 nm.

d Reaction conditions for cross-coupling of styrene derivates with acrylate: styrene derivatives (1.0 eq, 0.05 M) in DCM, alkyl acrylate (2.0 to 5.0 eq), catalyst 2 (2 mol%), catalyst PCf (5 mol%), 40 °C, 24 h, N_2_, 420 nm.

e Conversion and *Z* : *E* ratio are calculated from NMR spectra of the crude reaction mixture.

f Isolated yields.

Interestingly, in accordance with Grubbs' general empirical model for olefin reactivity,^13^ the reaction is amenable to olefin cross-metathesis with aliphatic olefins 8l–8q. In these cases, improved yields and selectivities were observed when PCa was used instead of PCf. As depicted in Table 2, good yields were attained when aliphatic olefins are combined with styrenes bearing a substituent at the *ortho* position of the aromatic ring. Interestingly the reaction works smoothly with 4-cyanostyrene, leading to the desired adduct 8o in good yield and *Z* : *E* = 74 : 26. Moreover, the reaction proceeds well with acrylates and good to excellent conversions were obtained (8r–8zf). The stereoselectivity observed in these cases was fair to excellent and the process shows good compatibility with various functionalities (ester (8t), chloro (8u), fluoro (8v), trifluoromethyl (8w), cyano (8za), and bromo (8zb)), confirming the broad scope of this novel tandem experimental protocol.

In order to gain further insights into the reaction mechanism, experimental and computational experiments were conducted.

**Fig. 1:**
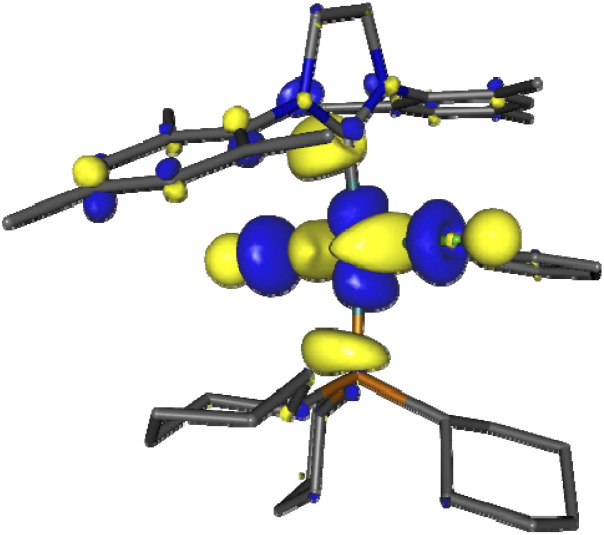
Kohn–Sham LUMO+7 of Grubbs catalyst (2), showing the antibonding Ru–P interaction involved in PCy_3_ photorelease.

We first examined the interaction of blue light with Grubbs second generation catalyst to confirm that light was not deleterious to the metathesis process. In this context, several DFT calculations were performed, starting with inspecting the optimized ground state molecular orbitals (Fig. S8, page S27‡) and analyzing the TD-DFT calculation of Franck–Condon excited states (Table S5, page S28‡). For catalyst 2, the population of LUMO+7, which is the dσ* antibonding Ru–P MO (Fig. 1), would be required to photorelease PCy_3_. Excitations towards LUMO+7 are indeed the major components of singlet states S5 (3.25 eV/381 nm) and S6 (3.41 eV/363 nm) at the ground state geometry. Their oscillator strength is small with respect to that of state S7 (3.56 eV/348 nm) but not neglectable, thus dissociative ^1^MC states can be directly populated upon 355 nm irradiation, or more likely upon excitation to S7 followed by internal conversion to S6 or S5. The corresponding dissociative ^3^MC states (T6 at 2.69 eV and T7 at 2.93 eV) are expected to be similarly populated through additional intersystem crossing facilitated by spin–orbit coupling due to the metal center. These computational results are fully consistent with previous experimental results showing the efficiency of near visible light irradiation to generate 14-electron complexes from 2 or related compounds.^12,15^

**Fig. 2:**
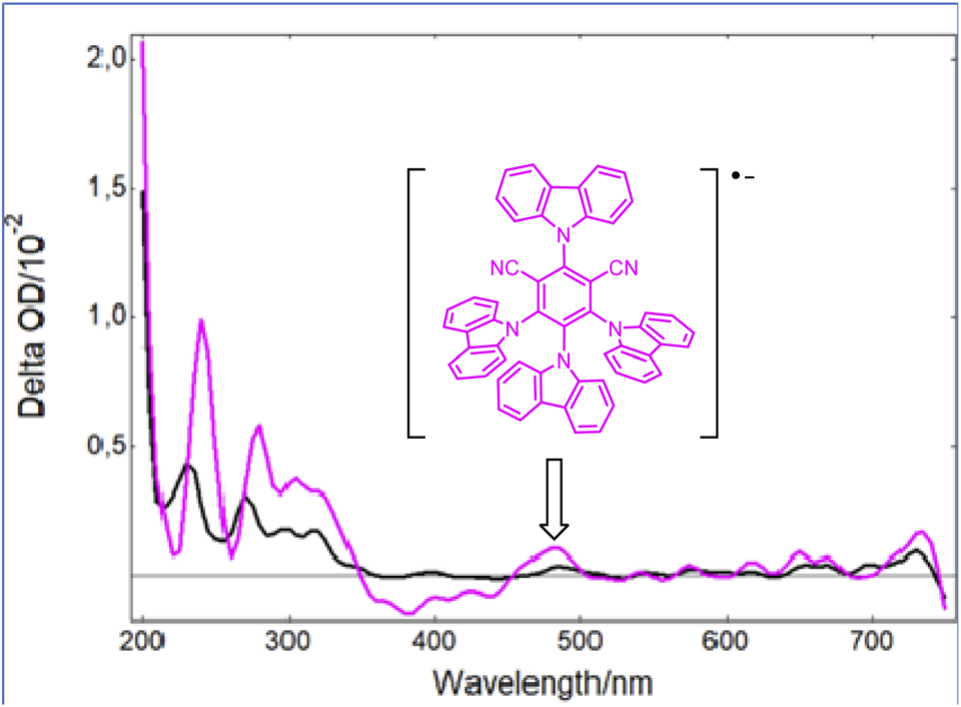
Transient UV-vis spectra following irradiation of Grubbs second-generation catalyst (2) at 355 nm (black) and a mixture of 2 with PCf (pink) at the same wavelength.

However, as the molar extinction coefficient of Grubbs catalyst 2 is much lower than that of the photocatalyst PCf at 420 nm, it is then likely that only the latter would be active upon blue light irradiation. Indeed, as shown in Fig. 2, while a strong bleaching of the ground state of PCf was observed after a laser excitation at 355 nm, a new absorption band at 465 nm appeared when 2 was introduced into a solution of PCf. Based on a previous report by Wu,^16^ this new peak is assigned to the radical anion (PCf)˙^−^. This efficient single electron transfer matches the redox potentials PCf* (*E*° = 1.41 V/SCE) and 2 (*E*° = 0.25 V/SCE) of both components. Additionally, the Stern–Volmer luminescence quenching experiment evidenced linear correlation with respect to the metathesis catalyst 2. This shows that 2 is an effective reductive quencher of PCf* (*K*_sv_ = 2.34 × 10^4^ M^−1^) (Fig. S3, page S22‡). It should be noted that quenching studies with styrene were less effective (Fig. S4, page S23‡).

**Scheme 2:**
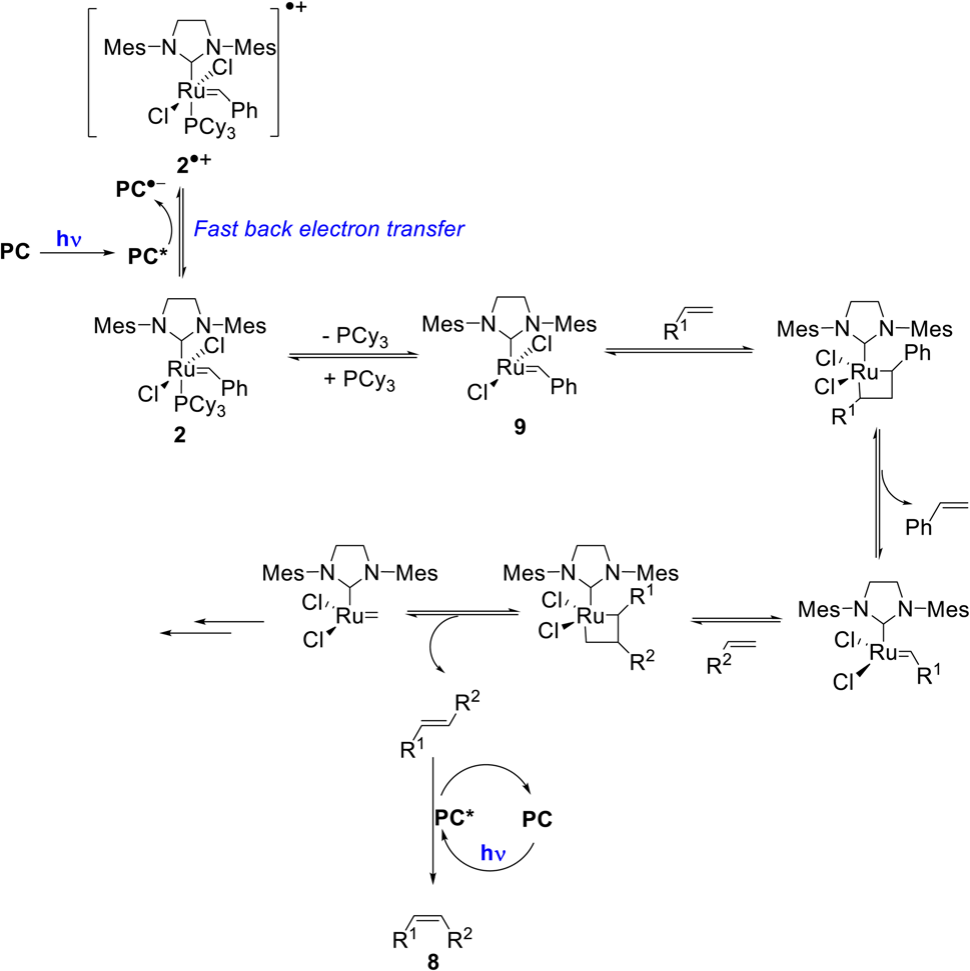
Proposed reaction mechanism for the tandem olefin cross metathesis and *E*–*Z* isomerization (some key intermediates in the metathesis mechanism are omitted for the sake of clarity).

Importantly, once catalyst 2 is oxidized, fast back-electron transfer (BET) was observed by transient spectroscopy and measured to be diffusion-limited (*k*_BET_ = 4 × 10^10^ M^−1^ s^−1^).^17^ This efficient process results in the generation of the 14-electron complex 9 through a classical dissociative pathway from neutral 2 rather from its radical cation (2)˙^+^ as in the case of bis-NHC ligated Ru complexes.^18^ Oxidation of 2 by *PC is thus not problematic since BET is fast (Scheme 2).

Having these results in mind, we next investigated computationally the structure of the 14-electron complex and its interaction with light. The ground state geometry of 9 reflects the freedom for Ph(ben) to occupy the volume set free by releasing the bulky PCy_3_, and moderate pyramidalization at the ruthenium centre (ben = benzylidene). Marked shortenings of the Ru–C(car) (car = *N*-heterocyclic carbene) and Ru–Cl distances are also noteworthy between 2 and 9 (Table S7, page 33‡).

The most remarkable ground state Kohn–Sham orbitals of 9 are the dσ* antibonding LUMO+1 (towards car) and LUMO+2 (towards ben), both displaying small contributions towards the bound C atom and large contributions in the vacant space opposite (Fig. S9, page S29‡). Population of LUMO+1 or LUMO+2 is therefore expected to lead to slight Ru–C elongations, but not to the extent of inducing ligand release. No photoinstability is thus envisioned for 9, particularly in the event of 355 nm light absorption to its most absorbing states S7 and S8 (Table S5, page S28‡).

The lowest triplet excited states of 2 and 9 were then optimized in the unrestricted DFT formalism. Both are of Ru-ben MLCT character, as illustrated on their spin density plots (Fig. S3‡). The Gibbs free energy difference between *2-^3^MLCT and 2 is low (0.90 eV/20.7 kcal mol^−1^), and even lower between *9-^3^MLCT and 9 (0.63 eV/14.6 kcal mol^−1^) (Table S8, page S34‡). This implies that triplet–triplet energy transfer from such low-lying triplets to the organic species present, and particularly the metathesis product *E*-stilbene, is thermodynamically not feasible. These results show clearly that the metathesis catalyst 2 and the 14-electron complex 9 are not responsible for the observed alkene photoisomerization.

In the case of complex 9, a ^3^MC state displaying an elongated Ru–C(car) distance at 2.096 Å (Table S7, Fig. S11‡) could be optimized and is located 20.0 kcal mol^−1^ above 9 (Gibbs free energy difference). This triplet state is nearly degenerate with its associated minimum energy crossing point (the lowest energy point of singlet–triplet degenerescence, relevant to intersystem crossing), therefore very efficient nonradiative deactivation processes can be expected in the 14-e complex. The existence of this low-lying ^3^MC state is not relevant to metathesis itself but could be involved in catalyst photodegradation mechanisms.

To sum up, the minor fraction of light absorbed by complex 2 is not deleterious in that it could lead to PCy_3_ loss or nonradiative deactivation. If light is absorbed by complex 9, efficient nonradiative deactivation channels are also available.

Regarding triplet–triplet energy transfer (EnT) processes, we have already established that the triplet states of 2 and 9 are too low lying to allow energy transfer to the organic compounds present in the reaction vessel (Scheme 2). We then optimized the triplet states of the other protagonists, particularly the photosensitizer PCf and the metathesis product, *E*-stilbene, which eventually gets isomerized into its *Z* form. The energy content of the triplet state PCf* is 51.3 kcal mol^−1^, *i.e.* larger than that of *E*-stilbene (45.8 kcal mol^−1^) (Table S4‡). This demonstrates the exergonicity of EnT from PCf* to *E*-stilbene, which in turn triggers *E*–*Z* isomerization.

Based on these mechanistic data, we propose the reaction mechanism shown in Scheme 2, which starts with the photoexcitation of PC to PC*, which is reductively quenched by the metathesis catalyst 2 to generate (PC)˙^−^ and (2)˙^+^. Due to a diffusion-limited BET, phosphine loss occurs from 2 rather than from its oxidized form. Then, the classical metathesis mechanism takes place to yield the *E*-alkene. A subsequent photoisomerization of the latter by PC* leads to the formation of the desired *Z*-isomer 8. The strong point is that for simplicity, the reaction is performed one-pot under continuous irradiation.

## Conclusion

We have developed an orthogonal tandem catalytic (metathesis/photoisomerization) transformation enabling facile access to *Z*-olefins from the standard *E*-selective Grubbs second-generation catalyst. Joint experimental and computational investigations showed that the feasibility of this reaction results from a combination of (i) light being mostly absorbed by the photocatalyst PC, (ii) diffusion-controlled back electron transfer restoring Grubbs catalyst following its oxidation by PC* (iii) formation of the classical metathesis product *E*-alkene, and (iv) *E*–*Z* photoisomerization by a final energy transfer event from PC*.

Finally, it is worth mentioning that metathesis reactions can often lead to positional isomerization or thermodynamic (*E*-selective) geometric isomerization due to the formation of ruthenium hydride complexes.^19^ However, our results show that a high level of *Z*-selectivity can be achieved without any additives. This indicates that competitive ground state metal hydride isomerization processes can be avoided under our conditions.

This work opens new avenues in the exploration of photometathesis that should lead to the development of practically simple approaches for the synthesis of *Z*-olefins.

## Data availability

Experimental and computational data have been provided in the ESI.‡

## Author contributions

S. E. C. and A. G. performed the synthetic experiments; S. C. performed the laser flash photolysis and fluorescence experiments; I. M. D. performed the computational studies; and S. L. designed and supervised the project. All authors analysed the results.

## Conflicts of interest

There are no conflicts to declare.

## Supplementary Material

SC-013-D2SC03961C-s001

SC-013-D2SC03961C-s002

SC-013-D2SC03961C-s003

SC-013-D2SC03961C-s004

SC-013-D2SC03961C-s005

SC-013-D2SC03961C-s006

SC-013-D2SC03961C-s007

SC-013-D2SC03961C-s008

SC-013-D2SC03961C-s009

SC-013-D2SC03961C-s010

SC-013-D2SC03961C-s011

SC-013-D2SC03961C-s012

SC-013-D2SC03961C-s013

SC-013-D2SC03961C-s014

SC-013-D2SC03961C-s015

SC-013-D2SC03961C-s016

SC-013-D2SC03961C-s017

SC-013-D2SC03961C-s018
