# Supplementary material for: Merging Grubbs second-generation catalyst with photocatalysis enables Z-selective metathesis of olefins: scope, limitations, and mechanism
Source: Chem Sci. 2022 Sep 22;13(41):12065–70. doi: 10.1039/d2sc03961c (PMC9600307; doi:10.1039/d2sc03961c)
Supplement: SC-013-D2SC03961C-s008 [file SC-013-D2SC03961C-s008.pdf]

Electronic Supplementary Material (ESI) for Chemical Science.
This journal is © The Royal Society of Chemistry 2022

Supporting Information for:

Merging Grubbs' Second-Generation Catalyst with Photocatalysis Enables *Z*–Selective Metathesis of Olefins: Scope, Limitations, and Mechanism

Saïf Eddine Chérif,[a,b] Avisek Ghosh,[a] Saloua Chelli,[a] Isabelle M. Dixon,[*c] Jamil Kraiem,[b] and Sami Lakhdar*[a]

a. CNRS/Université Paul Sabatier, Laboratoire Hétérochimie Fondamentale et Appliquée (LHFA, UMR5069). 118 Route de Narbonne,31062 Cedex 09 Toulouse (France).

b. Laboratoire de Développement Chimique, Galénique et Pharmacologique des Médicaments, Faculté de Pharmacie de Monastir, Université de Monastir, Rue Avicenne, 5000 Monastir, Tunisia

c. Université de Toulouse, CNRS/Université Paul Sabatier, Laboratoire de Chimie et Physique Quantiques, 118 route de Narbonne, 31062 Toulouse, France.

Corresponding authors :

isabelle.dixon@irsamc.ups-tlse.fr & sami.lakhdar@univ-tlse3.fr

Table of contents

**1. General Methods and Materials**……………………………………………	**S3**
**2. Procedure for synthesis**…………………………………………………………	**S4**
**3. Mechanistic studies**………………………………………………………….	**S22**
**3.1.** UV–Visible studies……………………………………………….	**S22**
**3.2.** Fluorescence studies and Stern-Volmer analysis………………	**S22**
**3.3.** Laser flash photolysis…………………………………………	**S27**
**4. Computational details**……………………………………………………..	**S27**
**5. Copies of 1H-, 13C- and 19F-NMR Spectra**………………………..	**S37**
**6. References**………………………………………………………………………..	**S78**

1. General Methods and Materials

All reactions were carried out under argon using stand glovebox techniques (glovebox maintained at < 0.1 ppm H_2O and < 0.1 ppm O_2). Dichloromethane was dried using an MBRAUN Solvent Purification System (SPS) and sparged with argon. All chemicals were purchased from Sigma-Aldrich and used without further purifications. The catalyst 1,2,3,5-Tetrakis(carbazol-9-yl)-4,6-dicyanobenzene,2,4,5,6-Tetrakis(9H-carbazol-9-yl) isophthalonitrile (4CzIPN) was prepared according to a literature procedure.[1]

All reaction mixtures were irradiated with 5W GU10 LED Lamp Blue from a 2 cm distance. The emission maximum of the light source used is 420 nm (Figure S1).

Flash column chromatography were carried out with Merck silica gel 60 (0.040–0.063 mm). Chromatography fractions and stated reactions were monitored by TLC on Merck silica gel 60 F254 aluminum plates. The spots were visualized under UV light at 254 nm. UV-Visible spectra were recorded on a Perkin Elmer Lambda 40 Spectrophotometer. High resolution mass spectrometry (HRMS) analyses were performed on a Q Exactive Mass Spectrometer (Thermofisher) using direct injection.

^{1}H NMR (300 MHz); ^{19}F (282 MHz) and ^{13}C proton decoupling (75 MHz)· spectra were recorded on an Agilent Direct Drive spectrometer (Agilent Technologies, Santa Clara) in $CDCl_3$. The chemical shifts are given in ppm and refer to $CDCl_3$ (δ H = 7.26, δ C = 77.16) as internal standard. Coupling constants *J* are measured in Hz. Splitting patterns are designed as follows: s = singlet; d = doublet; t = triplet; q = quartet, dd = doublet of doublet; dt = doublet of triplet; m = multiplet.

Transient absorption spectra were measured with an LP920-KS spectrometer from Edinburgh Instruments.

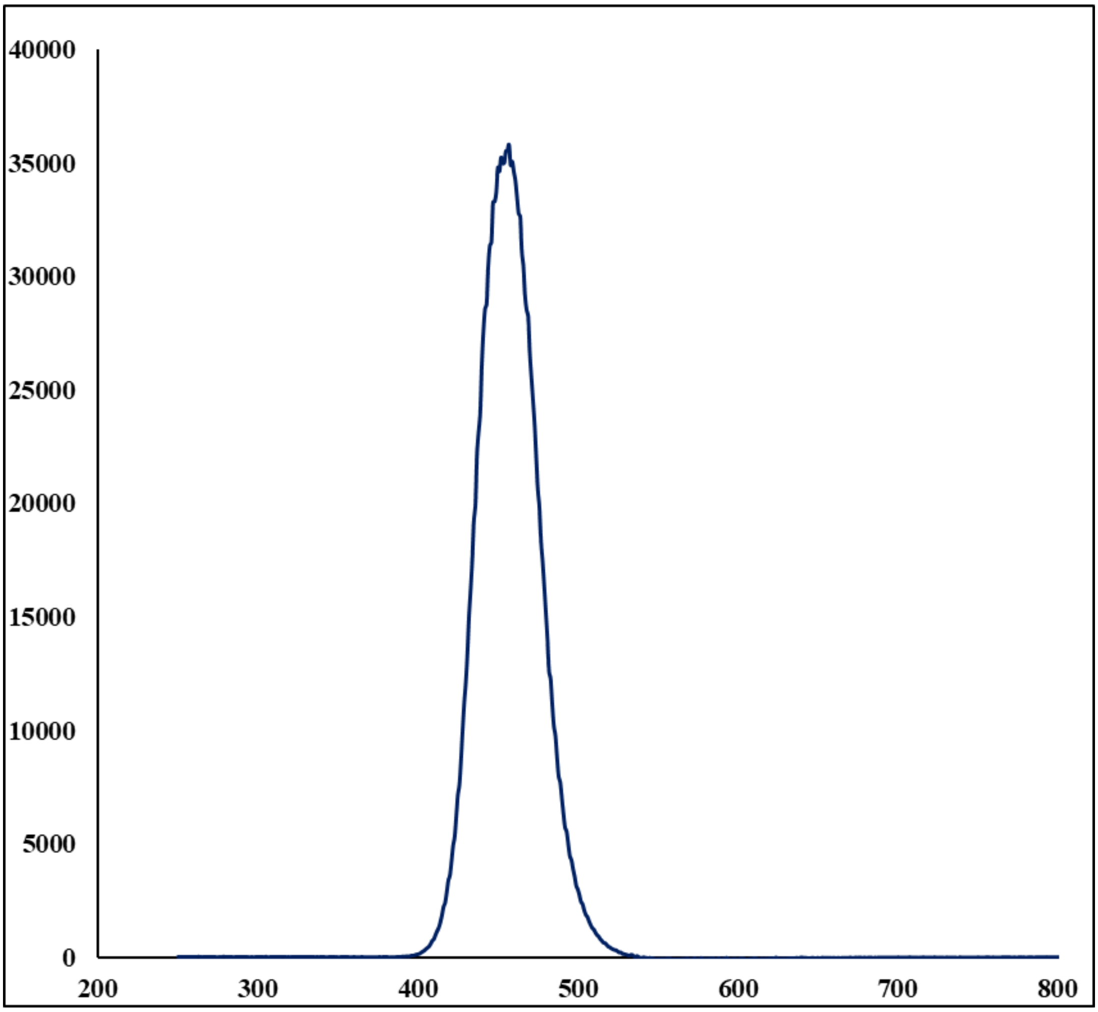

Figure S1. Emission spectrum of the blue LED lamp

2. Procedure for synthesis

General procedure for homodimerization reaction (**8a** to **8k**).[2,3]

A flame-dried quartz Schlenk tube was charged with styrene (1.0 eq, 0.1 M) and dichloromethane was added to make a final concentration of 1.0 M styrene (conc. of 0.5 M if you consider that 0.5 eq. reacts with the other 0.5 eq. to give 1.0 eq. of stilbene). Then, Grubbs 2nd generation catalyst (1 mol %) and the 4CzIPN (1 mol %) were then added in one portion. The reaction mixture was stirred at 40 °C for 12h under argon and at 420 nm. The solvent was removed under reduced pressure and the resulting residue was purified by flash chromatography on silica gel (100% pentane) to provide the *Z* isomers.

Compound (8a):

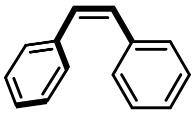Styrene (183 µL, 1.6 mmol); Grubbs 2nd generation catalyst (6.8 mg, 0.008 mmol,1 mol %); 4CzIPN (6.3 mg, 0.008 mmol,1 mol %).

Purification (colorless oil, 127 mg, 0.7 mmol, 88 %). ^{1}H NMR (300 MHz, $CDCl_3$) δ (ppm) 7.29 - 7.17 (m, 10H), 6.61 (s, 2H).^{13}C NMR (75 MHz, $CDCl_3$) δ (ppm) 137.4, 130.4, 129.0, 128.3, 127.2. HRMS (ESI+) for $C_{14}H_{12}$ [M] +: calc. 180.0939; found: 180.0947.

Compound (8b):

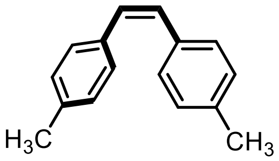

4-methylstyrene (210 µL, 1.6 mmol); Grubbs 2nd generation catalyst (6.8 mg, 0.008 mmol, 1 mol %); 4CzIPN (6.3 mg, 0.008 mmol, 1 mol %).

Purification (colorless oil, 110 mg, 0.53 mmol, 66 %).^{1}H NMR (300 MHz, $CDCl_3$) δ 7.18 (d, J = 9.0 Hz, 4H), 7.05 (d, J = 9.0 Hz, 4H), 6.53 (s, 2H), 2.33 (s, 6H).^{13}C NMR (75 MHz, $CDCl_3$) δ 136.9, 134.6, 129.7, 129.0, 128.9, 21.4. HRMS (ESI+) for $C_{16}H_{16}$ [M] +: calc. 208.1252; found: 208.1255.

Compound (8c):

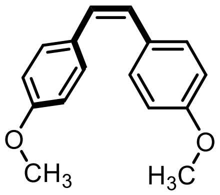

4-methoxystyrene (214 µL, 1.6 mmol); Grubbs 2nd generation catalyst (6.8 mg, 0.008 mmol, 1 mol %); 4CzIPN (6.3 mg, 0.008 mmol, 1 mol %).

Purification (Colorless oil, 79 mg, 0.33 mmol, 41%).^{1}H NMR (300 MHz, $CDCl_3$) δ 7.20 (d, *J* = 9.0 Hz, 4H), 6.77 (d, *J* = 9.0 Hz, 4H), 6.45 (s, 2H), 3.79 (s, 6H).^{13}C NMR (75 MHz, $CDCl_3$) δ 158.7, 130.2, 130.1, 128.5, 113.7, 55.3.

HRMS (ESI+) for $C_{16}H_{16}O_2$ [M] +: calc. 240.1150; found: 240.1157.

Compound (8d):

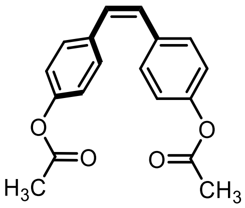

4-acetoxystyrene (245 µL, 1.6 mmol); Grubbs 2nd generation catalyst (6.8 mg, 0.008 mmol, 1 mol %); 4CzIPN (6.3 mg, 0.008 mmol, 1 mol %).

Purification (White solid,189 mg, 0.64 mmol, 80 %).^{1}H NMR (300 MHz, $CDCl_3$) δ 7.26 (d, *J* = 9.0 Hz, 4H), 6.96 (d, *J* = 9.0 Hz, 4H), 6.56 (s, 2H), 2.28 (s, 6H).^{13}C NMR (75 MHz, $CDCl_3$) δ 169.5, 149.8, 134.7, 130.0, 129.6, 121.6, 21.3.

HRMS (ESI+) for $C_{18}H_{16}O_4$ [M] +: calc. 296.1049; found: 296.1055.

Compound (8e):

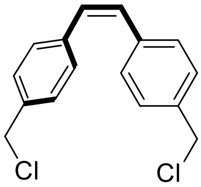

4-vinylbenzyl chloride (225 µL, 1.6 mmol); Grubbs 2nd generation catalyst (6.8 mg, 0.008 mmol, 1 mol %); 4CzIPN (6.3 mg, 0.008 mmol, 1 mol %).

Purification (White solid, 123 mg, 0.45 mmol, 56%).^{1}H NMR (300 MHz, $CDCl_3$) δ 7.28 – 7.22 (m, 8H), 6.60 (s, 2H), 4.56 (s, 4H).^{13}C NMR (75 MHz, $CDCl_3$) δ 137.4, 136.5, 130.3, 129.3, 128.7, 46.2.

HRMS (ESI+) for $C_{18}H_{14}Cl_2$ [M] +: calc. 276.0473; found: 276.0471.

Compound (8f):

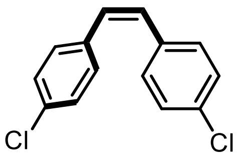

4-chlorostyrene (191 µL, 1.6 mmol); Grubbs 2nd generation catalyst (6.8 mg, 0.008 mmol, 1 mol %); 4CzIPN (6.3 mg, 0.008 mmol, 1 mol %).

Purification (Colorless oil, 121 mg, 0.49 mmol, 61%).^{1}H NMR (300 MHz, $CDCl_3$) δ 7.22 – 7.13 (m, 8H), 6.56 (s, 2H).^{13}C NMR (75 MHz, $CDCl_3$) δ 135.4, 133.2, 130.3, 129.8, 128.7. HRMS (ESI+) for $C_{14}H_{10}Cl_2$ [M] +: calc. 248.0160; found: 248.0168.

Compound (8g):

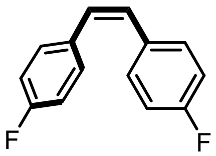

4-fluorostyrene (191 µL, 1.6 mmol); Grubbs 2nd generation catalyst (6.8 mg, 0.008 mmol, 1 mol %); 4CzIPN (6.3 mg, 0.008 mmol, 1 mol %).

Purification (Colorless oil, 102 mg,0.47 mmol, 59%).^{1}H NMR (300 MHz, $CDCl_3$) δ 7.22 – 7.17 (m, 4H), 6.96 – 6.91 (m, 4H), 6.55 (s, 2H). ^{19}F NMR (282 MHz, $CDCl_3$) δ -114.5. ^{13}C NMR (75 MHz, $CDCl_3$) δ 162.0 (d, J = 246.9 Hz),133.1 (d, J = 3.3 Hz), 130.6 (d, J = 7.9 Hz), 129.2 (d, J = 1.4 Hz), 115.4 (d, J = 21.3 Hz).

HRMS (ESI+) for $C_{14}H_{11}F_2$ [M] +: calc. 217.0829; found: 217.0836.

Compound (8h):

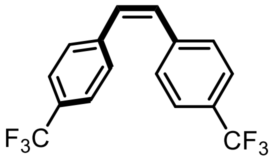

4-(trifluoromethyl)styrene (236 µL, 1.6 mmol) ; Grubbs 2nd generation catalyst (6.8 mg, 0.008 mmol, 1 mol %) ; 4CzIPN (6.3 mg, 0.008 mmol, 1 mol %).

Purification (White solid, 152 mg, 0.48 mmol, 60 %).^{1}H NMR (300 MHz, $CDCl_3$) δ 7.51 (d, *J* = 7.5 Hz, 4H), 7.32 (d, *J* = 7.5 Hz, 4H), 6.73 (s, 2H).^{19}F NMR (282 MHz, $CDCl_3$) δ - 62.6. ^{13}C NMR (75 MHz, $CDCl_3$) δ 140.3, 130.9, 129.6 (q, *J* = 32.6 Hz), 129.3, 125.6 (q, *J* = 3.8 Hz), 124.1 (q, *J* = 270.0 Hz).

HRMS (ESI+) for $C_{16}H_{10}F_6$ [M] +: calc. 316.0687; found: 316.0694.

Compound (8i):

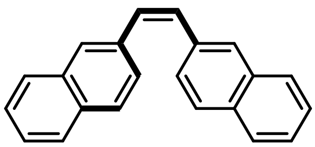

2-vinylnaphtalene (247 µL, 1.6 mmol); Grubbs 2nd generation catalyst (6.8 mg, 0.008 mmol, 1 mol %); 4CzIPN (6.3 mg, 0.008 mmol, 1 mol %).

Purification (White solid, 115 mg, 0.41 mmol, 51 %).^{1}H NMR (300 MHz, $CDCl_3$) δ 7.79 – 7.62 (m, 8H), 7.47 – 7.36 (m, 6H), 6.85 (s, 2H).^{13}C NMR (75 MHz, $CDCl_3$) δ 135.0, 133.6, 132.8, 130.7, 128.3, 128.1, 127.8, 127.7, 127.1, 126.2, 126.1.

HRMS (ESI+) for $C_{22}H_{16}$ [M] +: calc. 280.1252; found: 280.1266.

Compound (8j):

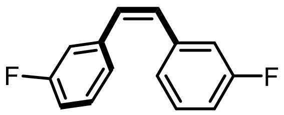

3-fluorostyrene (190 µL, 1.6 mmol); Grubbs 2nd generation catalyst (6.8 mg, 0.008 mmol, 1 mol %); 4CzIPN (6.3 mg, 0.008 mmol, 1 mol %).

Purification (Colorless oil, 105 mg, 0.48 mmol, 61%).^{1}H NMR (300 MHz, $CDCl_3$) δ 7.23 – 7.17 (m, 2H), 7.00 (dt, *J* = 7.0, 1.2 Hz, 2H), 6.94 – 6.89 (m, 4H), 6.60 (s, 2H).^{19}F NMR (282 MHz, $CDCl_3$) δ -113.2. ^{13}C NMR (75 MHz, $CDCl_3$) δ 162.9 (d, *J* = 244.1 Hz), 139.1 (d, *J* = 7.8 Hz), 130.3 (d, *J* = 2.4 Hz), 130.0 (d, *J* = 8.2 Hz), 124.8 (d, *J* = 2.9 Hz), 115.7 (d, *J* = 21.7 Hz), 114.5 (d, *J* = 21.2 Hz).

HRMS (ESI+) for $C_{14}H_{10}F_2$[M] +: calc. 217.0829; found: 217.0831.

Compound (8k):

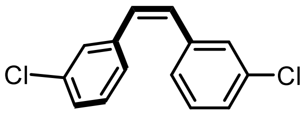

3-chlorostyrene (203 µL, 1.6 mmol); Grubbs 2nd generation catalyst (6.8 mg, 0.008 mmol, 1 mol %); 4CzIPN (6.3 mg, 0.008 mmol, 1 mol %).

Purification (Colorless oil, 137 mg, 0.55 mmol, 69 %).^{1}H NMR (300 MHz, $CDCl_3$) δ 7.23 – 7.13 (m, 6H), 7.10 – 7.07 (m, 2H), 6.58 (s, 2H).^{13}C NMR (75 MHz, $CDCl_3$) δ 138.6, 134.4, 130.2, 129.7, 129.0, 127.6, 127.1.

HRMS (ESI+) for $C_{14}H_{10}Cl_2$ [M] +: calc. 249.0238; found: 249.0244.

2-2 Procedure for metathesis reaction of styrene derivatives with cis-2-butene-1,4-diacetate (**8l-8o**):

In a flame-dried quartz Schlenk tube, styrene (1.0 eq) was dissolved in dichloromethane to give concentration (0.2 M) and the cis-2-butene-1,4-diacetate (2.4 eq), *fac*-Ir(ppy)$_3$ (5 mol %) and 2nd generation catalyst (5 mol %) were added. The reaction mixture was stirred at 40 °C for 24h under argon and irradiated at 420 nm. The solvent was removed under reduced pressure and the residue was purified by flash chromatography on silica gel (pentane/Ethyl acetate 95/5).

Compound (8l):

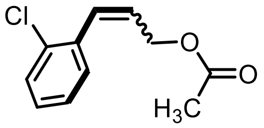

cis-2-butene-1,4-diacetate (153 µL, 0.96 mmol); 2-chlorostyrene (51 µL, 0.4 mmol); *fac*-Ir(ppy)$_3$ (13 mg, 0.02 mmol, 5 mol%); 2nd generation catalyst (17 mg, 0.02 mmol, 5 mol %).

Purification (Colorless oil, 63 mg, 0.3 mmol, 75% (*Z*:*E* = 92:8 in isolated product).

***Z* isomer (major)**: ^{1}H NMR (300 MHz, CDCl$_3$) δ 7.40 - 7.38 (m, 1H), 7.26 - 7.22 (m, 3HZ + 3H*E*), 6.78 (dt, *J* = 11.4, 1.6 Hz, 1H), 5.93 (dt, *J* = 11.4, 6.6 Hz, 1H), 4.71 (dd, *J* = 6.6, 1.6 Hz), 2.07 (s, 3H). ^{13}C NMR (75 MHz, CDCl$_3$) δ 171.0, 134.3, 133.8, 130.6, 130.5, 129.7, 129.1, 127.3, 126.7, 61.4, 21.1.

***E* isomer (minor):** ^{1}H NMR (300 MHz, CDCl$_3$) δ 7.54 - 7.51 (m, 1H), 7.26 - 7.22 (m, 3HZ + 3H*E*), 7.04 (dt, *J* = 16.0, 1.6 Hz, 1H), 6.27 (dt, *J* = 16.0, 6.9 Hz, 1H), 4.76 (dd, *J* = 6.9, 1.6 Hz), 2.11 (s, 3H). ^{13}C NMR (75 MHz, CDCl$_3$) δ 171.0, 134.5, 133.4, 130.1, 129.9, 129.2, 127.1, 127.0, 126.2, 65.0, 21.1.

HRMS (ESI+) for $C_{11}H_{11}O_2Cl$ [M] +: calc. 210.0448; found: 210.0444.

Compound (8m):

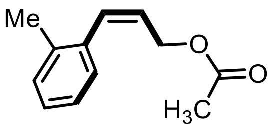

cis-2-butene-1,4-diacetate (150 µL, 0.96 mmol); 2-methylstyrene (52 µL, 0.4 mmol); *fac*-Ir(ppy)$_3$ (13 mg, 0.02 mmol, 5 mol %); 2nd generation catalyst (17 mg, 0.02 mmol, 5 mol%).

Purification (Colorless oil, 38 mg, 0.2 mmol, 50 % (*Z* isomer).^{1}H NMR (300 MHz, $CDCl_3$) δ 7.20 - 7.08 (m, 4H), 6.73 (dt, *J* = 11.4, 1.6 Hz, 1H), 5.86 (dt, *J* = 11.4, 6.8 Hz, 1H), 4.70 (dd, *J* = 6.8, 1.6 Hz, 2H), 2.27 (s, 3H), 2.06 (s, 3H). ^{13}C NMR (75 MHz, $CDCl_3$) δ 171.0, 136.4, 135.2, 132.7, 130.1, 129.1, 127.9, 125.9, 125.8, 61.6, 21.1, 20.0. HRMS (ESI+) for $C_{12}H_{14}O_2$ [M] +: calc. 190.0994; found: 190.1002.

Compound (8n):

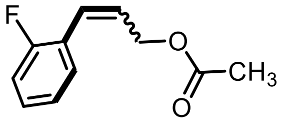

cis-2-butene-1,4-diacetate (150 µL, 0.96 mmol); 2-fluorostyrene (48 µL, 0.4 mmol); *fac*-Ir(ppy)$_3$ (13 mg, 0.02 mmol, 5 mol %); 2nd generation catalyst (17 mg, 0.02 mmol, 5 mol %).

Purification (Colorless oil, 68 mg, 0.35 mmol, 88 % (*Z*:*E* = 78:22 in isolated product).

***Z* isomer (major)**: ^{1}H NMR (300 MHz, $CDCl_3$) δ 7.36 - 7.27 (m, 1H*Z* + 1H*E*), 7.01 – 6.91 (m, 3H), 6.62 (d, *J* = 12.1 Hz, 1H), 5.85 (dt, *J*= 12.1, 6.6 Hz, 1H), 4.82 (dd, *J* = 6.6, 1.7 Hz, 2H), 2.09 (s, 3H). ^{19}F NMR (282 MHz, $CDCl_3$) δ -113.1. ^{13}C NMR (75 MHz, $CDCl_3$) δ 171.0, 160.0 (d, *J* = 240.1 Hz); 130.5 (d, *J* = 3.5 Hz), 129.7 (d, *J* = 8.3 Hz), 127.9, 125.7 (d, *J* = 3.6 Hz), 124.0 (d, *J* = 3.9 Hz), 123.8 (d, *J* = 14.6 Hz), 115.6 (d, *J* = 22.0 Hz), 61.7, 21.0.

***E* isomer (minor):** ^{1}H NMR (300 MHz, $CDCl_3$) δ 7.36 - 7.27 (m, 1H*Z* + 1H*E*), 7.16 - 7.07 (m, 3H), 6.67 (d, *J* = 15.9 Hz, 1H), 6.29 (dt, *J* = 15.9, 6.3 Hz, 1H), 4.73 (dd, *J* = 6.3, 1.4 Hz, 2H), 2.11 (s, 3H). ^{19}F NMR (282 MHz, $CDCl_3$) δ -113.4. ^{13}C NMR (75 MHz, $CDCl_3$) δ 171.0, 160.4 (d, *J* = 240.1 Hz), 129.5 (d, *J* = 9.8 Hz), 127.7 (d, *J* = 3.5 Hz), 126.5 (d, *J* = 3.5 Hz), 126.0 (d, *J* = 5.0 Hz), 124.2 (d, *J* = 3.8 Hz), 129.4, 115.9 (d, *J* = 21.9 Hz), 65.2, 21.1

HRMS (ESI+) for $C_{11}H_{11}O_2F$ [M] +: calc. 194.0743; found: 194.0759.

Compound (8o):

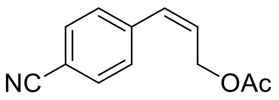

Cis-2-butene-1,4-diacetate (150 µL, 0.96 mmol); 4-cyanostyrene (52 µL, 0.40 mmol); *fac*-Ir(ppy)$_3$ (13 mg, 0.02 mmol, 5 mol %); 2nd generation catalyst (17 mg, 0.02 mmol, 5 mol %).

Purification (Colorless oil, 33 mg, 0.16 mmol, 42 % (Z isomer). ^{1}H NMR (300 MHz, CDCl$_3$) δ 7.61 (d, *J* = 8.3 Hz, 2H), 7.46 (d, *J* = 8.3 Hz, 2H), 6.65 (d, *J* = 12.6 Hz, 1H), 6.41 (dt, *J* = 12.6, 1.6 Hz, 1H), 4.76 (dd, *J* = 6.6, 1.6 Hz), 2.12 (s, 3H). ^{13}C NMR (75 MHz, CDCl$_3$) δ 170.8, 140.8, 132.6, 132.5, 132.0, 127.5, 127.2, 111.5, 64.5, 21.0.

2-3-Procedure for metathesis reaction of styrene derivatives with 5-hexenyl-1-acetate (**8p-8q**):

In flame dried flask, styrene (3.0 eq), *fac*-Ir(ppy)$_3$ (5 mol %) and 2nd generation catalyst (5 mol%) were added to a solution of 5-hexenyl-1-acetate (1.0 eq) in dichloromethane (0.18 M). The reaction mixture was stirred at 40 °C for 24h under argon and irradiated at 420 nm. The solvent was removed under reduced pressure and the residue was purified by flash chromatography on silica gel (pentane/Ethyl acetate 95/5) to provide the compound.

Compound (8p):

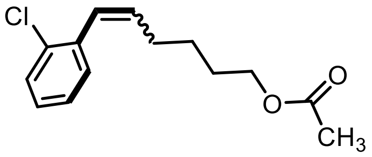

5-hexenyl-1-acetate (72 µL, 0.45 mmol), 2-fluorostyrene (174 µL, 1.36 mmol) and *fac*-Ir(ppy)$_3$ (15 mg, 0.022 mmol, 5 mol %) 2nd generation catalyst (19 mg, 0.022 mmol, 5 mol %).

Purification (colorless oil, 84 mg, 0.33 mmol, 74% (*Z*:*E* = 92:8 in isolated product).

***Z* isomer (major):** ^{1}H NMR (300 MHz, CDCl$_3$) δ 7.39 – 7.36 (m, 1H), 7.28 – 7.13 (m, 3H), 6.52 (d, *J* = 12.0 Hz, 1H), 5.77 (dt, *J* = 12.0, 7.4 Hz, 1H), 4.02 (t, *J* = 6.6 Hz, 2H), 2.31 – 2.17

(m, 2H*Z* + 1H*E*), 2.02 (s, 3H), 1.81 – 1.43 (m, 4H*Z* + 4H*E*).^{13}C NMR (75 MHz, $CDCl_3$) δ 171.3, 135.8, 133.7, 132.7, 130.6, 129.5, 128.2, 126.8, 126.3, 64.4, 28.3, 28.1, 26.1, 21.1.

***E* isomer (minor):** ^{1}H NMR (300 MHz, $CDCl_3$) δ 7.51 – 7.48 (m, 1H), 7.34 – 7.31 (m, 3H), 6.77 (d, *J* = 15.0 Hz, 1H), 6.20 (dt, *J* = 15.0, 7.2 Hz, 1H), 4.13 (t, *J* = 6.6 Hz, 2H), 2.56 – 2.49 (m, 1H), 2.31 – 2.17 (m, 2H*Z* + 1H*E*), 2.05 (s. 3H), 1.81 – 1.43 (m, 4H*Z* + 4H*E*)).^{13}C NMR (75 MHz, $CDCl_3$) δ 171.2, 135.7, 133.7, 133.3, 130.5, 129.7, 128.3, 127.4, 126.7, 63.9, 28.6, 25.7, 24.9, 21.0.

HRMS (ESI+) for $C_{14}H_{17}O_2Cl$ [M] +: calc. 252.0917; found: 252.0930.

Compound (8q):

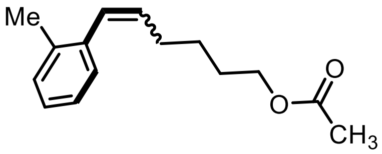

5-hexenyl-1-acetate (70 µL, 0.45 mmol), 2-methylstyrene (176 µL, 1.36 mmol) and *Fac*-Ir(ppy)$_3$ (15 mg, 0.022 mmol, 5 mol%) 2nd generation catalyst (19 mg, 0.022mmol, 5 mol%).

Purification (Colorless oil, 84 mg, 0.36 mmol, 80 % (*Z*:*E* = 94:6 in isolated product)).

***Z* isomer (major) :** ^{1}H NMR (300 MHz, $CDCl_3$) δ 7.20 – 7.13 (m, 4H*Z* + 4H*E*), 6.46 (dt, *J* = 11.4, 1.8 Hz, 1H), 5.69 (dt, *J* = 11.4, 7.4 Hz, 1H), 4.02 (t, *J* = 6.6 Hz, 2H), 2.25 (s, 3H), 2.23 – 2.15 (m, 2H), 2.03 (s. 3H), 1.81 – 1.38 (m, 4H*Z* + 4H*E*).^{13}C NMR (75 MHz, $CDCl_3$) δ 171.2, 136.8, 136.4, 132.2, 129.9, 129.1, 128.6, 126.9, 125.4, 64.5, 28.3, 28.0, 26.3, 21.1, 20.0.

***E* isomer (minor):** ^{1}H NMR (300 MHz, $CDCl_3$) δ 7.21 – 7.12 (m, 4H*E* + 4H*Z*), 6.50 (d, *J* = 15.7 Hz, 1H), 6.07 (dt, *J* = 15.7, 6.9 Hz, 1H), 4.03 (t, *J* = 6.6 Hz, 2H), 2.53 – 2.43 (m, 1H), 2.34 (s, 3H), 2.06 (s. 3H), 1.81 – 1.38 (m, 4H*Z* + 4H*E*).^{13}C NMR (75 MHz, $CDCl_3$) δ 171.1,136.6, 136.4, 131.2, 130.3, 129.3, 129.0, 127.0, 125.6, 63.9, 29.8, 28.7, 24.7, 21.0, 20.0.

HRMS (ESI+) for $C_{15}H_{20}O_2$ [M] +: calc. 232.1461; found: 232.1463.

2-4-General procedure for metathesis reaction (Cross metathesis **8r-8zf**):

A flame-dried quartz Schlenk tube was charged under argon with styrene (1.0 eq) and dichloromethane (0.05 M). Next, alkyl acrylate (2.0 to 5.0 eq), Grubbs 2nd generation catalyst

(2 mol %) and 4CzIPN (5 mol %) were added in one portion. The Schlenk tube was closed with a cap and then it stirred at 40 °C under argon for 24h and irradiated at 420 nm. The solvent was removed under reduced pressure and the residue was purified by flash chromatography (silica gel, pentane/Ethyl acetate 98/2) to provide the compound.

Compound (8r):

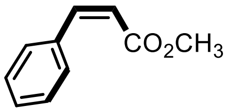

Styrene (80 µL, 0.7 mmol); Methyl acrylate (317 µL, 3.5 mmol, 5.0 eq) Grubbs 2nd generation catalyst (12 mg, 0.014 mmol, 2 mol %); 4CzIPN (27.7 mg, 0.035 mmol, 5 mol %).

Purification (Colorless solid, 64 mg, 0.39 mmol, 56% (*Z* isomer)). ^{1}H NMR (300 MHz, $CDCl_3$) δ 7.61 – 7.57 (m, 2H), 7.37 – 7.34 (m, 3H), 6.96 (d, J = 12.6 Hz, 1H), 5.96 (d, J = 12.6 Hz, 1H), 3.71 (s, 3H). ^{13}C NMR (75 MHz, $CDCl_3$) δ 166.8, 143.6, 134.9, 129.8, 129.2, 128.2, 119.4, 51.5.

HRMS (ESI+) for $C_{10}H_{11}O_2$ [M] +: calc. 163.0759; found: 163.0764.

Compound (8s):

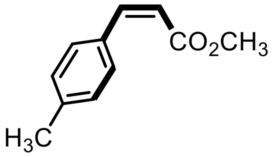

4-methylstyrene (93 µL, 0.7 mmol); Methyl acrylate (317 µL, 3.5 mmol, 5 eq); Grubbs 2nd generation catalyst (12 mg, 0.014 mmol, 2 mol %) ; 4CzIPN (27.7 mg, 0.035 mmol, 5 mol %).

Purification (Colorless oil, 62 mg, 0.35 mmol, 50 % (*Z* isomer)). ^{1}H NMR (300 MHz, $CDCl_3$) δ 7.53 (d, J = 9.0 Hz, 2H), 7.17 (d, J = 9.0 Hz, 2H), 6.92 (d, J = 12.7 Hz, 1H), 5.90 (d, J = 12.7 Hz, 1H), 3.72 (s, 3H), 2.36 (s, 3H). ^{13}C NMR (75 MHz, $CDCl_3$) δ 166.9, 143.7, 139.5, 132.0, 130.1, 128.9, 118.4, 51.5, 21.6.

HRMS (ESI+) for $C_{11}H_{12}O_2$ [M] +: calc. 176.0837; found: 176.0834.

Compound (8t):

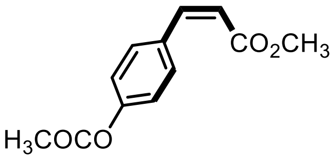

4-acetoxystyrene (107 µL, 0.7 mmol); Methyl acrylate (317 µL, 3.5 mmol, 5 eq); Grubbs 2nd generation catalyst (12 mg, 0.014 mmol, 2 mol %); 4CzIPN (27.7 mg, 0.035 mmol, 5 mol %).

Purification (White solid, 77 mg, 0.35 mmol, 50% (*Z* isomer)). ^{1}H NMR (300 MHz, $CDCl_3$) δ 7.67 (d, *J* = 9.0 Hz, 2H), 7.09 (d, *J* = 9.0 Hz, 2H), 6.91 (d, *J* = 12.7 Hz, 1H), 5.95 (d, *J* = 12.7 Hz, 1H), 3.71 (s, 3H), 2.30 (s, 3H). ^{13}C NMR (75 MHz, $CDCl_3$) δ 169.3, 166.6, 151.2, 142.6, 132.4, 131.4, 121.3, 119.4, 51.5, 21.3.

HRMS (ESI+) for $C_{12}H_{13}O_4$ [M] +: calc. 221.0814; found: 221.0817.

Compound (8u):

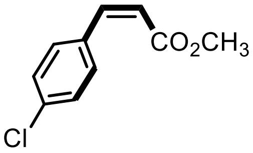

4-chlorostyrene (84 µL, 0.7 mmol); Methyl acrylate (317 µL, 3.5 mmol, 5 eq); Grubbs 2nd generation catalyst (12 mg, 0.014 mmol, 2 mol %); 4CzIPN (27.7 mg, 0.035 mmol, 5 mol %).

Purification (Yellow oil, 68 mg, 0.35 mmol, 50 % (*Z* isomer)). ^{1}H NMR (300 MHz, $CDCl_3$) δ 7.55 (d, *J* = 9.0 Hz, 2H), 7.32 (d, *J* = 9.0 Hz, 2H), 6.88 (d, *J* = 12.7 Hz, 1H), 5.97 (d, *J* = 12.7 Hz, 1H), 3.71 (s, 3H).^{13}C NMR (75 MHz, $CDCl_3$) δ 166.5, 142.4, 135.2, 133.3, 131.3, 128.4, 119.9, 51.6.

HRMS (ESI+) for $C_{10}H_{10}O_2Cl$ [M] +: calc. 197.0369; found: 197.0367.

Compound (8v):

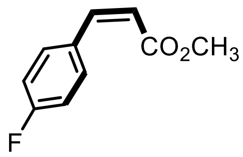

4-fluorostyrene (84 µL, 0.7 mmol); Methyl acrylate (317 µL, 3.5 mmol, 5 eq); Grubbs 2nd generation catalyst (12 mg, 0.014 mmol, 2 mol %); 4CzIPN (27.7 mg, 0.035 mmol, 5 mol %).

Purification (Colorless oil, 91 mg, 0.5 mmol, 72% (*Z* isomer)). ^{1}H NMR (300 MHz, $CDCl_3$) δ 7.67 – 7.61 (m, 2H), 7.08 – 7.00 (m, 2H), 6.89 (d, *J* = 12.7 Hz, 1H), 5.93 (d, *J* = 12.7 Hz, 1H), 3.72 (s, 3H).^{19}F NMR (282 MHz, $CDCl_3$) δ -111.3. ^{13}C NMR (75 MHz, $CDCl_3$) δ 166.6, 163.1 (d, *J* = 250.0 Hz), 142.6, 132.2 (d, *J* = 8.3 Hz), 130.9 (d, 3.4 Hz), 119.0 (d, *J* = 1.7 Hz), 115.1 (d, *J* = 21.5 Hz), 51.5.

HRMS (ESI+) for $C_{10}H_{10}O_2F$ [M] +: calc. 181.0665; found: 181.0663.

Compound (8w):

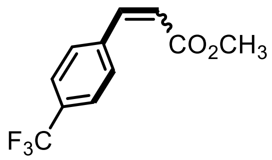

4-(trifluoromethyl)styrene (104 µL, 0.7 mmol); Methyl acrylate (317 µL, 3.5 mmol, 5.0 eq); Grubbs 2nd generation catalyst (12 mg, 0.014 mmol, 2 mol %); 4CzIPN (27.7 mg, 0.035 mmol, 5 mol %).

Purification (Colorless oil, 138 mg, 0.6 mmol, 86% (*Z*:*E* = 82:18 in isolated product).

***Z* isomer (major):** ^{1}H NMR (300 MHz, $CDCl_3$) δ 7.66 – 7.59 (m, 4H*Z* + 4H*E*), 6.99 (d, *J* = 12.6 Hz, 1H), 6.07 (d, *J* = 12.6 Hz, 1H), 3.71 (s, 3H). ^{19}F NMR (282 MHz, $CDCl_3$) δ -62.9 ^{13}C NMR (75 MHz, $CDCl_3$) δ 166.2, 142.0, 138.4, 130.7 (q, 32.0 Hz), 129.8, 125.1 (q, *J* = 3.8 Hz), 121.6, 120.9 (q, 270.0 Hz), 51.7.

***E* isomer (minor):** ^{1}H NMR (300 MHz, $CDCl_3$) δ 7.70 (d, *J* = 16.0 Hz, 1H), 7.68 – 7.59 (m, 4H*Z* + 4H*E*), 6.51 (d, *J* = 16.0 Hz, 1H), 3.83 (s, 3H). ^{19}F NMR (282 MHz, $CDCl_3$) δ -62.8. ^{13}C NMR (75 MHz, $CDCl_3$) δ 167.0, 143.1, 137.9, 131,9 (q, 32 Hz), 128.3, 126.0 (q, *J* = 3.8 Hz), 120.9 (q, 270 Hz), 120.5, 52.1.

HRMS (ESI+) for $C_{11}H_{10}O_2F_3$ [M] +: calc. 231.0633; found: 231.0623.

Compound (8x):

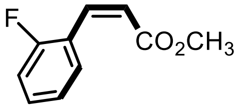

2-fluorostyrene (83 µL, 0.7 mmol); Methyl acrylate (127 µL, 1.4 mmol, 2eq); Grubbs 2nd generation catalyst (12.0 mg, 0.014 mmol, 2 mol %); 4CzIPN (27.7 mg, 0.035 mmol, 5 mol %).

Purification (Yellow oil, 64 mg, 0.35 mmol, 51% (*Z* isomer)). ^{1}H NMR (300 MHz, $CDCl_3$) δ 7.63 – 7.58 (m, 1H), 7.36 – 7.28 (m, 1H), 7.15 – 7.02 (m, 3H), 6.08 (d, *J* = 12.5 Hz, 1H), 3.70 (s, 3H). ^{19}F NMR (282 MHz, $CDCl_3$) δ -114.1. ^{13}C NMR (75 MHz, $CDCl_3$) δ 166.3, 160.3 (d, 249.7 Hz), 136.0 (d, *J* = 3.9 Hz), 130.9 (d, *J* = 2.5 Hz), 130.8 (d, J = 8,5 Hz), 123.6 (d, *J* = 3.7 Hz), 123.0 (d, *J* = 14,8 Hz), 121.8 (d, *J* = 1.5 Hz), 115.3 (d, *J* = 21.7 Hz), 51.6.

HRMS (ESI+) for $C_{10}H_{10}O_2F$ [M] +: calc. 181.0665; found: 181.0663.

Compound (8y):

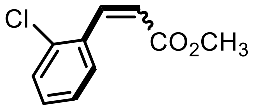

2-chlorostyrene (89 µL, 0.7 mmol); Methyl acrylate (127 µL, 1.4 mmol, 2.0 eq); Grubbs 2nd generation catalyst (12.0 mg, 0.014 mmol, 2 mol %); 4CzIPN (27.7 mg, 0.035 mmol, 5 mol %).

Purification (Colorless oil, 103 mg, 0.52 mmol, 75 % (*Z*:*E* = 92:8 in isolated product)).

***Z* isomer (major):** ^{1}H NMR (300 MHz, $CDCl_3$) δ 7.52 – 7.49 (m, 1H), 7.41 – 7.37 (m, 1H*E* + 1H*Z*), 7.32 – 7.21 (m, 2H*Z* + 2H*E*), 7.16 (d, *J* = 12.2 Hz, 1H), 6.09 (d, *J* = 12.2 Hz, 1H), 3.66 (s, 3H). ^{13}C NMR (75 MHz, $CDCl_3$) δ 166.2, 140.9, 133.9, 130.9, 130.0, 129.3, 127.2, 126.2, 121.5, 51.6.

***E* isomer (minor):** ^{1}H NMR (300 MHz, $CDCl_3$) δ 8.10 (d, *J* = 16.0 Hz, 1H), 7.63 – 7.60 (m, 1H), 7.41 – 7.37 (m, 1H*E* + 1H*Z*), 7.32 – 7.21 (m, 2H*Z* + 2H*E*), 6.44 (d, *J* = 16.0 Hz, 1H),

3.83 (s, 3H). ^{13}C NMR (75 MHz, $CDCl_3$) δ 166.2, 140.8, 133.4, 131.2, 130.3, 127.8, 127.2, 124.6, 120.6, 52.0.

HRMS (ESI+) for $C_{10}H_{10}O_2Cl$ [M] +: calc. 197.0369; found: 197.0371.

Compound (8z):

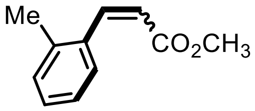

2-methylstyrene (91 µL, 0.7 mmol); Methylacrylate (127 µL, 1.4 mmol, 2eq) ; Grubbs 2nd generation catalyst (12.0 mg, 0.014 mmol, 2 mol %) ; 4CzIPN (27.7 mg, 0.035 mmol, 5 mol %).

Purification (Colorless oil, 89 mg, 0.50 mmol, 72 % (*Z*:*E* = 90:10 in isolated product).

***Z* isomer (major):** ^{1}H NMR (300 MHz, $CDCl_3$) δ 7.33 (d, *J* = 6.9 Hz, 1H), 7.23 – 7.11 (m, 4H*Z* + 3H*E*), 6.04 (d, *J* = 12.5 Hz, 1H), 3.64 (s, 3H), 2.29 (s, 3H). ^{13}C NMR (75 MHz, $CDCl_3$) δ 166.5, 143.5, 135.9, 134.9, 129.8, 128.9, 128.7, 125.4, 120.6, 51.4, 20.0.

***E* isomer (minor):** ^{1}H NMR (300 MHz, $CDCl_3$) δ 7.99 (d, *J* = 16.0 Hz, 1H), 7.57 – 7.52 (m, 1H), 7.23 – 7.11 (m, 4HZ + 3H*E*), 6.37 (d, *J* = 16.0 Hz, 1H), 3.82 (s, 3H), 2.44 (s, 3H). ^{13}C NMR (75 MHz, $CDCl_3$) δ 167.6, 142.7, 137.8, 133.5, 130.9, 130.2, 126.5,126.4, 119.0, 51.8, 19.9.

HRMS (ESI+) for $C_{11}H_{13}O_2$ [M] +: calc. 177.0916; found: 177.0917.

Compound (8za):

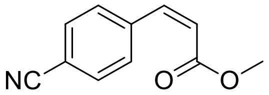

4-cyanostyrene (90 µL, 0.7 mmol); Methylacrylate (127 µL, 1.4 mmol, 2eq) ; Grubbs 2nd generation catalyst (12.0 mg, 0.014 mmol, 2 mol %) ; 4CzIPN (27.7 mg, 0.035 mmol, 5 mol %).

Purification (Yellow solid, 77 mg, 0.41 mmol, 59 % (*Z*:*E* = 74:26 in isolated product).

***Z* isomer (major):** ^{1}H NMR (300 MHz, $CDCl_3$) δ 7.69 – 7.55 (m, 4H*Z* + 5H*E*), 6.97 (d, *J* = 12.5 Hz, 1H), 6.09 (d, *J* = 12.5 Hz, 1H), 3.71 (s, 3H). ^{13}C NMR (75 MHz, $CDCl_3$) δ 165.9, 141.5, 139.5, 131.9, 130.1, 128.5, 122.4, 112.4, 51.8.

***E* isomer (minor):** ^{1}H NMR (300 MHz, $CDCl_3$) δ 7.69 – 7.55 (m, 4H*Z* + 5H*E*), 6.52 (d, *J* = 12.5 Hz, 1H), 3.82 (s, 3H). ^{13}C NMR (75 MHz, $CDCl_3$) δ 170.0, 142.5, 138.8, 132.8, 129.9, 122.4, 121.5, 118.8, 52.2.

Compound (8zb):

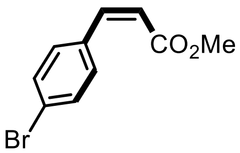

4-bromostyrene (92 µL, 0.7 mmol); Methyl acrylate (317 µL, 3.5 mmol, 5 eq); Grubbs 2nd generation catalyst (12 mg, 0.014 mmol, 2 mol %); 4CzIPN (27.7 mg, 0.035 mmol, 5 mol %).

Purification (Yellow oil, 102 mg, 0.43 mmol, 61 % (Z isomer)). ^{1}H NMR (300 MHz, $CDCl_3$) δ 7.48 (s, 4H), 6.87 (d, *J* = 12.6 Hz, 1H), 5.97 (d, *J* = 12.6 Hz, 1H), 3.71 (s, 3H). ^{13}C NMR (75 MHz, $CDCl_3$) δ 166.4, 142.4, 133.7, 131.5, 131.3, 123.5, 120.0, 51.6.

HRMS (ESI+) for $C_{10}H_{10}O_2Br$ [M] +: calc. 240.9864; found: 240.9865.

Compound (8zc):

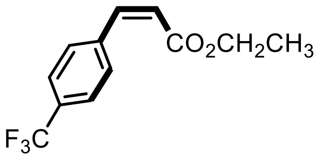

4-(trifluoromethyl)styrene (104 µL, 0.7 mmol) ; Ethyl acrylate (372 µL, 3.5 mmol, 5 eq); Grubbs 2nd generation catalyst (12.0 mg, 0.014 mmol, 2 mol %); 4CzIPN (27.7 mg, 0.035 mmol, 5 mol %).

Purification (Yellow liquid, 110 mg, 0.45 mmol, 66 % (*Z* isomer)). ^{1}H NMR (300 MHz, $CDCl_3$) δ 7.65 – 7.58 (m, 4H), 6.97 (d, *J* = 12.4 Hz, 1H), 6.06 (d, *J* = 12.4 Hz, 1H), 4.17 (q, *J* = 7.1 Hz, 2H), 1.24 (t, *J* = 7.1 Hz, 3H). ^{19}F NMR (282 MHz, $CDCl_3$) δ - 62.8. ^{13}C NMR (75

MHz, $CDCl_3$) δ 165.8, 141.6, 138.6, 130.6 (q, *J* = 32.5 Hz), 129.8, 125.0 (q, *J* = 3.8 Hz), 124.1 (q, *J* = 270.0 Hz), 122.2, 60.7, 14.1.

HRMS (ESI+) for $C_{12}H_{12}O_2F_3$ [M] +: calc. 245.0789; found: 245.0781.

Compound (8zd):

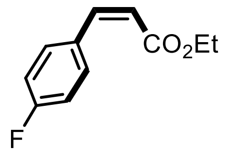

4-fluorostyrene (84 µL, 0.7 mmol); Ethyl acrylate (372 µL, 3.5 mmol, 5 eq); Grubbs 2nd generation catalyst (12 mg, 0.014 mmol, 2 mol %); 4CzIPN (27.7 mg, 0.035 mmol, 5 mol %).

Purification (Colorless oil, 92 mg, 0.47 mmol, 68 % (*Z* isomer)).^{1}H NMR (300 MHz, $CDCl_3$) δ 7.67 – 7.60 (m, 2H), 7.07 – 6.99 (m, 2H), 6.88 (d, *J* = 12.0 Hz, 1H), 5.93 (d, *J* = 12.0 Hz, 1H), 4.18 (q, *J* = 7.2 Hz, 2H), 1.26 (t, *J* = 7.14 Hz, 3H). ^{19}F NMR (282 MHz, $CDCl_3$) δ -111.4. ^{13}C NMR (75 MHz, $CDCl_3$) δ 166.2, 163.3 (d, *J* = 249.9 Hz), 142.3, 132.1 (d, *J* = 8.3 Hz), 131.0 (d, *J* = 3.3 Hz), 119.7 (d, *J* = 1.6 Hz), 115.1 (d, *J* = 21.6 Hz), 60.5, 14.3.

HRMS (ESI+) for $C_{11}H_{12}O_2F$ [M] +: calc. 195.0821; found: 195.0826.

Compound (8ze):

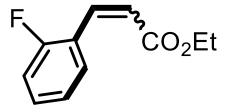

2-fluorostyrene (84 µL, 0.7 mmol); Ethyl acrylate (150 µL, 1.4 mmol, 2 eq); Grubbs 2nd generation catalyst (12.0 mg, 0.014 mmol, 2 mol %); 4CzIPN (27.7 mg, 0.035 mmol, 5 mol %).

Purification (Colorless solid, 82 mg, 0.42 mmol, 60% (*Z*:*E* = 84:16 in isolated product).

***Z* isomer (major):** ^{1}H NMR (300 MHz, $CDCl_3$) δ 7.62 – 7.51 (m, 1H*Z* + 1H*E*), 7.37 – 7.27 (m, 1H*Z* + 1H*E*), 7.19 – 7.01 (m, 3H*Z* + 2H*E*), 6.08 (d, *J* = 12.6 Hz, 1H), 4.16 (q, *J* = 7.2 Hz, 2H), 1.22 (t, *J* = 7.2 Hz, 3H). ^{19}F NMR (282 MHz, $CDCl_3$) δ -114.1. ^{13}C NMR (75 MHz, $CDCl_3$) δ 165.9, 160.3 (d, *J* = 248.9 Hz), 135.6 (d, *J* = 3.8 Hz), 131.0 (d, *J* = 2.7 Hz), 130.7

(d, J = 8.54 Hz), 123.6 (d, J = 3.6 Hz), 122.4 (d, J = 1.5 Hz), 121.0 (d, J = 6.6 Hz), 115.2 (d, J = 22.1 Hz), 60.5, 14.1.

***E* isomer (minor):** ^{1}H NMR (300 MHz, $CDCl_3$) δ 7.81 (d, J = 16.0 Hz, 1H), 7.62 – 7.54 (m, 1HZ + 1H*E*), 7.37 – 7.28 (m, 1HZ + 1H*E*), 7.19 – 7.01 (m, 3HZ + 2H*E*), 6.54 (d, J = 16.0 Hz, 1H), 4.28 (q, J =7.2 Hz, 2H), 1.34 (t, J =7.2 Hz, 3H). ^{19}F NMR (282 MHz, $CDCl_3$) δ -114.4. ^{13}C NMR (75 MHz, $CDCl_3$) δ 166.9, 161.1 (d, J = 248.9 Hz), 137.3 (d, J = 2.3 Hz), 131.7 (d, J = 8.8 Hz), 129.2 (d, J = 3.0 Hz), 124.5 (d, J = 3.8 Hz), 123.2 (d, J = 13.4 Hz), 121.0 (d, J = 6.6 Hz), 116.3 (d, J = 22.1 Hz), 60.7, 14.4.

HRMS (ESI+) for $C_{10}H_{10}O_2F$ [M] +: calc. 195.0821; found: 195.0834.

Compound (8zf):

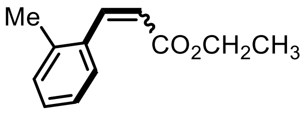

2-methylstyrene (90.5 µL, 0.7 mmol); Ethyl acrylate (150 µL, 1.4 mmol, 2 eq); Grubbs 2nd generation catalyst (12.0 mg, 0.014 mmol, 2 mol %); 4CzIPN (27.7 mg, 0.035 mmol, 5 mol %).

Purification (Colorless liquid, 98 mg, 0.52 mmol, 74% (*Z*:*E* = 92:8 in isolated product)).

***Z* isomer (major):** ^{1}H NMR (300 MHz, $CDCl_3$) δ 7.31 (d, J = 7.6 Hz, 1H), 7.23 – 7.10 (m, 4H), 6.02 (d, J = 12.0 Hz, 1H), 4.09 (q, J = 7.2 Hz, 2H), 2.28 (s, 3H), 1.15 (t, J = 7.2 Hz, 3H). ^{13}C NMR (75 MHz, $CDCl_3$) δ 166.0, 143.0, 135.8, 135.1, 129.7, 128.8, 128.5, 125.2, 121.2, 60.2, 19.9, 14.0.

***E* isomer (minor):** ^{1}H NMR (300 MHz, $CDCl_3$) δ 7.98 (d, J = 15.9 Hz, 1H), 7.56-7.54 (m, 2H), 6.93 – 6.91 (m, 2H), 6.36 (d, J = 15.9 Hz, 1H), 4.27 (q, J = 7.2 Hz, 2H), 2.44 (s, 3H), 1.34 (t, J = 7.2 Hz, 3H). ^{13}C NMR (75 MHz, $CDCl_3$) δ 167.1, 142.3, 137.7, 133.5, 130.8, 130.0, 126.5, 126.4, 119.4, 60.5, 19.9, 14.4.

HRMS (ESI+) for $C_{12}H_{14}O_2$ [M] +: calc. 191.1063; found: 191.1072.

3. Mechanistic studies

3.1. UV – Visible studies

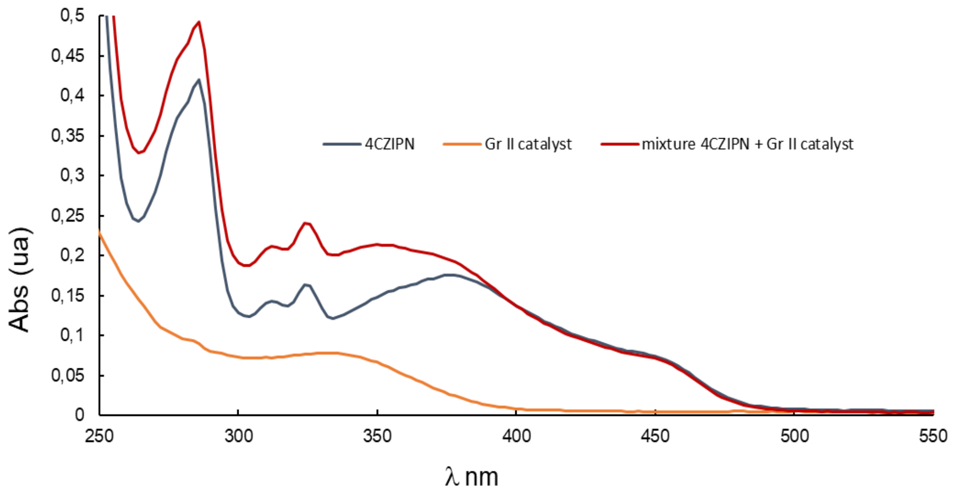

Figure S2 Spectra of 4CzIPN (10^{-5} M, blue), catalyst Grubbs 2nd generation (10^{-5} M, red) in CH_2Cl_2 and solution mixture of 4CzIPN (10^{-5} M) + catalyst Grubbs 2nd generation (10^{-5} M) in CH_2Cl_2 (Orange).

3.2. Fluorescence studies and Stern-Volmer analysis

Table S1. Fluorescence quenching of 4CzIPN by catalyst Grubbs 2nd generation in CH_2Cl_2

[Grubbs 2nd catalyst] mol L^{-1}	Fluorescence intensity (I)	I_0/I
0	577	1
1×10^{-5}	502	1.14
2×10^{-5}	446	1.29
3×10^{-5}	373	1.54
4×10^{-5}	354	1.62
5×10^{-5}	308	1.87
6×10^{-5}	272	2.12
1×10^{-4}	183	3.15
2×10^{-4}	103	5.60

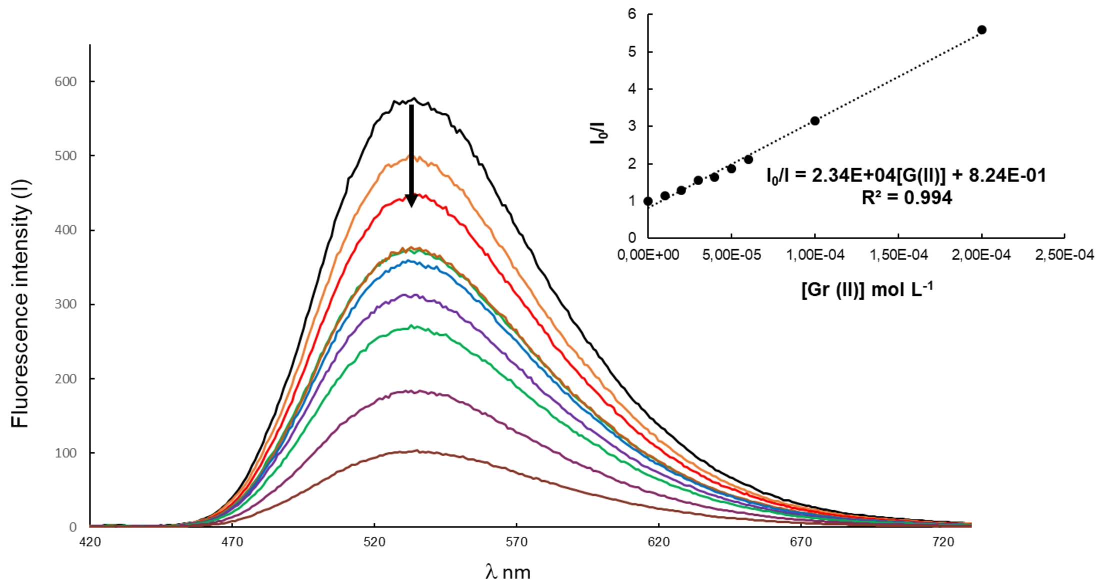

Figure S3. Fluorescence quenching of 4CzIPN (3 x 10^{-6} mol L^{-1}) by catalyst Grubbs 2nd generation in CH_2Cl_2. The arrow in the figure indicates the direction increasing catalyst Grubbs 2nd generation concentration as well as the peak where fluorescence intensity values were taken for Stern-Volmer analysis (insert).

Table S2. Fluorescence quenching of 4CzIPN by styrene in CH_2Cl_2

[Styrene] mol L^{-1}	Fluorescence intensity (I)	I_0/I
0	577	1
5 x 10^{-5}	558	1.14
1 x 10^{-4}	470	1.29
1.5 x 10^{-4}	467	1.54
2 x 10^{-4}	460	1.62
3 x 10^{-4}	450	1.87
5 x 10^{-4}	116	2.12
1 x 10^{-3}	100	3.15

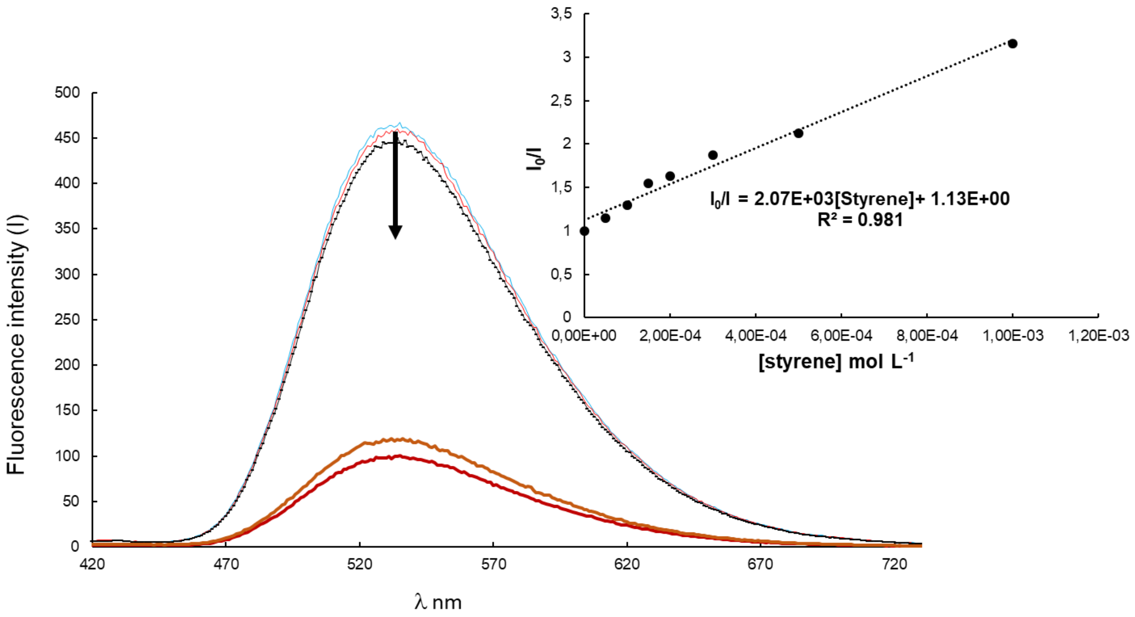

Figure S4. Fluorescence quenching of 4CzIPN (3 x 10^{-6} mol L^{-1}) by styrene in CH_2Cl_2. The arrow in the figure indicates the direction increasing styrene concentration as well as the peak where fluorescence intensity values were taken for Stern-Volmer analysis (insert)

Table S3. Fluorescence quenching of 4CzIPN by *cis*-stilbene in CH_2Cl_2

[*cis*-stilbene] mol L^{-1}	Fluorescence intensity (I)	I_0/I
0	953	1
2 x 10^{-4}	883	1.07
4 x 10^{-4}	805	1.18
6 x 10^{-4}	771	1.23
1 x 10^{-3}	685	1.39
1.3 x 10^{-3}	623	1.52
2 x 10^{-3}	558	1.70

3 x 10^{-3}	468	2.03
4 x 10^{-3}	389	2.44

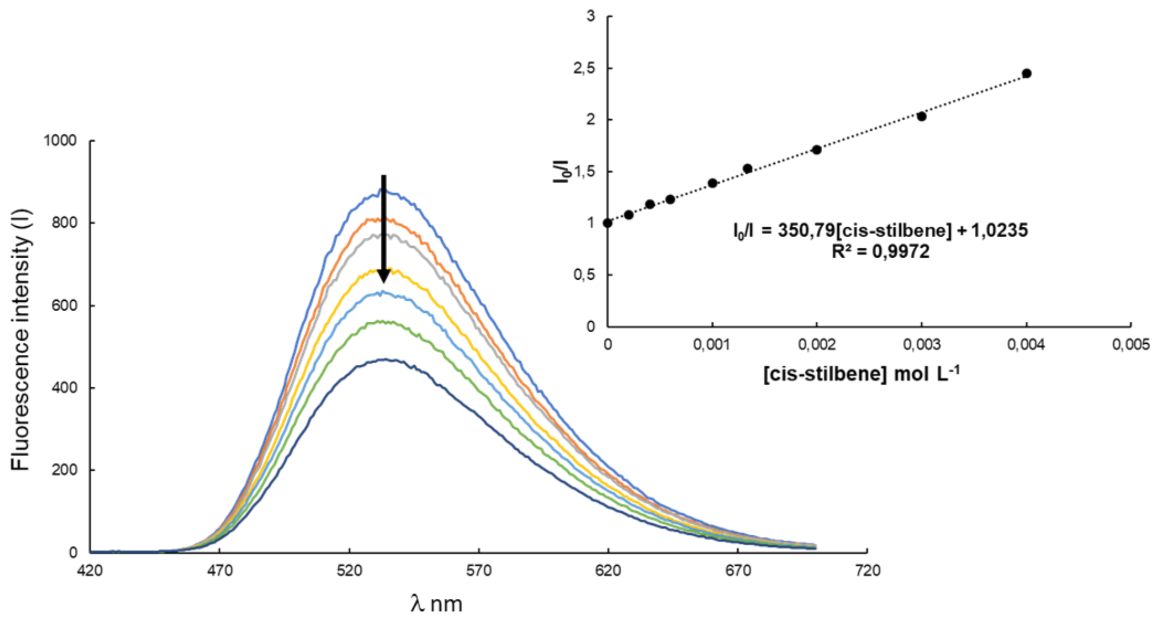

Figure S5. Fluorescence quenching of 4CzIPN (3 x 10^{-6} mol L^{-1}) by *cis*-stilbene in CH_2Cl_2. The arrow in the figure indicates the direction increasing cis-stilbene concentration as well as the peak where fluorescence intensity values were taken for Stern-Volmer analysis (insert).

Table S4. Fluorescence quenching of 4CzIPN by *trans*-stilbene in CH_2Cl_2

[*trans*-stilbene] mol L^{-1}	Fluorescence intensity (I)	I_0/I
0	953	1
2 x 10^{-4}	650	1.46
4 x 10^{-4}	635	1.50
6 x 10^{-4}	476	1.99
1 x 10^{-3}	399	2.38
1.3 x.10^{-3}	367	2.59
2 x 10^{-3}	33	2.80

3 x 10^{-3}	271	3.50
4 x 10^{-3}	239	3.97

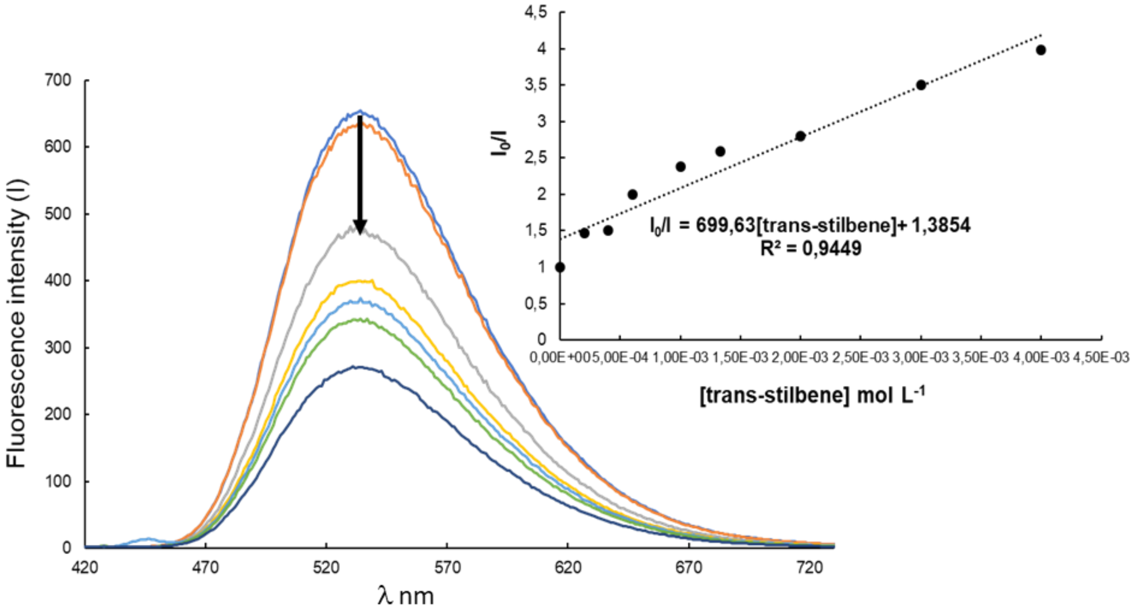

Figure S6. Fluorescence quenching of 4CzIPN (3 x 10^{-6} mol L^{-1}) by *trans*-stilbene in CH_2Cl_2. The arrow in the figure indicates the direction increasing trans-stilbene concentration as well as the peak where fluorescence intensity values were taken for Stern-Volmer analysis (insert)

3.3. Laser flash photolysis

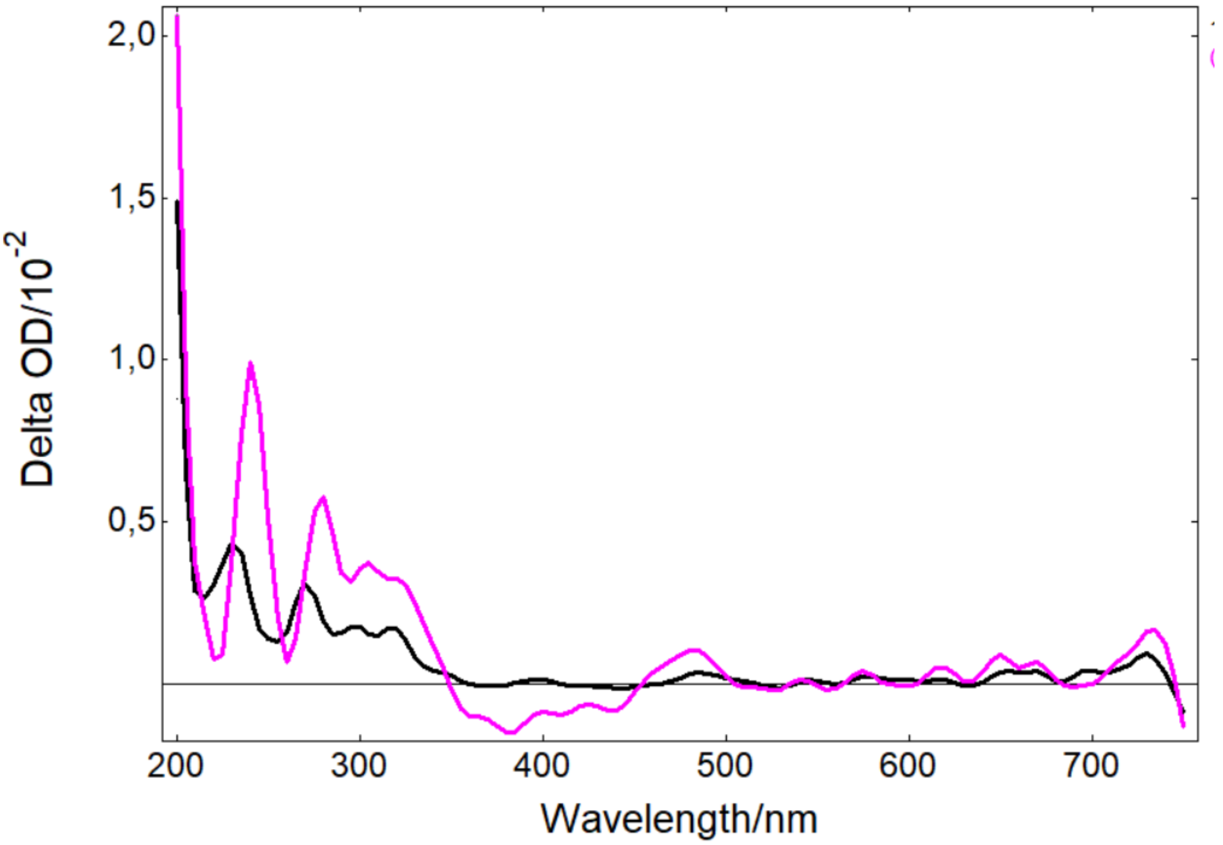

Figure S7. Transient UV-vis spectra following irradiation of Grubbs second-generation catalyst (**2**) at 355 nm (black) and a mixture of **2** with **PCf** (pink) at the same wavelength in CH_2Cl_2

4. Computational details

All the calculations were performed at the density functional theory (DFT) level using the Orca 4.2.1 program package.[4,5]Geometry optimizations (minima and MECPs) were performed in vacuum without symmetry using the B3LYP functional,[6,7]a relativistic small core pseudopotential (SD28-Def2-ECP) and its associated basis set for Ru,[8,9] the def2-TZVP(-f) basis set for all other atoms,[8] and the empirical D3-BJ dispersion correction.[10,11] The restricted Kohn–Sham formalism was used for ground states, while its unrestricted analogue was used for triplet states and oxidized states. SCF convergence was achieved using the DIIS algorithm followed by a semi-quadratic SOSCF converger. The RIJCOSX approximation was applied for all calculations using the def2/J auxiliary basis sets.[12] Frequency calculations were run at the same level of theory. No imaginary frequencies were obtained, except in rare cases in which there was one imaginary frequency originating from methyl rotation (ca –80 cm^{-1}).

In the TD-DFT calculations, 50 singlets and 20 triplets were computed in the framework of the Tamm-Dancoff approximation. Molecular orbitals were viewed using Gabedit.[13]

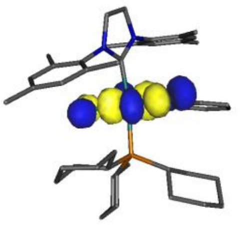 HOMO	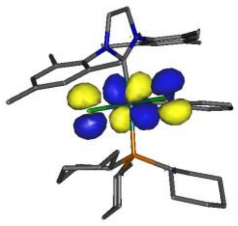 HOMO–1	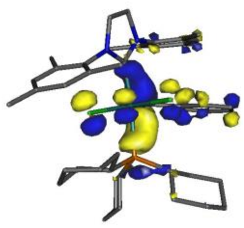 HOMO–2	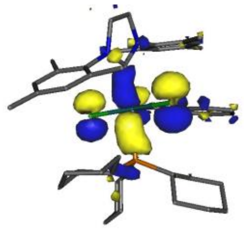 HOMO–3
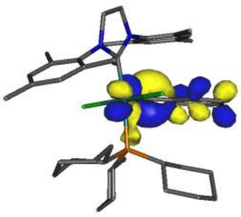 LUMO	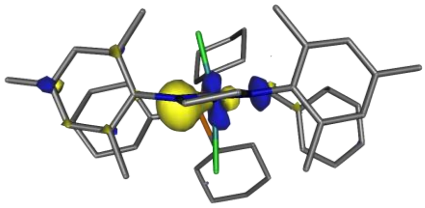 LUMO+1 (top view)	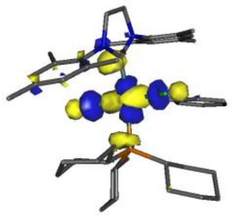 LUMO+7	

Figure S8 Selected Kohn-Sham orbitals for complex 2 optimized in the ground state.

Table S5. Franck-Condon singlet excited states for complex 2 (E ≤ 4 eV).

The two transitions in bold are the ones giving rise to the absorption spectral profile, with apparent absorption bands at 494 nm and 348 nm.

state	λ (nm)	E (eV)	oscillator strength	major excitation
1	696	1.78	0.000295786	HO --> LU
2	602	2.06	0.000101093	HO-1 --> LU
**3**	**494**	**2.51**	**0.004295395**	**HO --> LU+1**
4	419	2.96	0.000125864	HO-1 --> LU+1
5	380	3.26	0.001454459	HO-1 --> LU+7
6	364	3.41	0.006151611	HO --> LU+7
**7**	**348**	**3.56**	**0.088089164**	**HO-2 --> LU**
8	333	3.72	0.002036370	HO --> LU+2
9	325	3.81	0.037641954	HO-4 --> LU
10	324	3.83	0.025993159	HO-5 --> LU<br>HO-3 --> LU
11	318	3.90	0.019225623	HO --> LU+3<br>HO-5 --> LU
12	317	3.91	0.017883859	HO --> LU+3<br>HO-5 --> LU
13	313	3.96	0.026418174	HO --> LU+5<br>HO-1 --> LU+2
14	310	4.00	0.000039857	HO --> LU+4

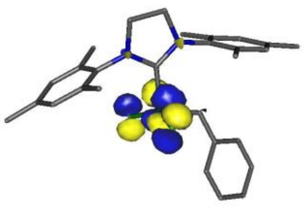 HOMO	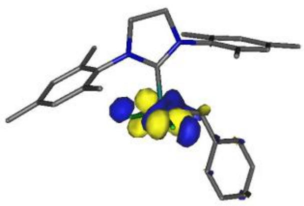 HOMO–1	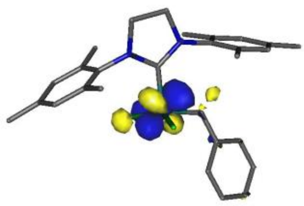 HOMO–2
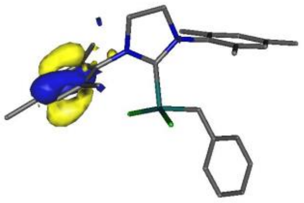 HOMO–3		
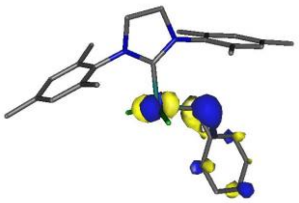 LUMO	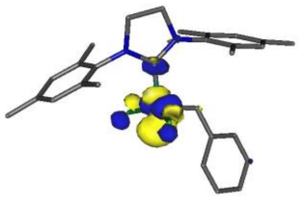 LUMO+1	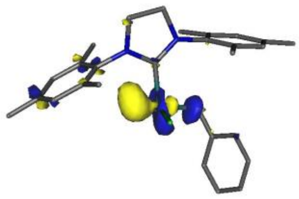 LUMO+2

Figure S9. Selected Kohn-Sham orbitals for complex 9 optimized in the ground state.

Table S6. Franck-Condon singlet excited states for complex 9 (E ≤ 4 eV).

state	λ (nm)	E (eV)	oscillator strength	major excitation
1	566	2.19	0.004581685	HO --> LU
2	516	2.40	0.002759161	HO --> LU+1
3	494	2.51	0.002842105	HO-1, HO-2 --> LU
4	417	2.98	0.004480958	HO-1, HO-2 --> LU+1
5	382	3.24	0.002778584	HO-1, HO-2 --> LU+1
6	370	3.35	0.010016529	HO --> LU+2
7	355	3.49	0.146461633	HO-1, HO-2 --> LU<br>HO-1, HO-2 --> LU+2
8	349	3.55	0.139007164	HO-1, HO-2 --> LU<br>HO-1, HO-2 --> LU+2
9	314	3.95	0.031039946	HO-3 --> LU
10	311	3.99	0.010140449	HO-1, HO-2 --> LU+2

	complex **2**	complex **9**
β-LUMO (hole)	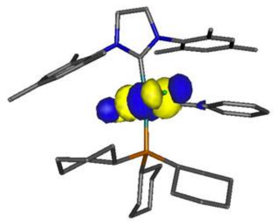	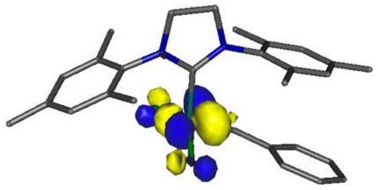
α-HOMO (particle)	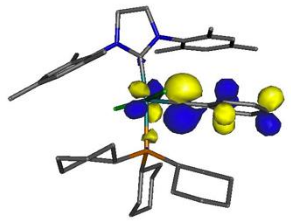	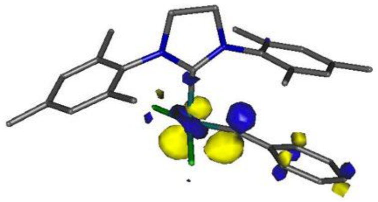
spin density	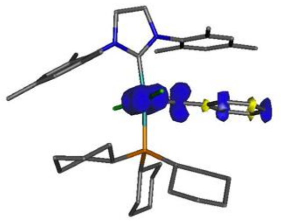	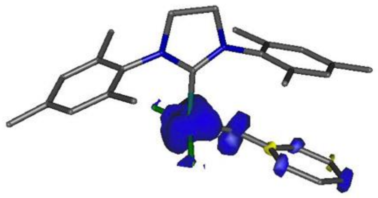

Figure S10. Selected Kohn-Sham orbitals and spin density for complexes 2 and 9 optimized in the lowest triplet state (Ru-ben MLCT character; ben=benzylidene).

β-LUMO (hole)	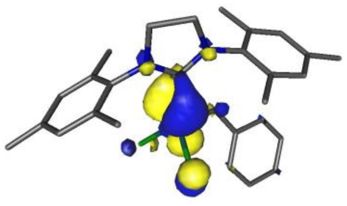	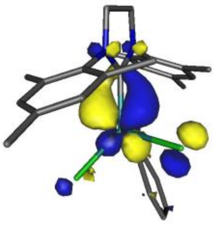 (side view)
α-HOMO (particle)	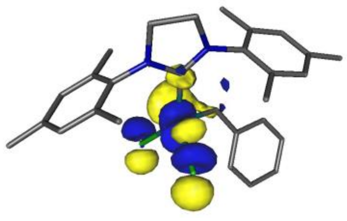	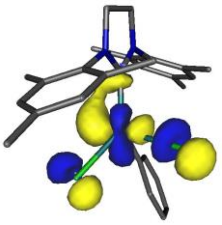 (side view)
spin density	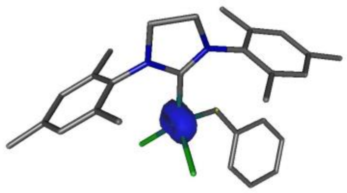	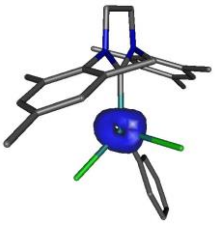 (side view)

Figure S11. Selected Kohn-Sham orbitals and spin density for complex 9 optimized in the ^{3}MC state (Ru-C(car) antibonding character; car=N-heterocyclic carbene).

Table S7. Selected distances (A) and angles (°), and Mulliken spin density.

C(car) refers to N-heterocyclic carbene; C(ben) refers to benzylidene.

Complex	Ru-P	Ru-C(ben)	Ru-C(car)	Ru-Cl	Ru-Cl	C-C(ben)	C(car)-Ru-C(ben)	spin/Ru
**2_GS**	2.419	1.836	2.082	2.435	2.432	1.457	100.95	-
***2-^{3}MLCT**	2.442	1.961	2.101	2.343	2.363	1.405	98.56	1.01
**2_^{3}MECP(MLCT)**	2.436	2.072	2.125	2.281	2.310	1.378	95.81	0.98
**9_GS**	-	1.825	1.937	2.331	2.323	1.449	102.68	-
***9_^{3}MLCT**	-	1.892	1.995	2.313	2.326	1.430	108.38	1.19
**9_^{3}MECP(MLCT)**	-	1.896	2.003	2.307	2.321	1.429	110.60	1.20
***9_MC(car)**	-	1.844	2.096	2.328	2.370	1.447	94.55	1.69
**9_^{3}MECP(MC)**	-	1.848	2.080	2.333	2.355	1.447	91.95	1.68

Table S8. Computed energies (atomic units).

Species	state / point	charge, multiplicity	Electronic energy	Gibbs free energy
**complex 2**	ground state	0, 1	-3257,92375079253	-3256,97963545
	^{3}MLCT state	0, 3	-3257,88633647512	-3256,94670799<br>ΔG^a = 20.7 kcal/mol
	MECP	0, 3	-3257,87799501900	-
**complex 9**	ground state	0, 1	-2210,86689395221	-2210,39735152
	^{3}MLCT state	0, 3	-2210,84187471702	-2210,37413766<br>ΔG = 14.6 kcal/mol
	MECP(MLCT)	0, 3	-2210,84164811032	-
	^{3}MC state	0, 3	-2210,83285232734	-2210,36544518<br>ΔG = 20.0 kcal/mol
	MECP(MC)	0, 3	-2210,83239368771	-
**PCf**	ground state	0, 1	-2481,36413845560	-2480,71245138
	triplet state	0, 3	-2481,2758127722	-2480,63065102<br>ΔG = 51.3 kcal/mol

PCy_3	ground state	0, 1	-1046,98000842924	-1046,54118648
E-stilbene	ground state	0, 1	-540,594693269745	-540,41838917
	triplet state	0, 3	-540,517412171159	-540,34537016<br>ΔG = 45.8 kcal/mol
Z-stilbene	ground state	0, 1	-540,589463145081	-540,41164067
	triplet state	0, 3	-540,523061754208	-540,35076745<br>ΔG = 38.2 kcal/mol
Styrene	ground state	0, 1	-309,580779324063	-309,47863231
	triplet state	0, 3	-309,493203279912	-309,39713709<br>ΔG = 51.1 kcal/mol

[a] Gibbs free energy difference with ground state.

5. Copies of ^{1}H-, ^{13}C- and ^{19}F-NMR Spectra

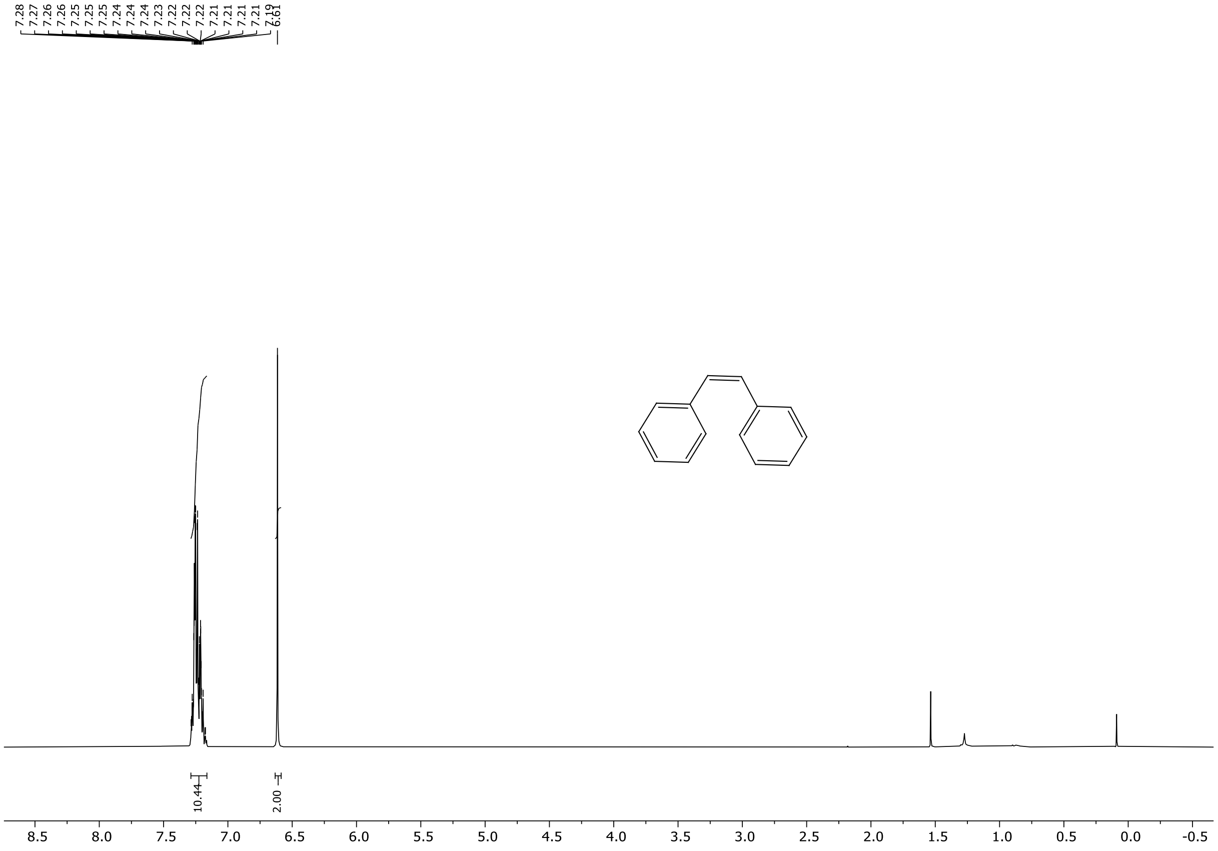

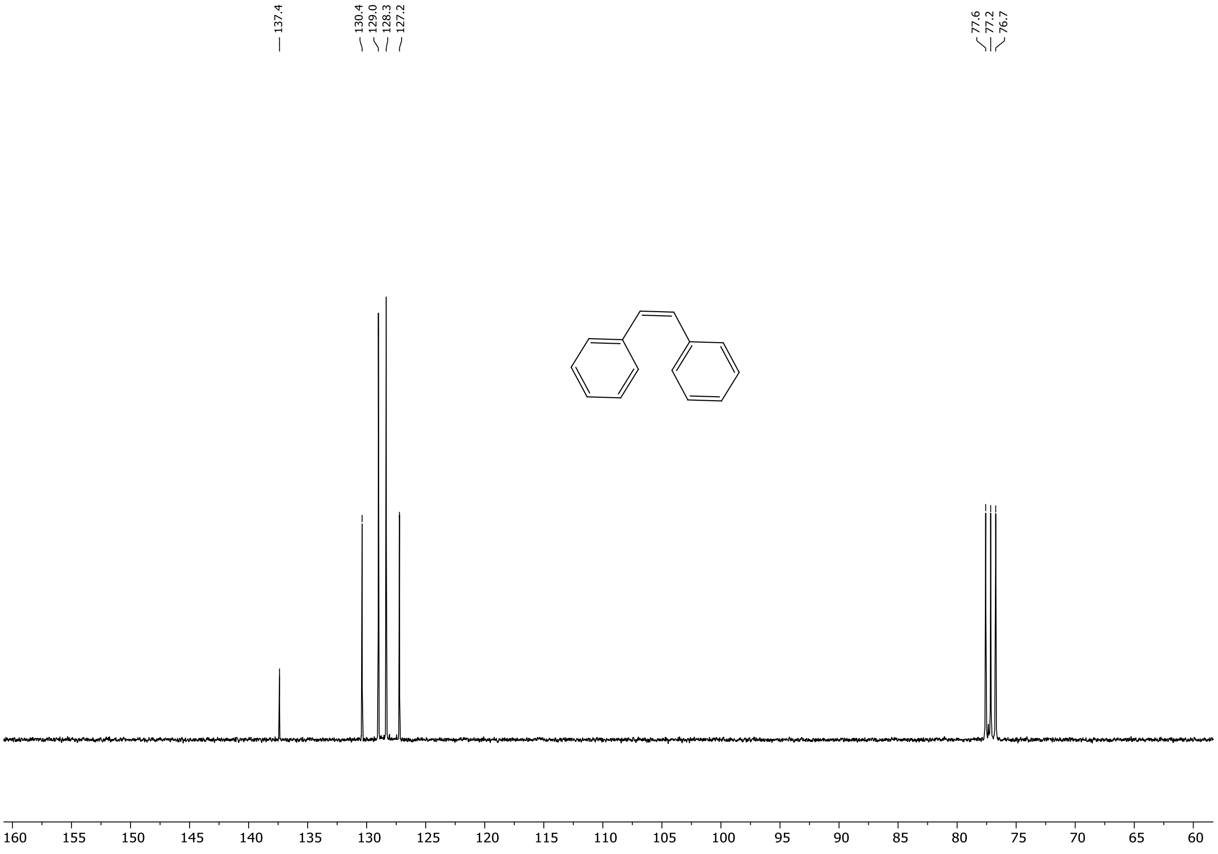

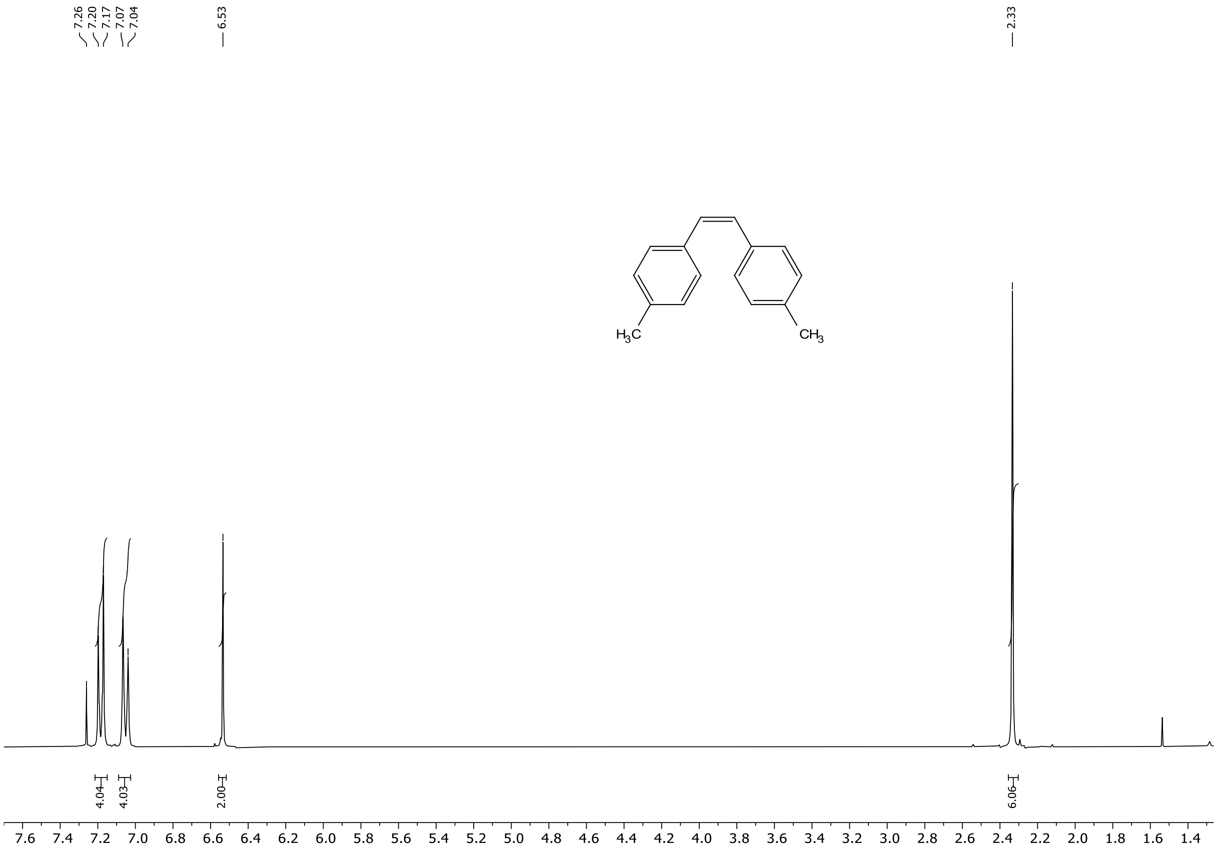
7.26
7.20
7.17
7.07
7.04
6.53
2.33
H_3C
CH_3
4.04
4.03
2.00
6.06
7.6 7.4 7.2 7.0 6.8 6.6 6.4 6.2 6.0 5.8 5.6 5.4 5.2 5.0 4.8 4.6 4.4 4.2 4.0 3.8 3.6 3.4 3.2 3.0 2.8 2.6 2.4 2.2 2.0 1.8 1.6 1.4

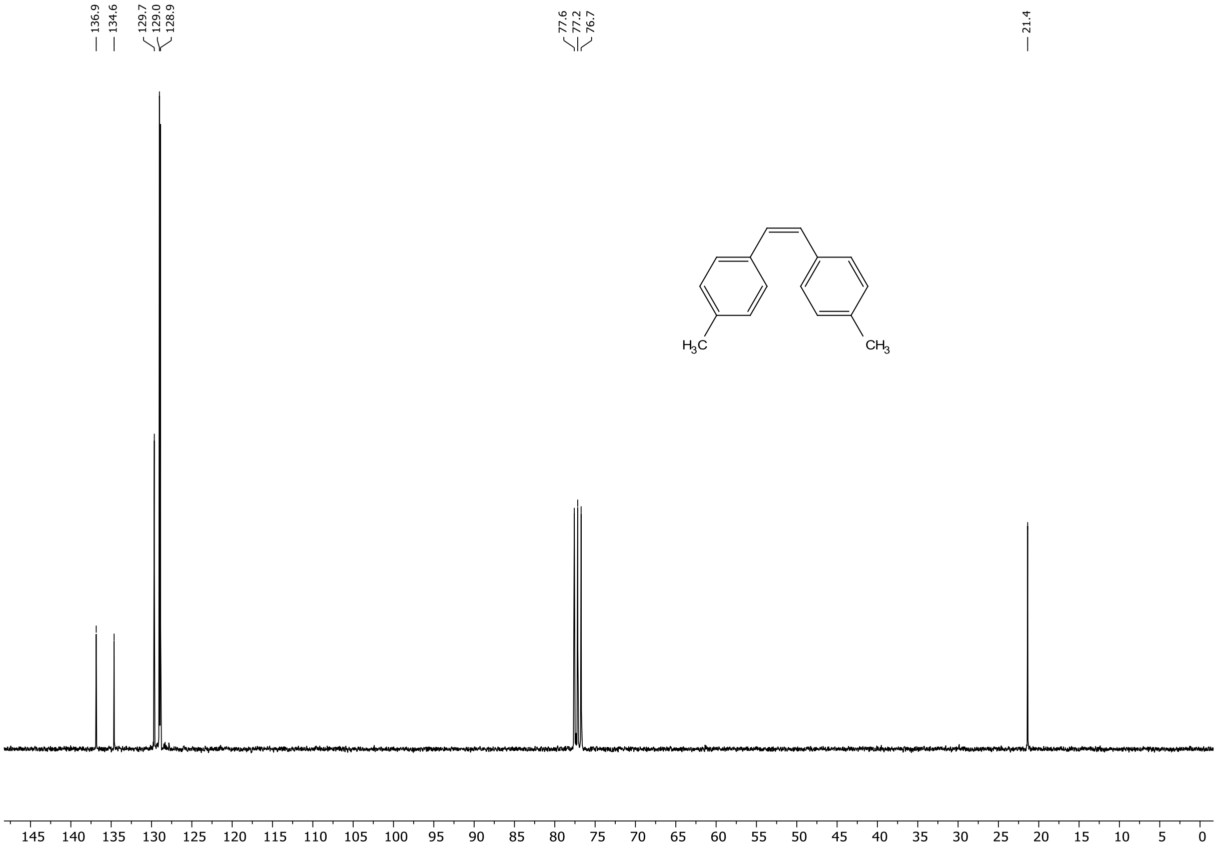
136.9
134.6
129.7
129.0
128.9
77.6
77.2
76.7
21.4
H_3C
CH_3
145 140 135 130 125 120 115 110 105 100 95 90 85 80 75 70 65 60 55 50 45 40 35 30 25 20 15 10 5 0

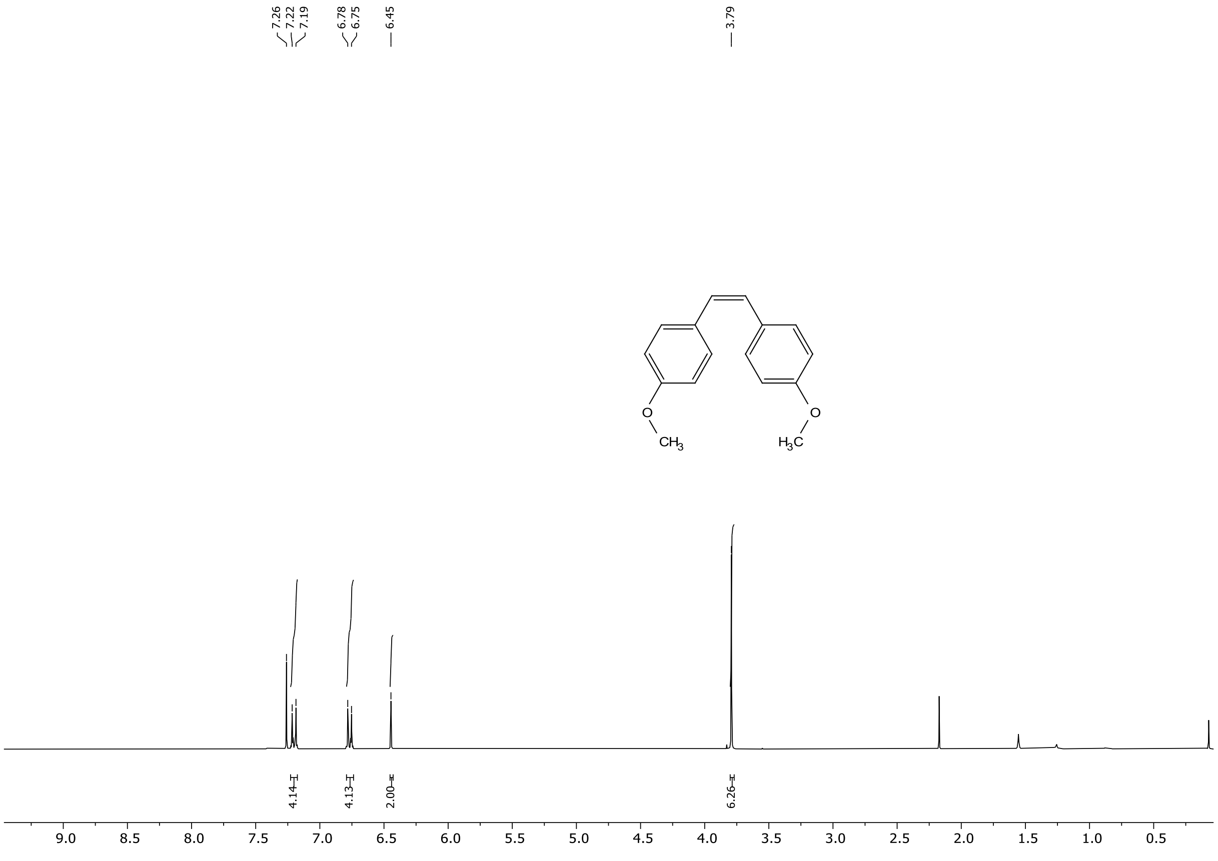
7.26
7.22
7.19
6.78
6.75
6.45
3.79
CH3
H3C
4.14
4.13
2.00
6.26
9.0 8.5 8.0 7.5 7.0 6.5 6.0 5.5 5.0 4.5 4.0 3.5 3.0 2.5 2.0 1.5 1.0 0.5

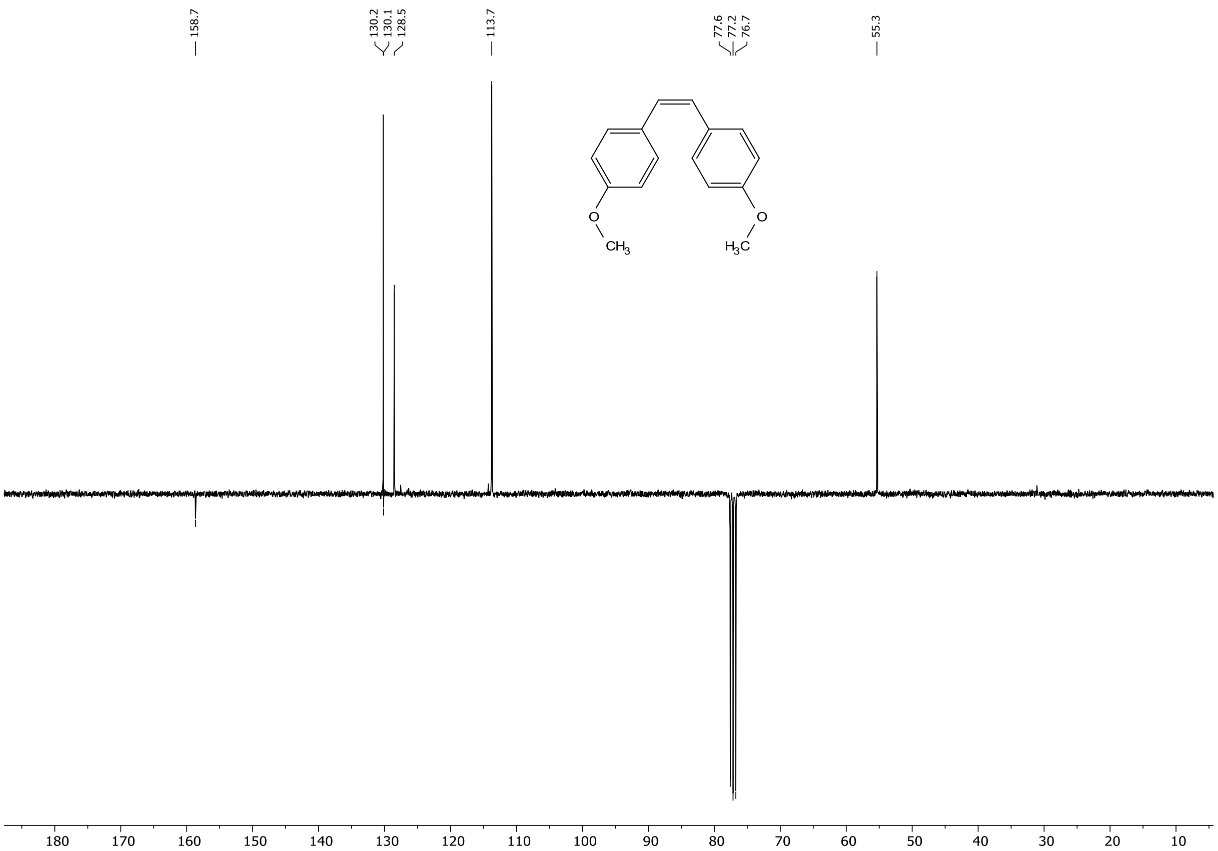
158.7
130.2
130.1
128.5
113.7
77.6
77.2
76.7
55.3
CH3
H3C
180 170 160 150 140 130 120 110 100 90 80 70 60 50 40 30 20 10

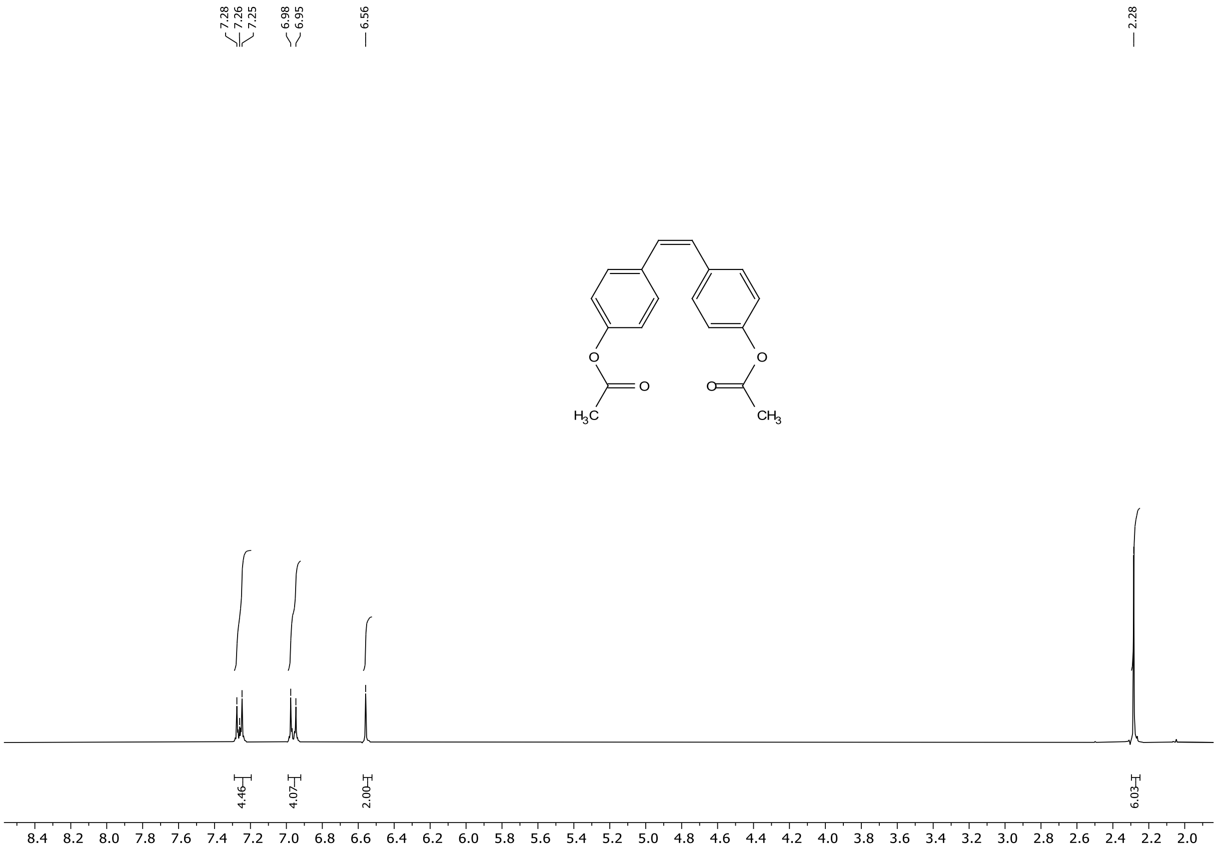
7.28
7.26
7.25
6.98
6.95
6.56
2.28
O
O
O
O
H3C
CH3
4.46
4.07
2.00
6.03
8.4 8.2 8.0 7.8 7.6 7.4 7.2 7.0 6.8 6.6 6.4 6.2 6.0 5.8 5.6 5.4 5.2 5.0 4.8 4.6 4.4 4.2 4.0 3.8 3.6 3.4 3.2 3.0 2.8 2.6 2.4 2.2 2.0

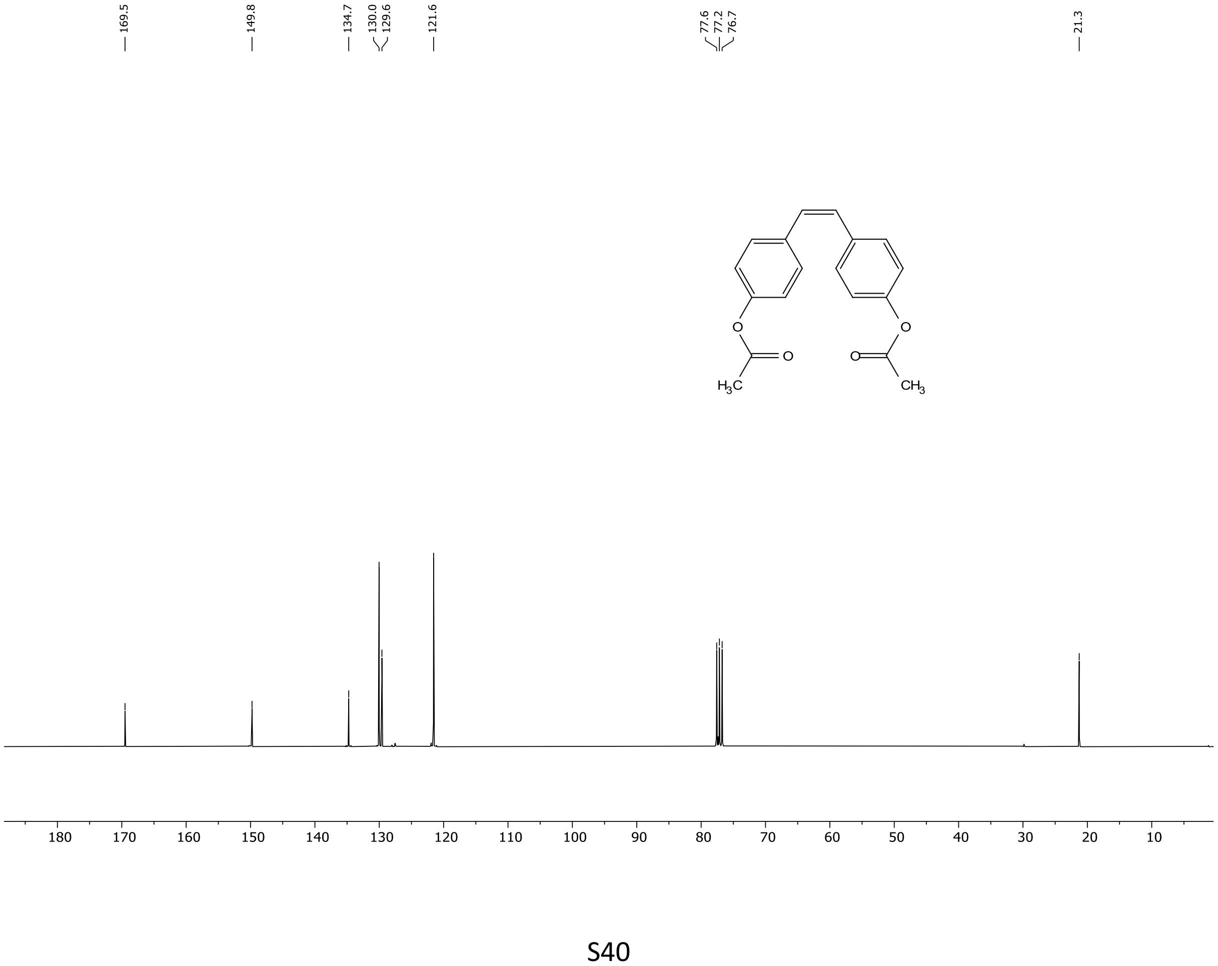
169.5
149.8
134.7
130.0
129.6
121.6
77.6
77.2
76.7
21.3
O
O
O
O
H3C
CH3
180 170 160 150 140 130 120 110 100 90 80 70 60 50 40 30 20 10

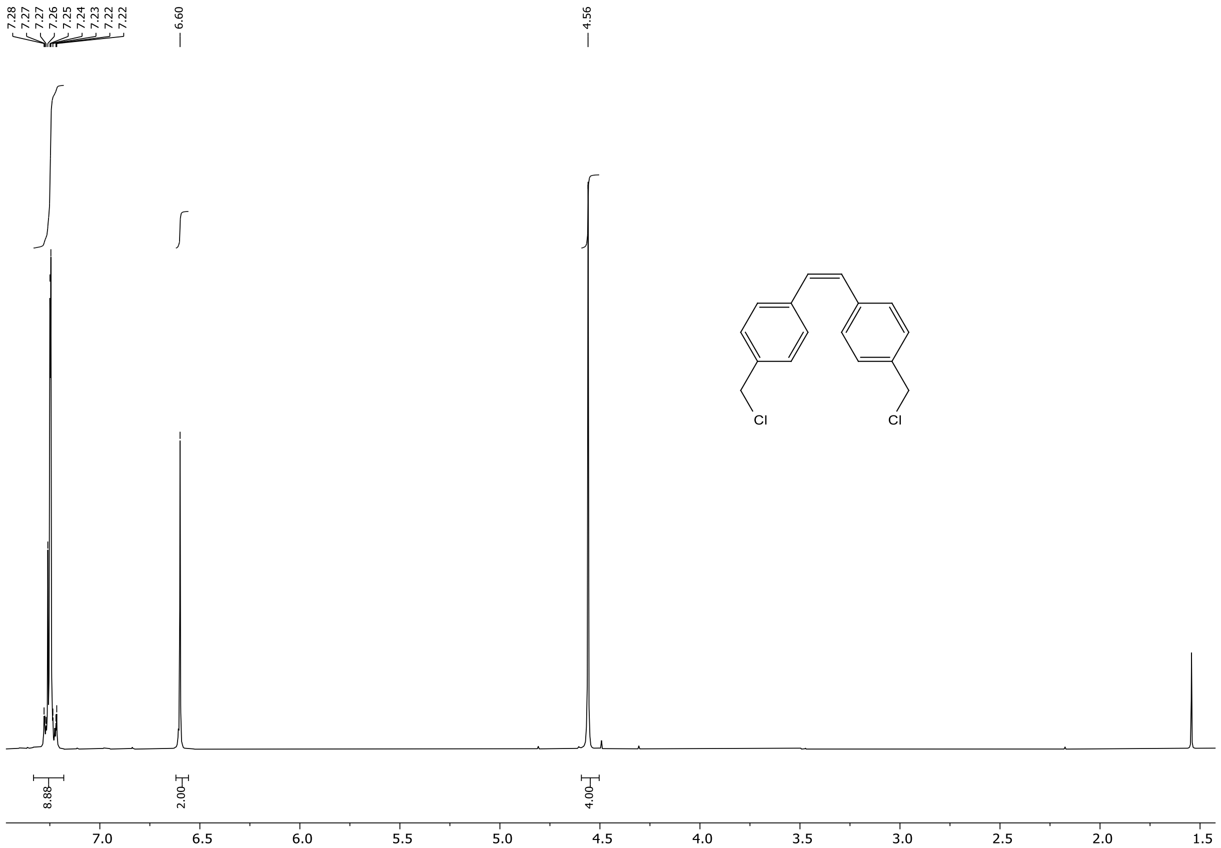
7.28
7.27
7.27
7.26
7.25
7.24
7.23
7.22
7.22
6.60
4.56
Cl
Cl
8.88
2.00
4.00
7.0 6.5 6.0 5.5 5.0 4.5 4.0 3.5 3.0 2.5 2.0 1.5

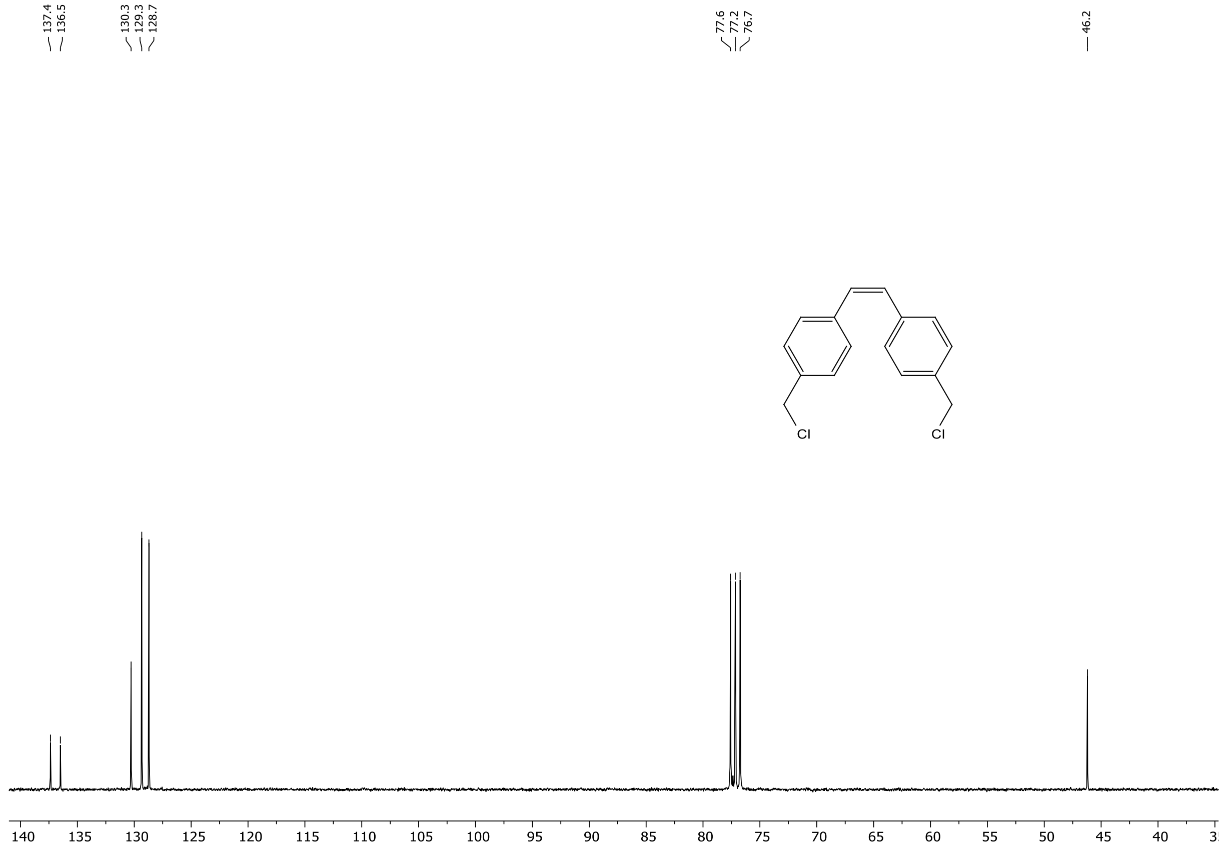
137.4
136.5
130.3
129.3
128.7
77.6
77.2
76.7
46.2
Cl
Cl
140 135 130 125 120 115 110 105 100 95 90 85 80 75 70 65 60 55 50 45 40

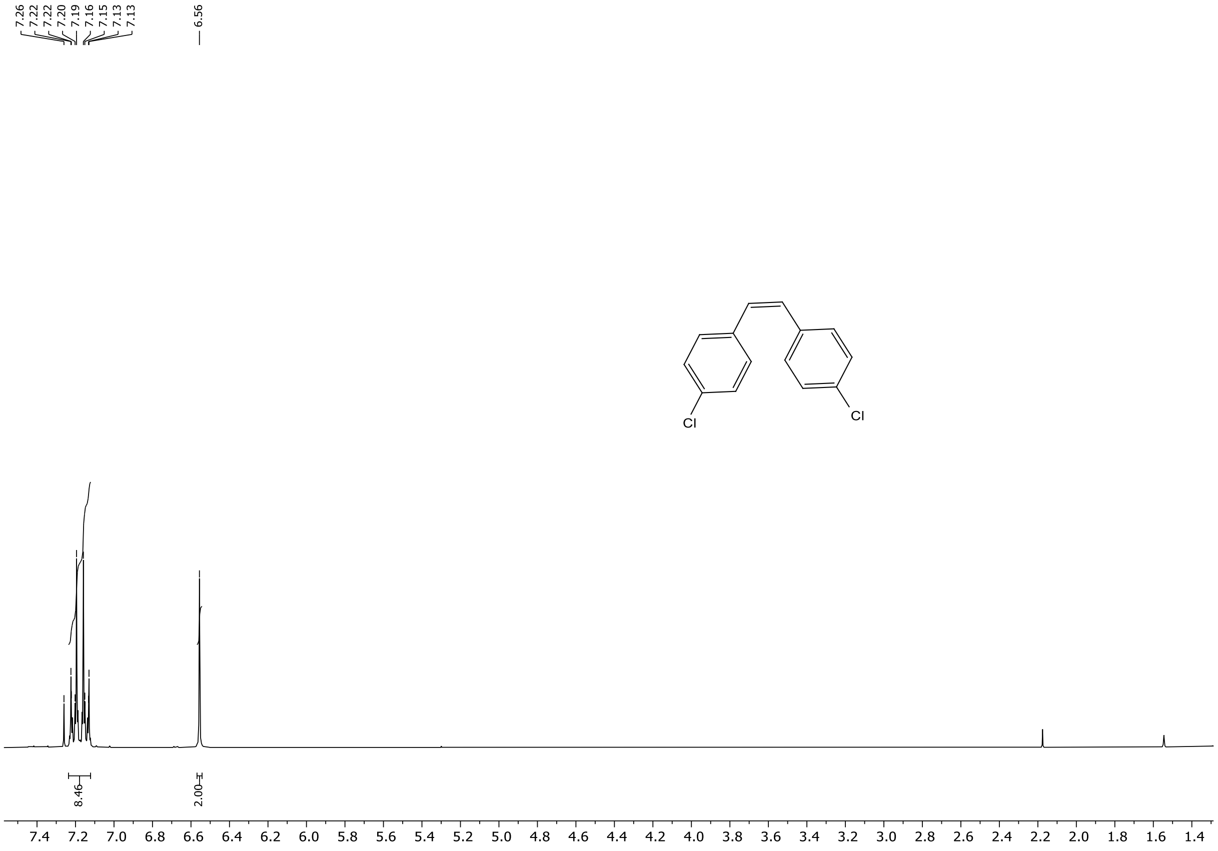

7.26
7.22
7.22
7.20
7.19
7.16
7.15
7.13
7.13
6.56
Cl
Cl
8.46
2.00
7.4 7.2 7.0 6.8 6.6 6.4 6.2 6.0 5.8 5.6 5.4 5.2 5.0 4.8 4.6 4.4 4.2 4.0 3.8 3.6 3.4 3.2 3.0 2.8 2.6 2.4 2.2 2.0 1.8 1.6 1.4

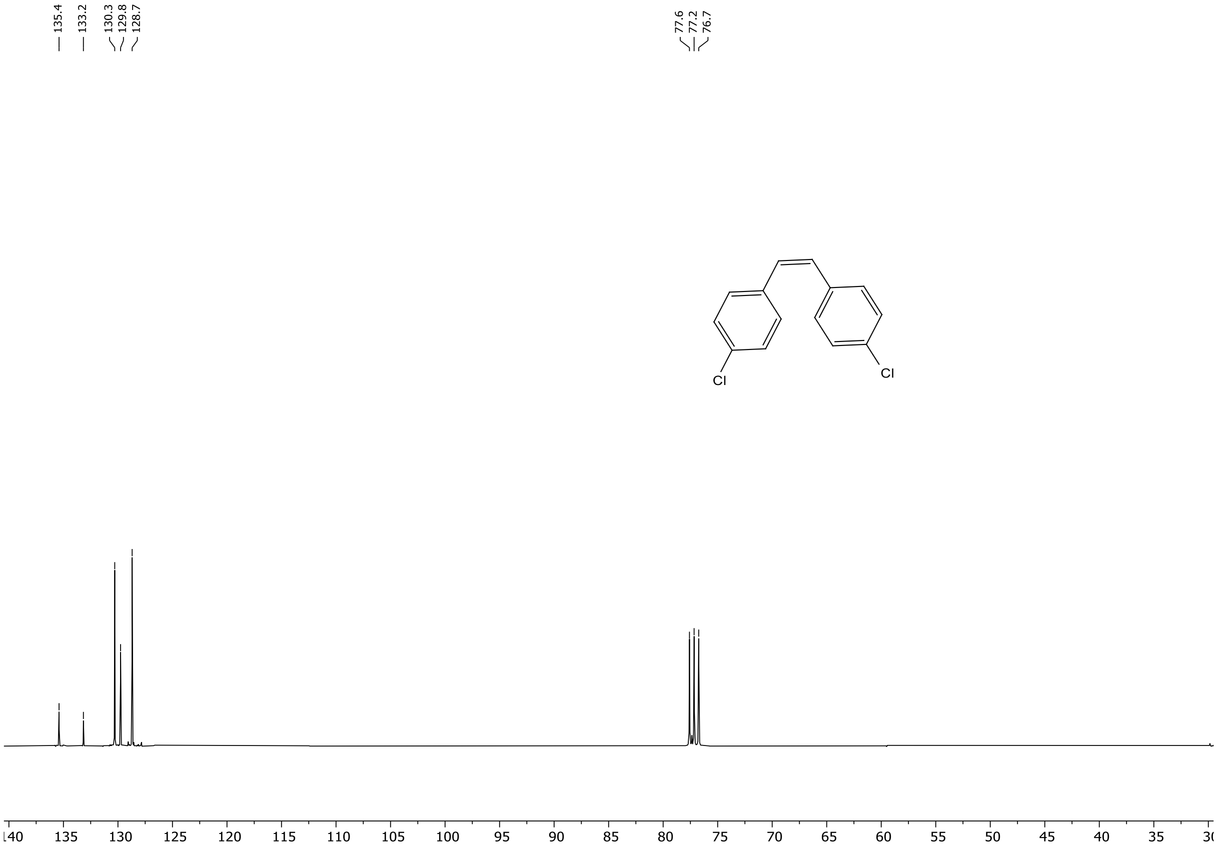

135.4
133.2
130.3
129.8
128.7
77.6
77.2
76.7
Cl
Cl
135 130 125 120 115 110 105 100 95 90 85 80 75 70 65 60 55 50 45 40 35

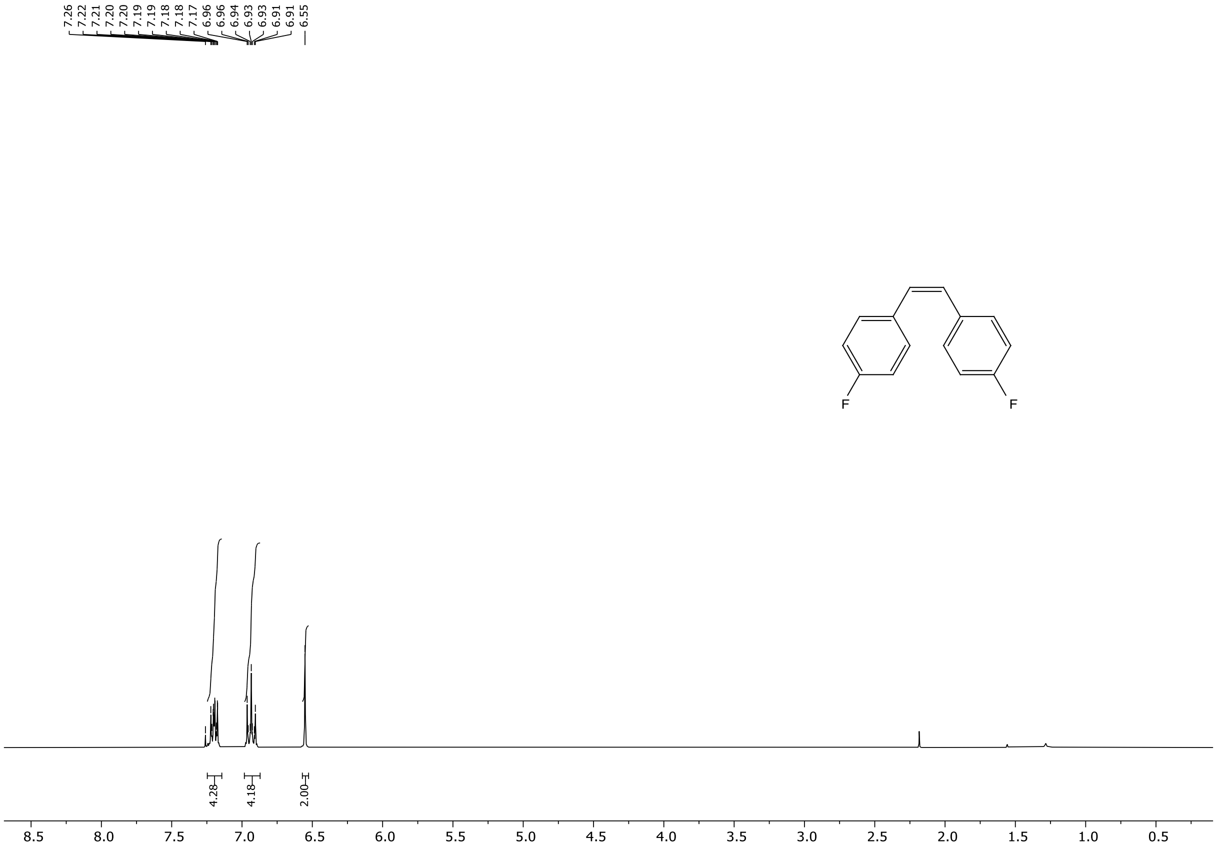
7.26
7.22
7.21
7.20
7.20
7.19
7.19
7.18
7.18
7.17
6.96
6.96
6.94
6.93
6.93
6.91
6.91
6.55
F
F
4.28
4.18
2.00
8.5 8.0 7.5 7.0 6.5 6.0 5.5 5.0 4.5 4.0 3.5 3.0 2.5 2.0 1.5 1.0 0.5

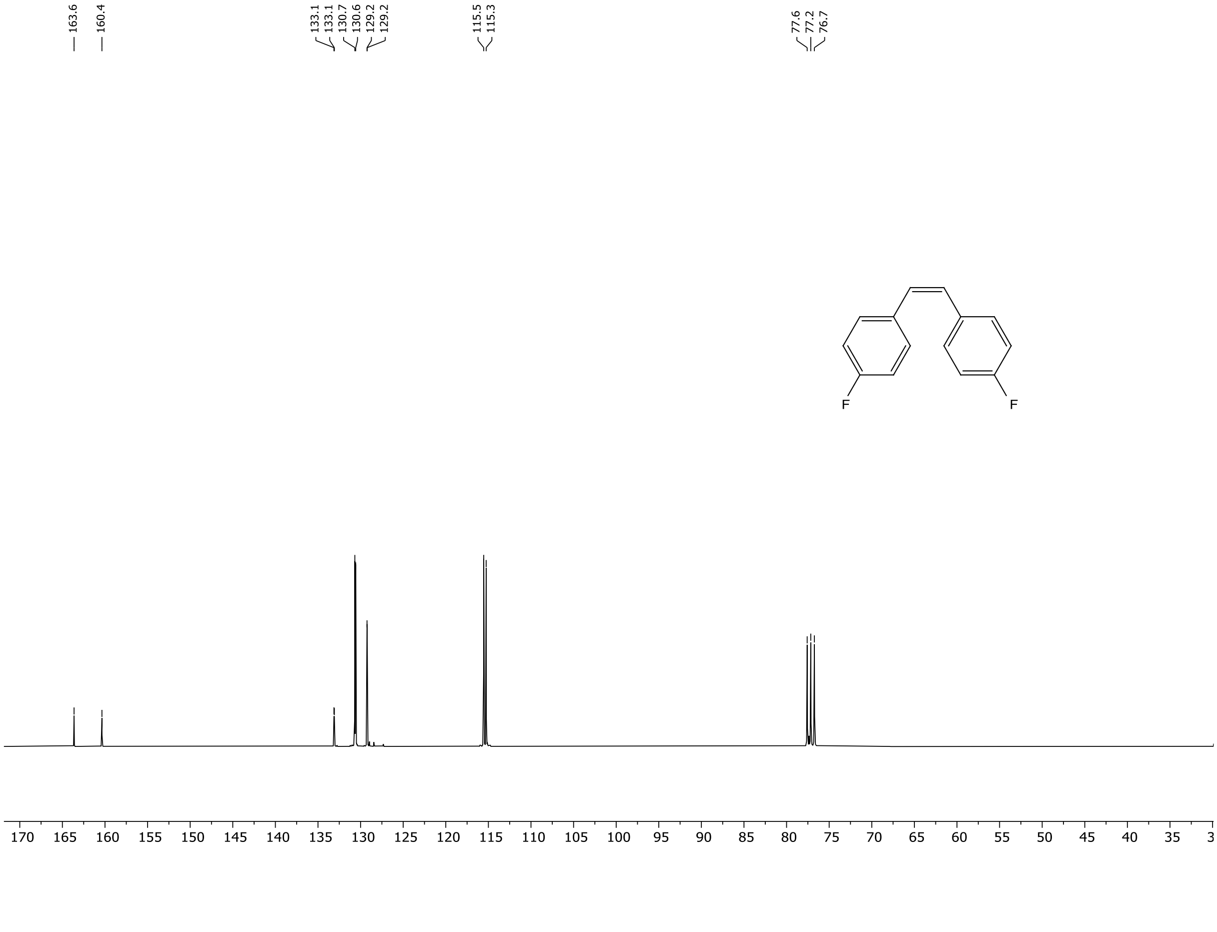
163.6
160.4
133.1
133.1
130.7
130.6
129.2
129.2
115.5
115.3
77.6
77.2
76.7
F
F
170 165 160 155 150 145 140 135 130 125 120 115 110 105 100 95 90 85 80 75 70 65 60 55 50 45 40 35 3

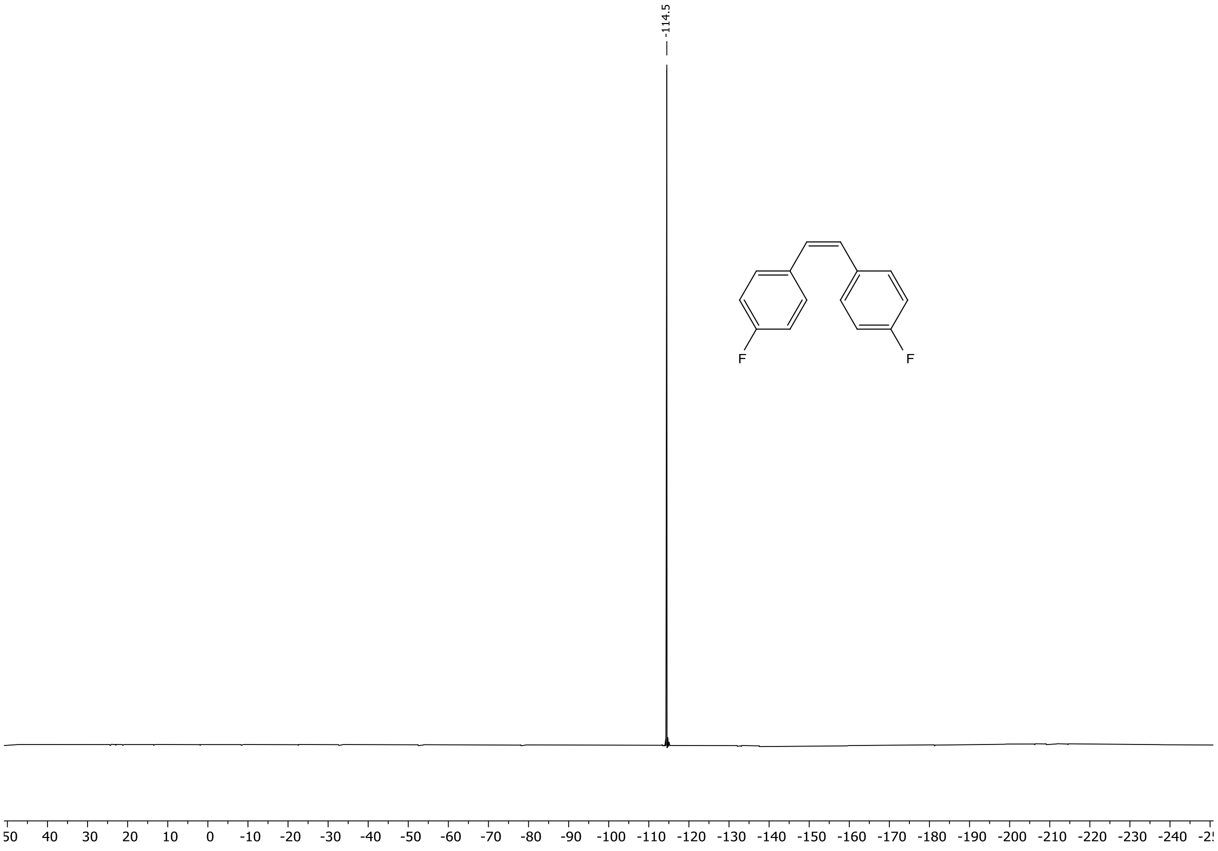
-114.5
F
F
50 40 30 20 10 0 -10 -20 -30 -40 -50 -60 -70 -80 -90 -100 -110 -120 -130 -140 -150 -160 -170 -180 -190 -200 -210 -220 -230 -240

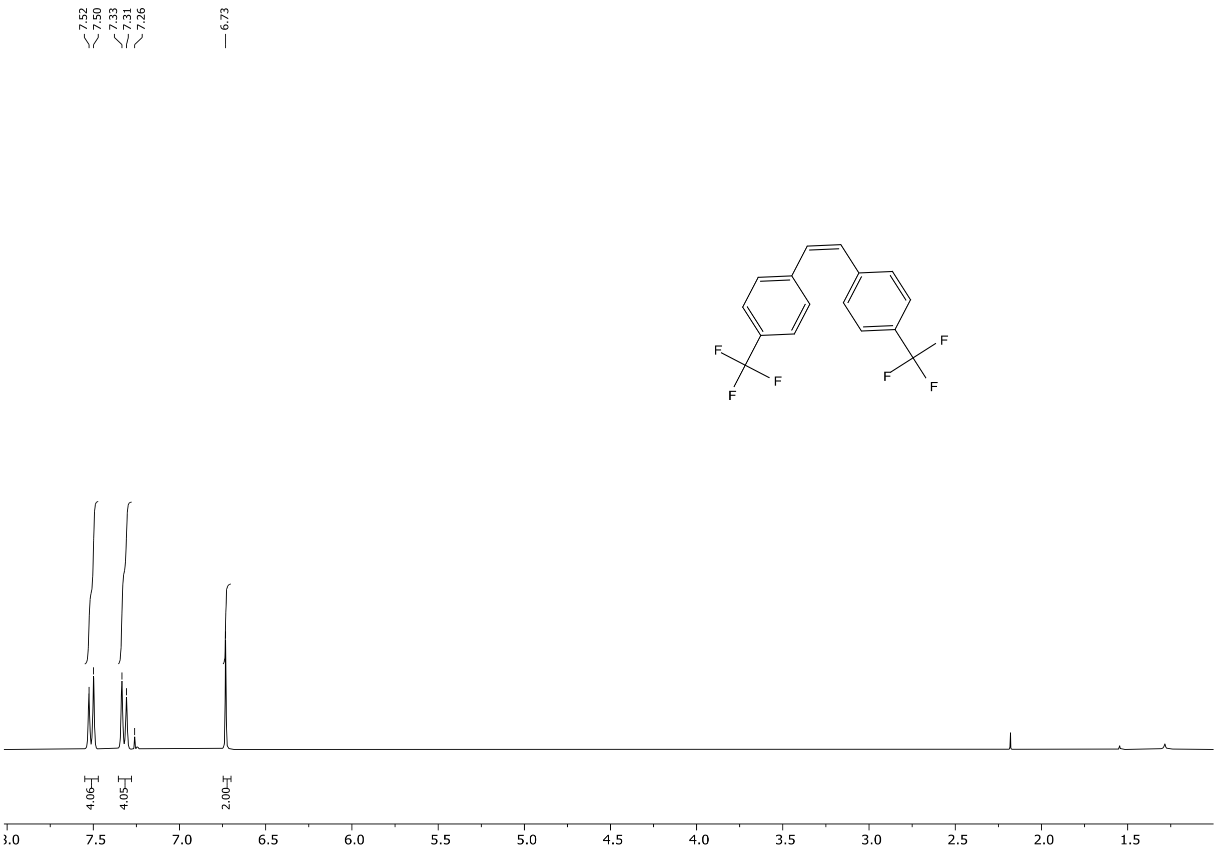
7.52
7.50
7.33
7.31
7.26
6.73
F
F
F
F
F
F
4.06
4.05
2.00
7.5 7.0 6.5 6.0 5.5 5.0 4.5 4.0 3.5 3.0 2.5 2.0 1.5

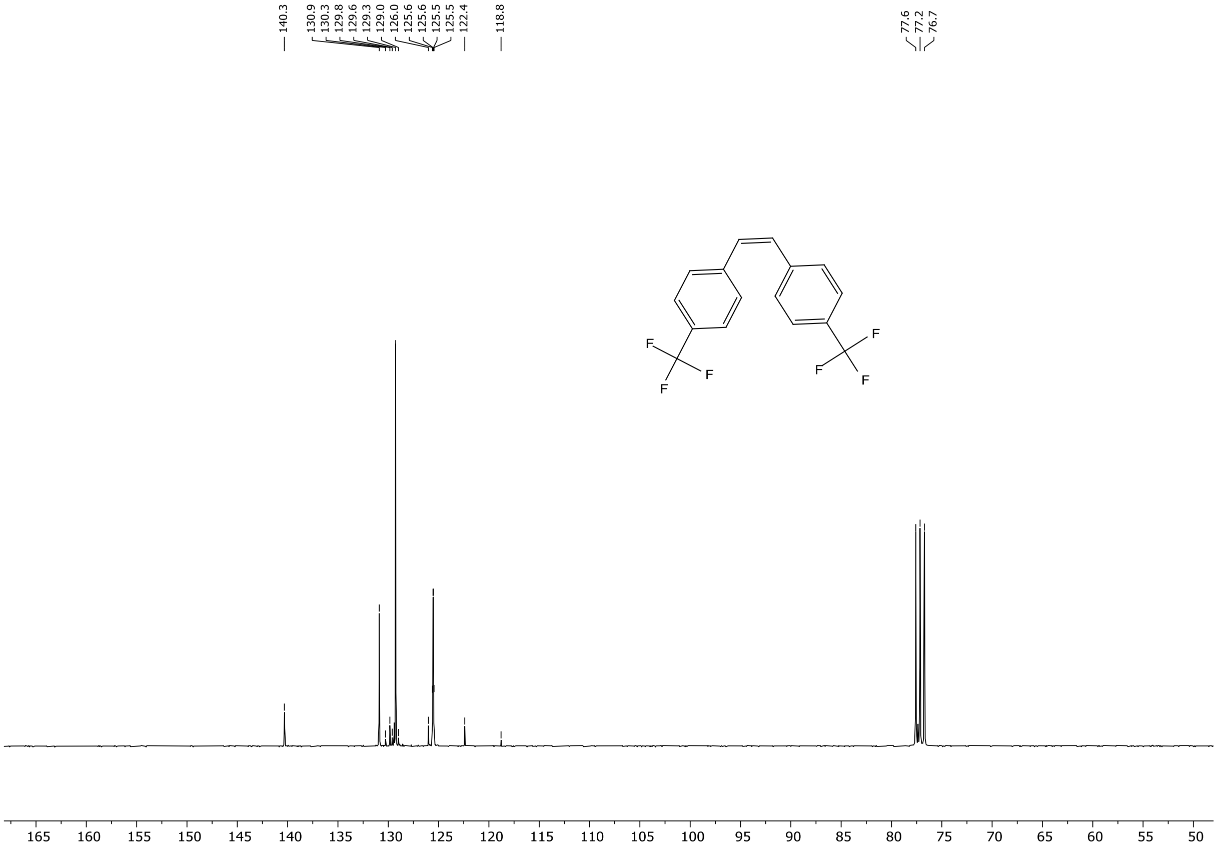
140.3
130.9
130.3
129.8
129.6
129.3
129.0
126.0
125.6
125.6
125.5
125.5
122.4
118.8
77.6
77.2
76.7
F F F F F F
165 160 155 150 145 140 135 130 125 120 115 110 105 100 95 90 85 80 75 70 65 60 55 50

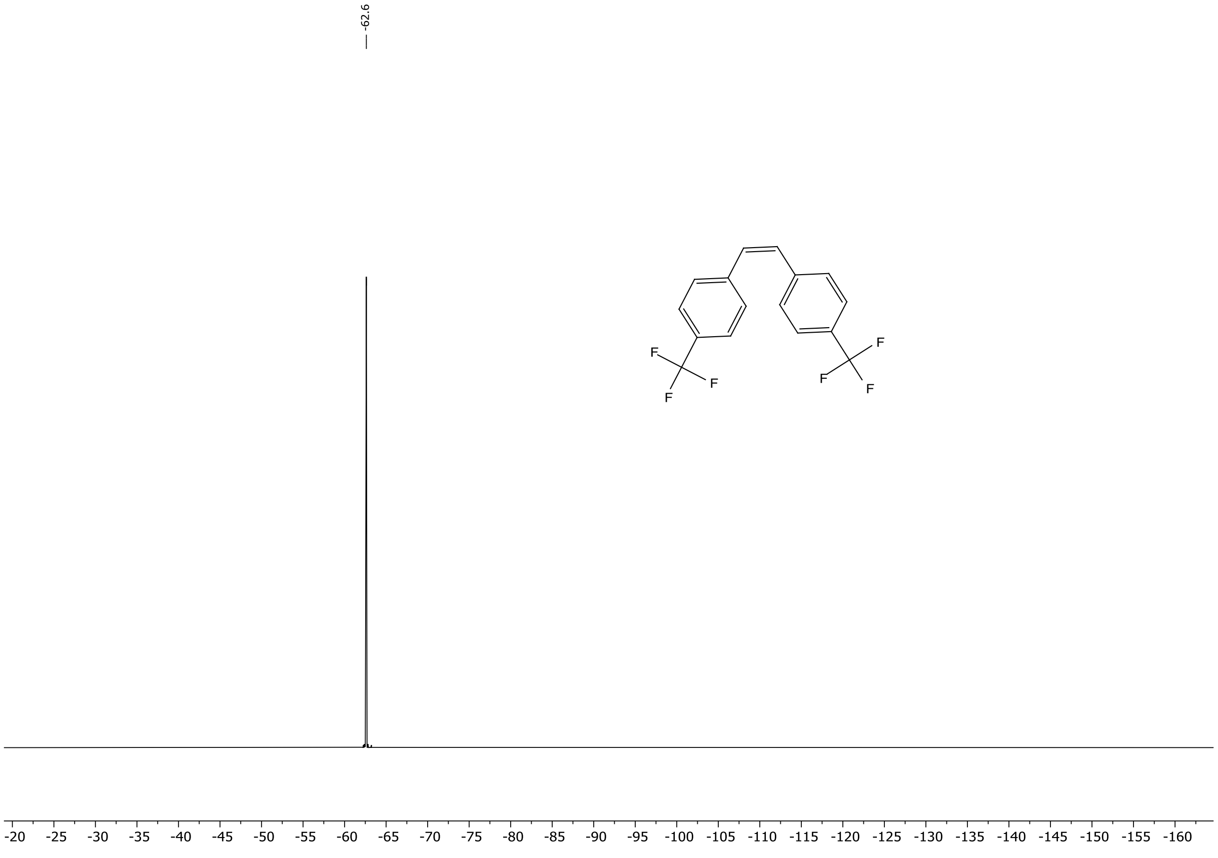
-62.6
F F F F F F
-20 -25 -30 -35 -40 -45 -50 -55 -60 -65 -70 -75 -80 -85 -90 -95 -100 -105 -110 -115 -120 -125 -130 -135 -140 -145 -150 -155 -160

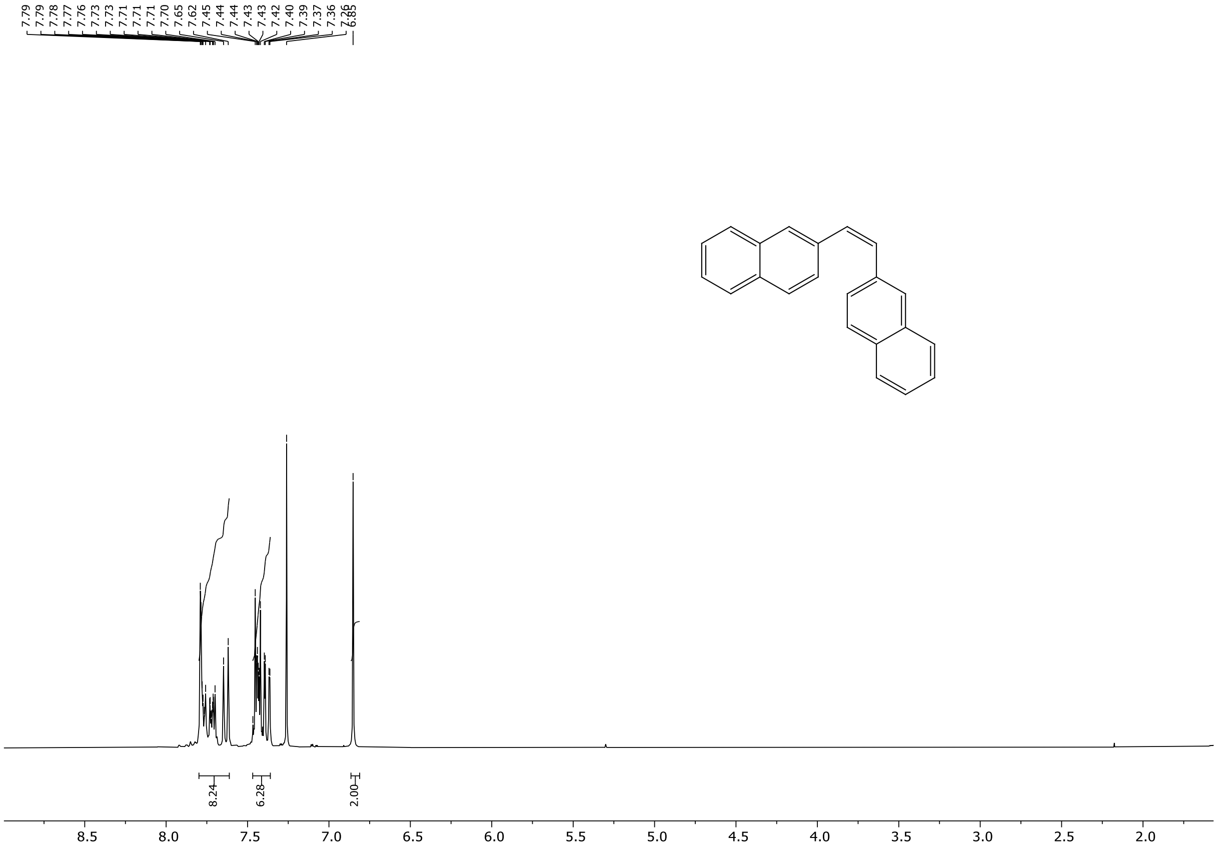
7.79 7.79 7.78 7.77 7.76 7.73 7.73 7.71 7.71 7.71 7.70 7.65 7.62 7.45 7.44 7.44 7.43 7.43 7.42 7.40 7.39 7.37 7.36 7.26 6.85
8.24
6.28
2.00
8.5 8.0 7.5 7.0 6.5 6.0 5.5 5.0 4.5 4.0 3.5 3.0 2.5 2.0

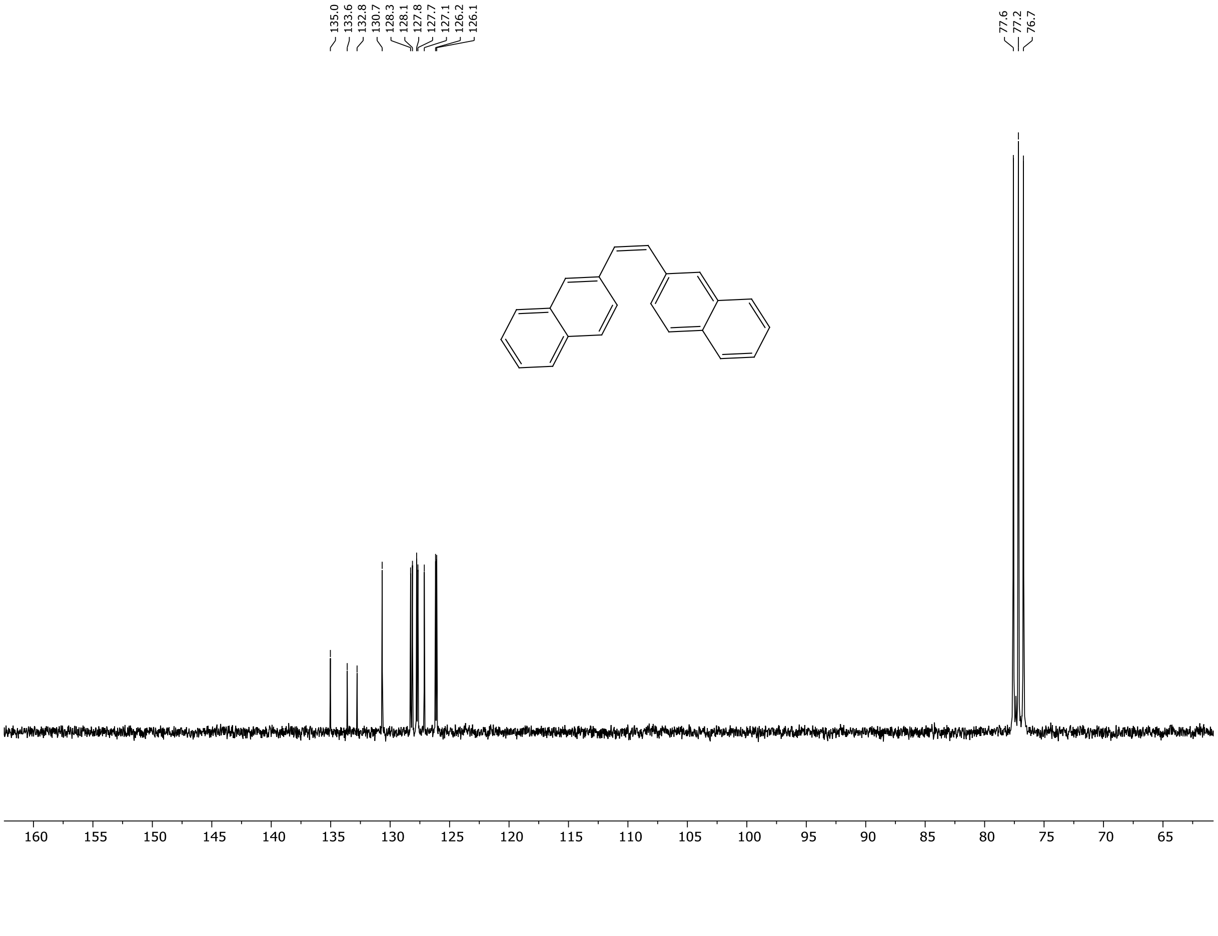
135.0 133.6 132.8 130.7 128.3 128.1 127.8 127.7 127.1 126.2 126.1
77.6 77.2 76.7
160 155 150 145 140 135 130 125 120 115 110 105 100 95 90 85 80 75 70 65

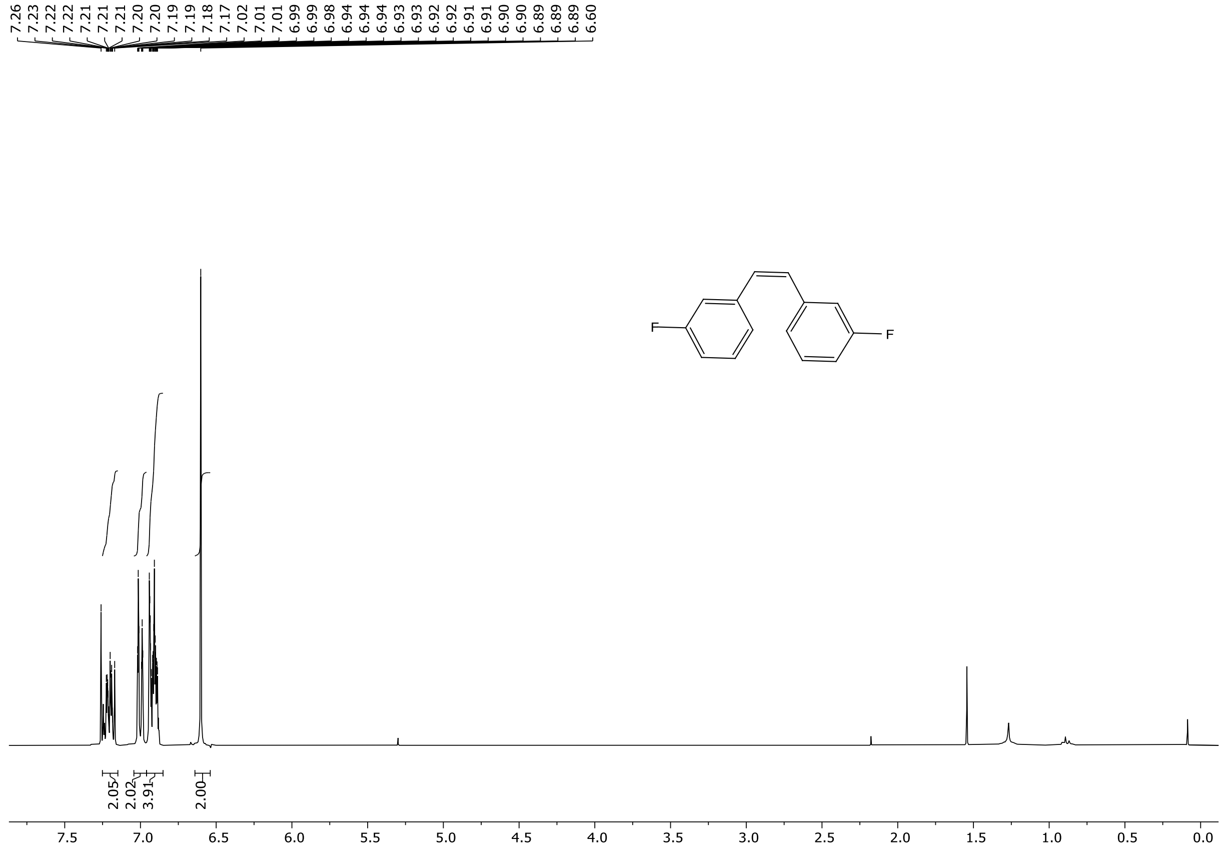
7.26
7.23
7.22
7.22
7.21
7.21
7.21
7.20
7.20
7.19
7.19
7.18
7.17
7.02
7.01
7.01
6.99
6.99
6.98
6.94
6.94
6.94
6.93
6.93
6.92
6.92
6.91
6.91
6.90
6.90
6.89
6.89
6.60
F
F
2.05
2.02
3.91
2.00
7.5
7.0
6.5
6.0
5.5
5.0
4.5
4.0
3.5
3.0
2.5
2.0
1.5
1.0
0.5
0.0

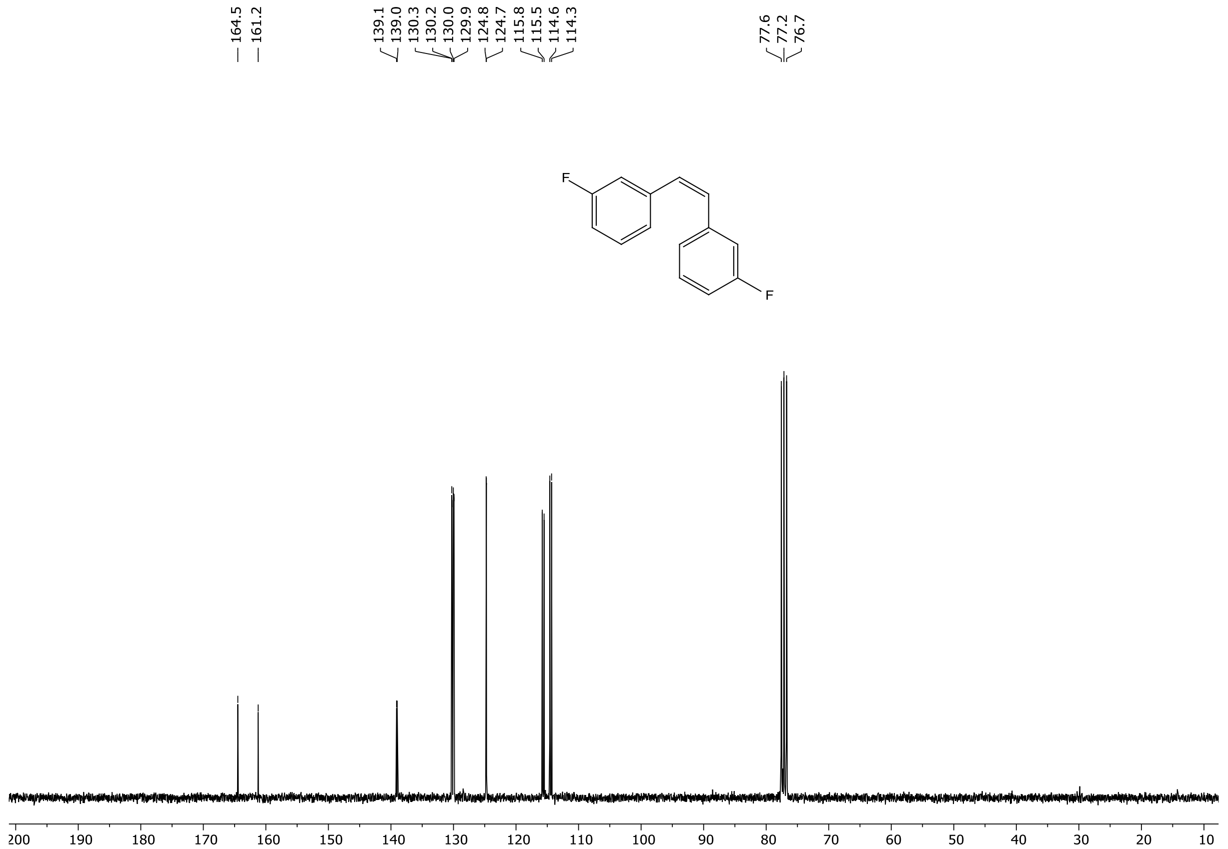
164.5
161.2
139.1
139.0
130.3
130.2
130.0
129.9
124.8
124.7
115.8
115.5
114.6
114.3
77.6
77.2
76.7
F
F
200
190
180
170
160
150
140
130
120
110
100
90
80
70
60
50
40
30
20
10

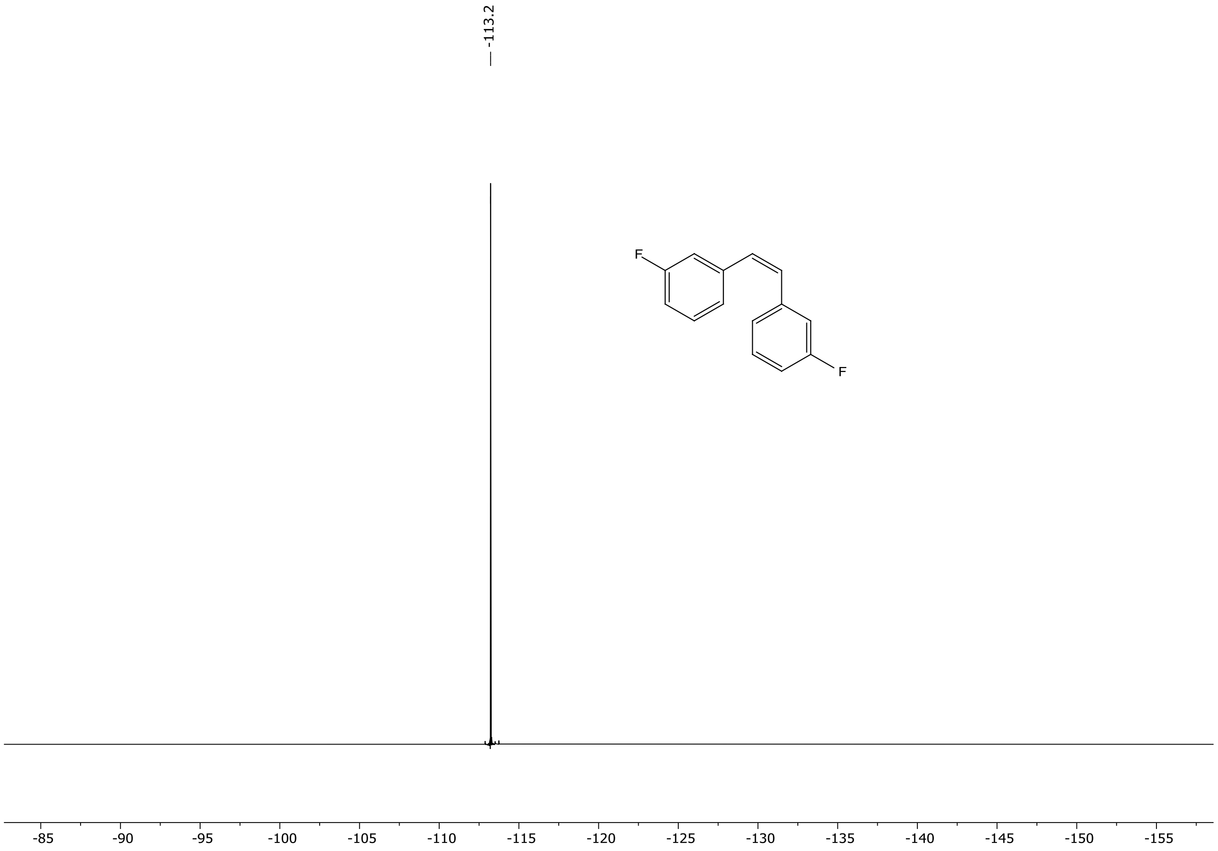

-113.2
F
F
-85
-90
-95
-100
-105
-110
-115
-120
-125
-130
-135
-140
-145
-150
-155

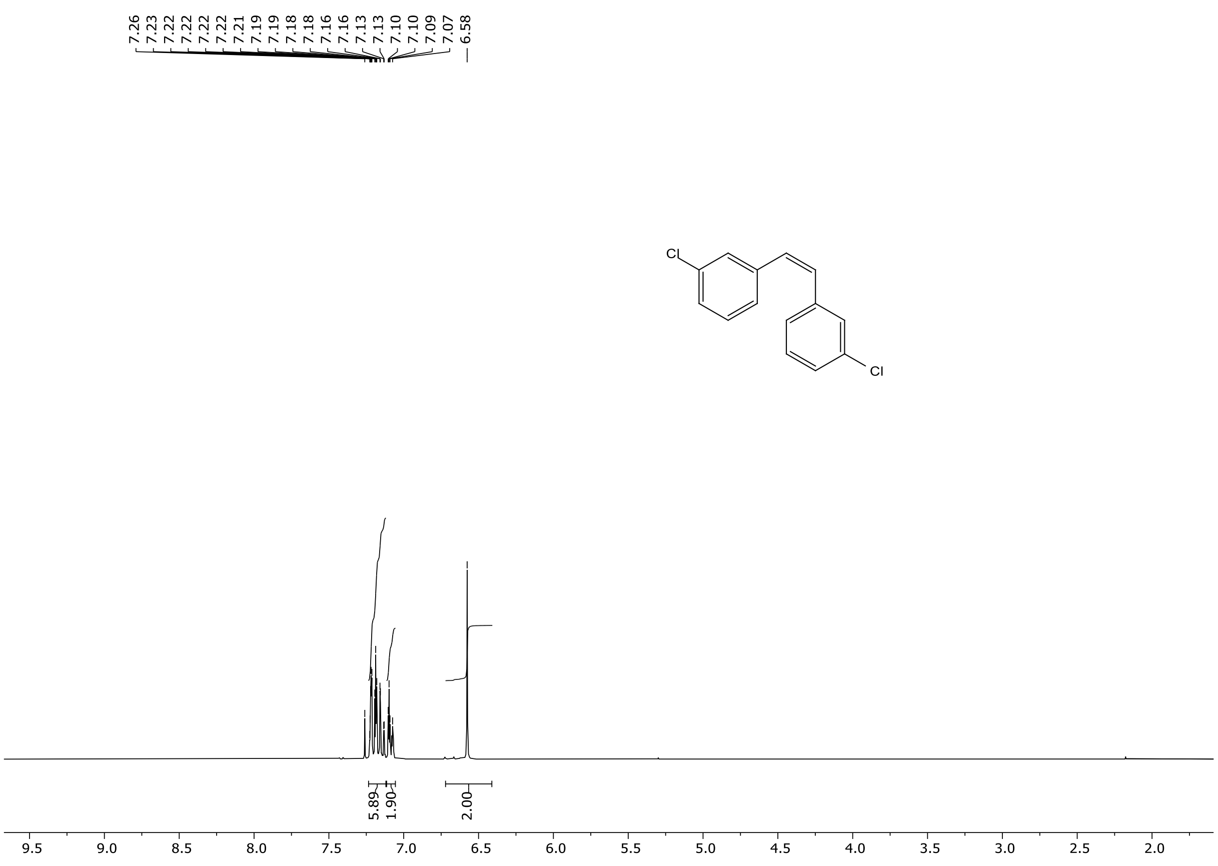
7.26
7.23
7.22
7.22
7.22
7.22
7.21
7.19
7.19
7.18
7.18
7.16
7.16
7.13
7.13
7.10
7.10
7.09
7.07
6.58
Cl
Cl
5.89
1.90
2.00
9.5 9.0 8.5 8.0 7.5 7.0 6.5 6.0 5.5 5.0 4.5 4.0 3.5 3.0 2.5 2.0

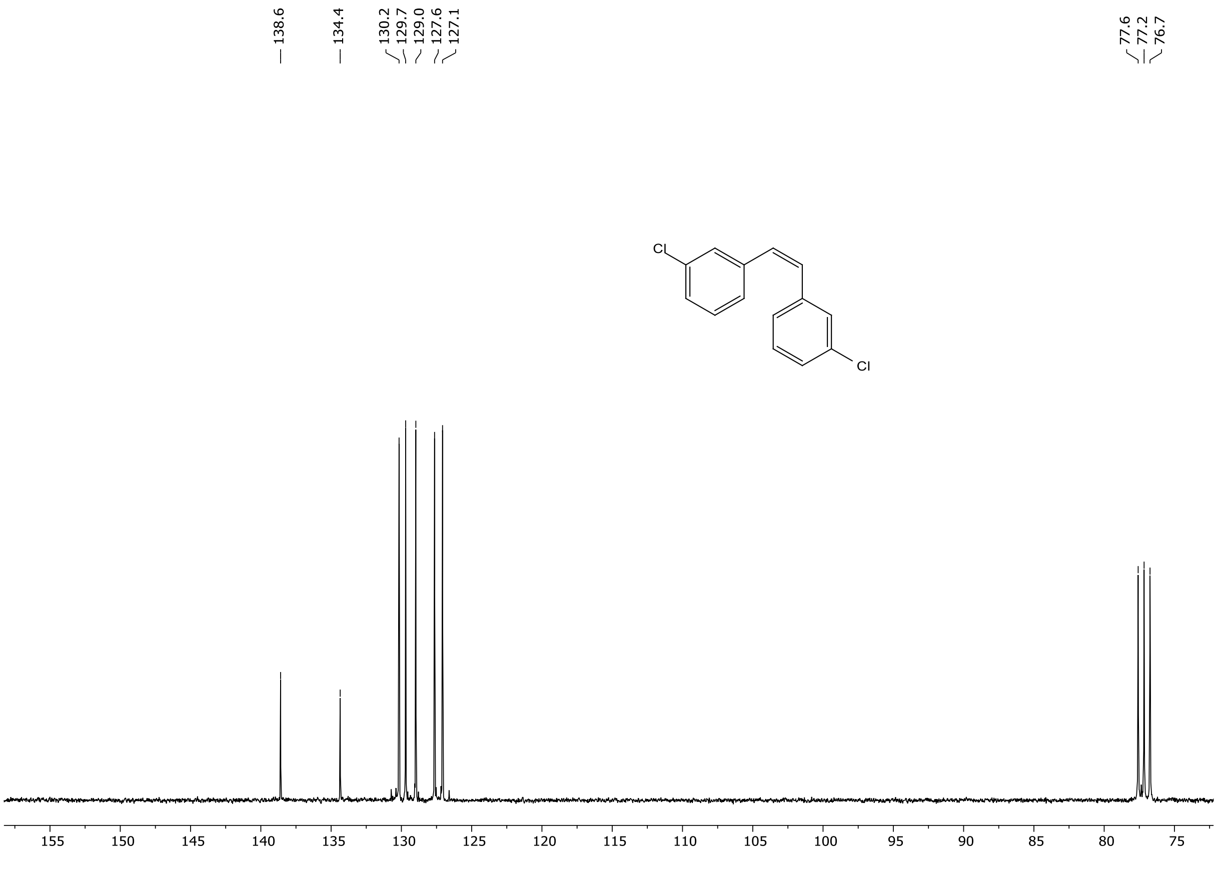
138.6
134.4
130.2
129.7
129.0
127.6
127.1
77.6
77.2
76.7
Cl
Cl
155 150 145 140 135 130 125 120 115 110 105 100 95 90 85 80 75

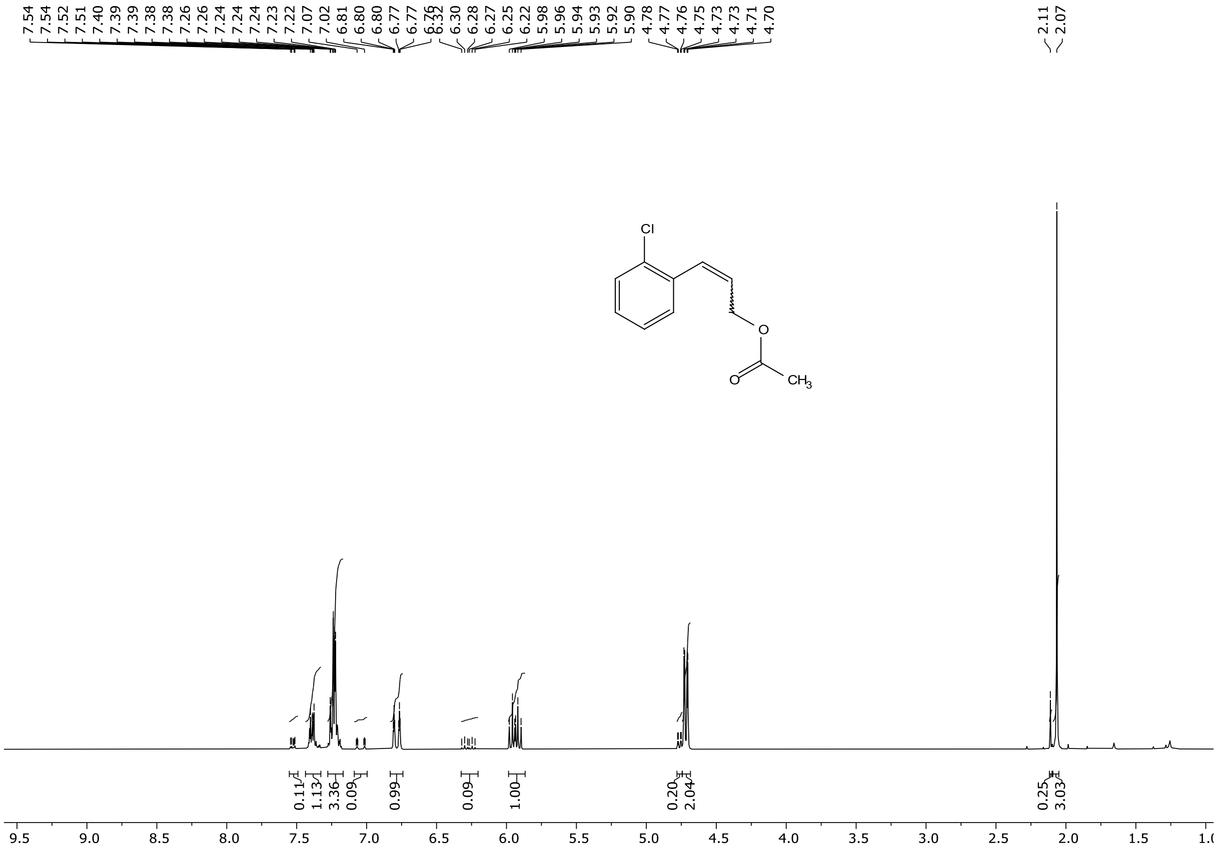

7.54
7.54
7.52
7.51
7.40
7.39
7.39
7.38
7.38
7.26
7.26
7.24
7.24
7.24
7.23
7.22
7.07
7.02
6.81
6.80
6.80
6.77
6.77
6.76
6.32
6.30
6.28
6.27
6.25
6.22
5.98
5.96
5.94
5.93
5.92
5.90
4.78
4.77
4.76
4.75
4.73
4.73
4.71
4.70
2.11
2.07
Cl
O
O
CH3
0.11
1.13
3.36
0.09
0.99
0.09
1.00
0.20
2.04
0.25
3.03
9.5
9.0
8.5
8.0
7.5
7.0
6.5
6.0
5.5
5.0
4.5
4.0
3.5
3.0
2.5
2.0
1.5

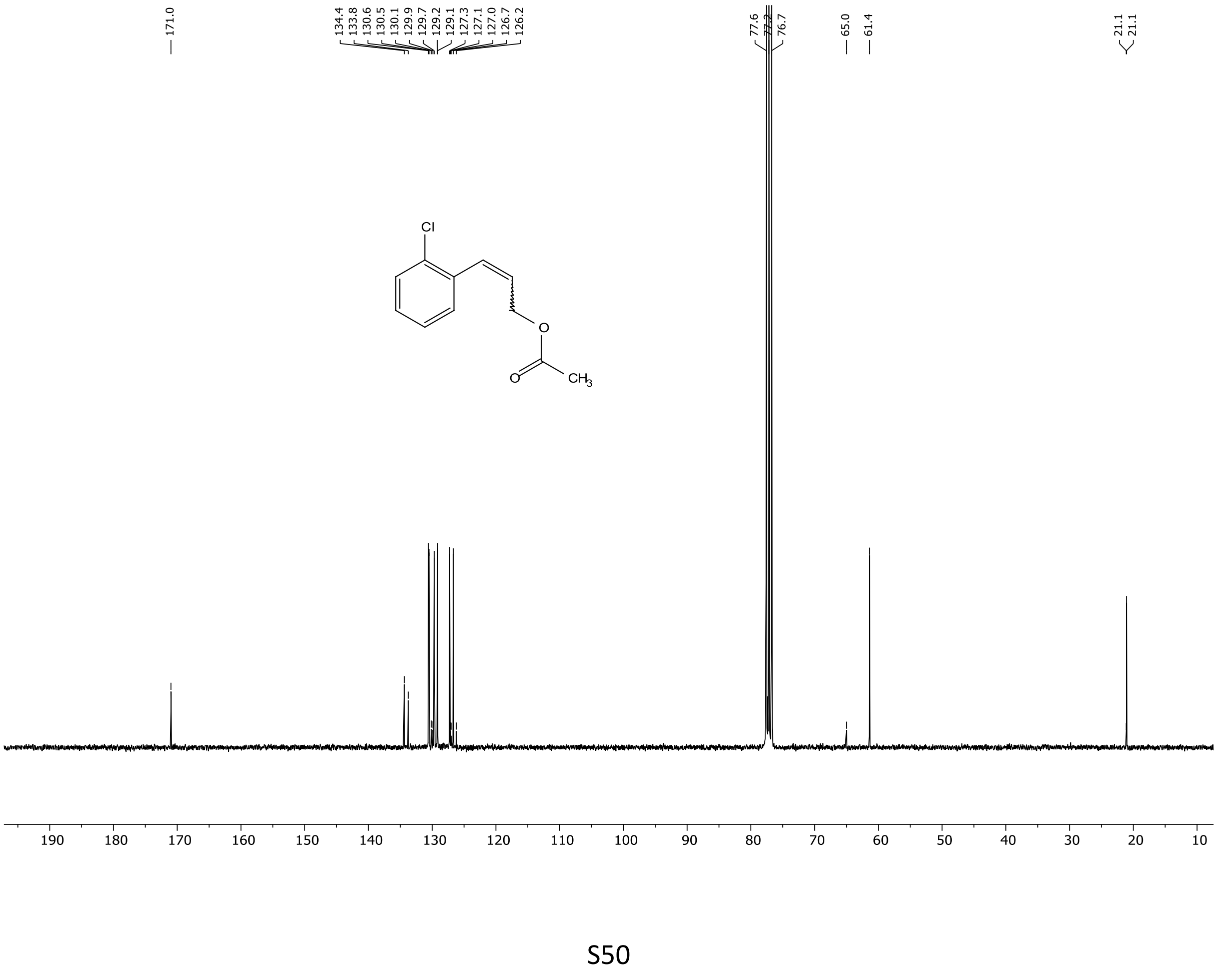

171.0
134.4
133.8
130.6
130.5
130.1
129.9
129.7
129.2
129.1
127.3
127.1
127.0
126.7
126.2
77.6
77.2
76.7
65.0
61.4
21.1
21.1
Cl
O
O
CH3
190
180
170
160
150
140
130
120
110
100
90
80
70
60
50
40
30
20
10

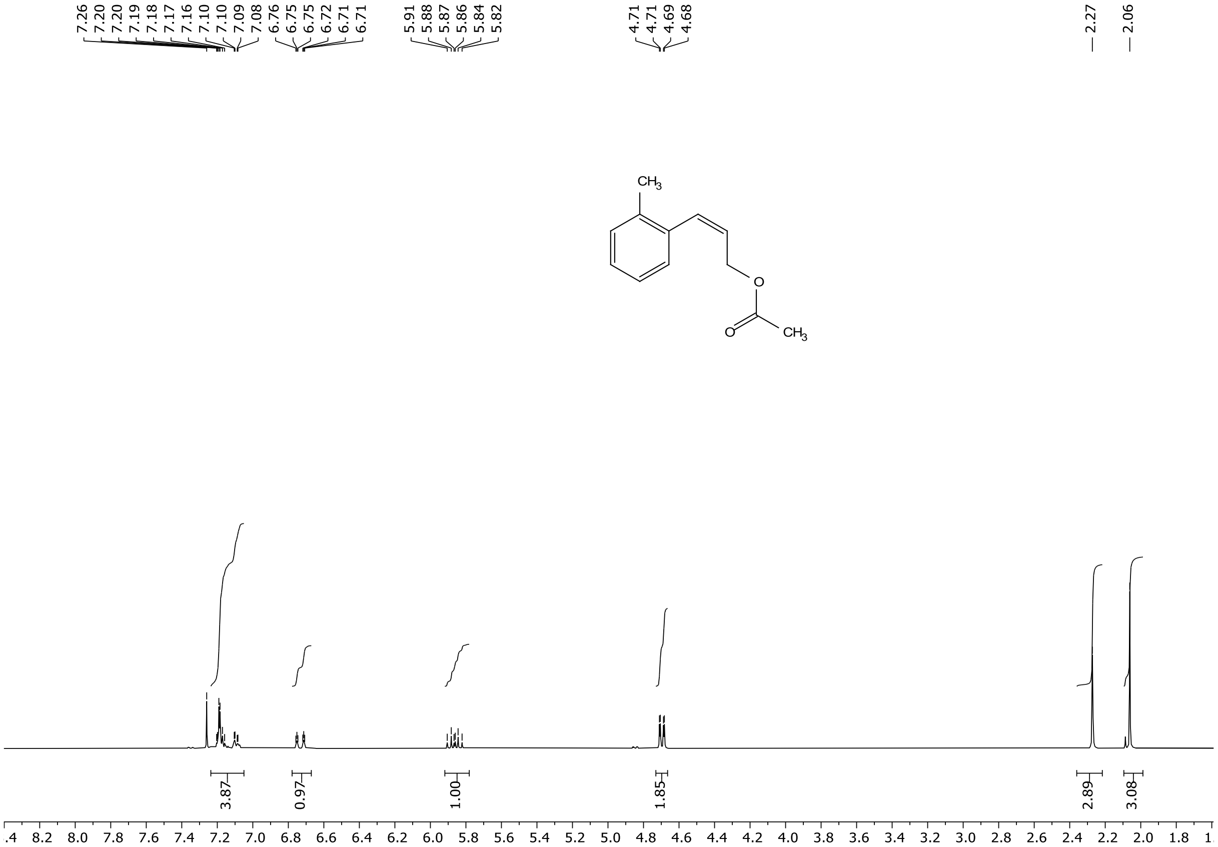
7.26 7.20 7.20 7.19 7.18 7.17 7.16 7.10 7.09 7.08 6.76 6.75 6.75 6.72 6.71 6.71
5.91 5.88 5.87 5.86 5.84 5.82
4.71 4.71 4.69 4.68
2.27
2.06
CH3
O
O
CH3
3.87
0.97
1.00
1.85
2.89
3.08
.4 8.2 8.0 7.8 7.6 7.4 7.2 7.0 6.8 6.6 6.4 6.2 6.0 5.8 5.6 5.4 5.2 5.0 4.8 4.6 4.4 4.2 4.0 3.8 3.6 3.4 3.2 3.0 2.8 2.6 2.4 2.2 2.0 1.8 1.

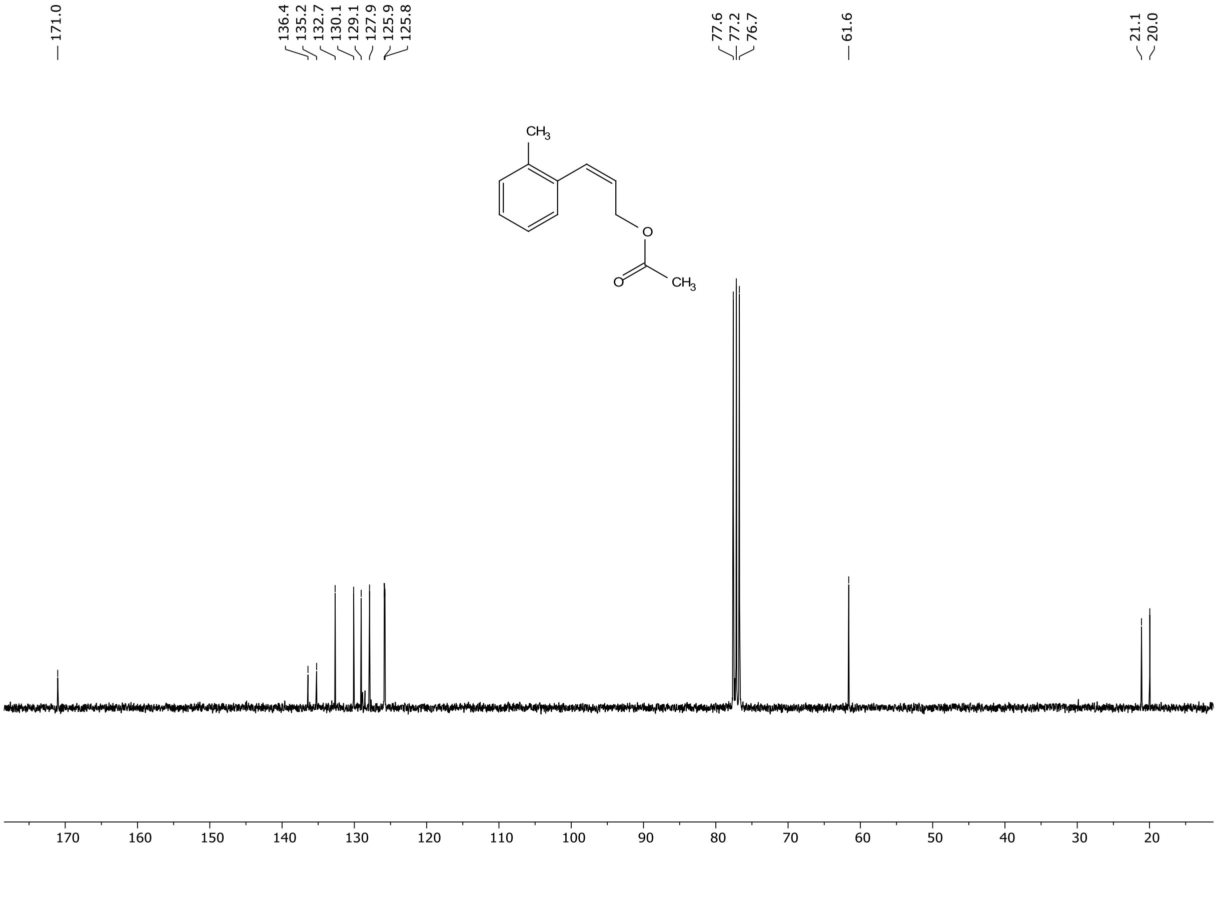
171.0
136.4 135.2 132.7 130.1 129.1 127.9 125.9 125.8
77.6 77.2 76.7
61.6
21.1 20.0
CH3
O
O
CH3
170 160 150 140 130 120 110 100 90 80 70 60 50 40 30 20

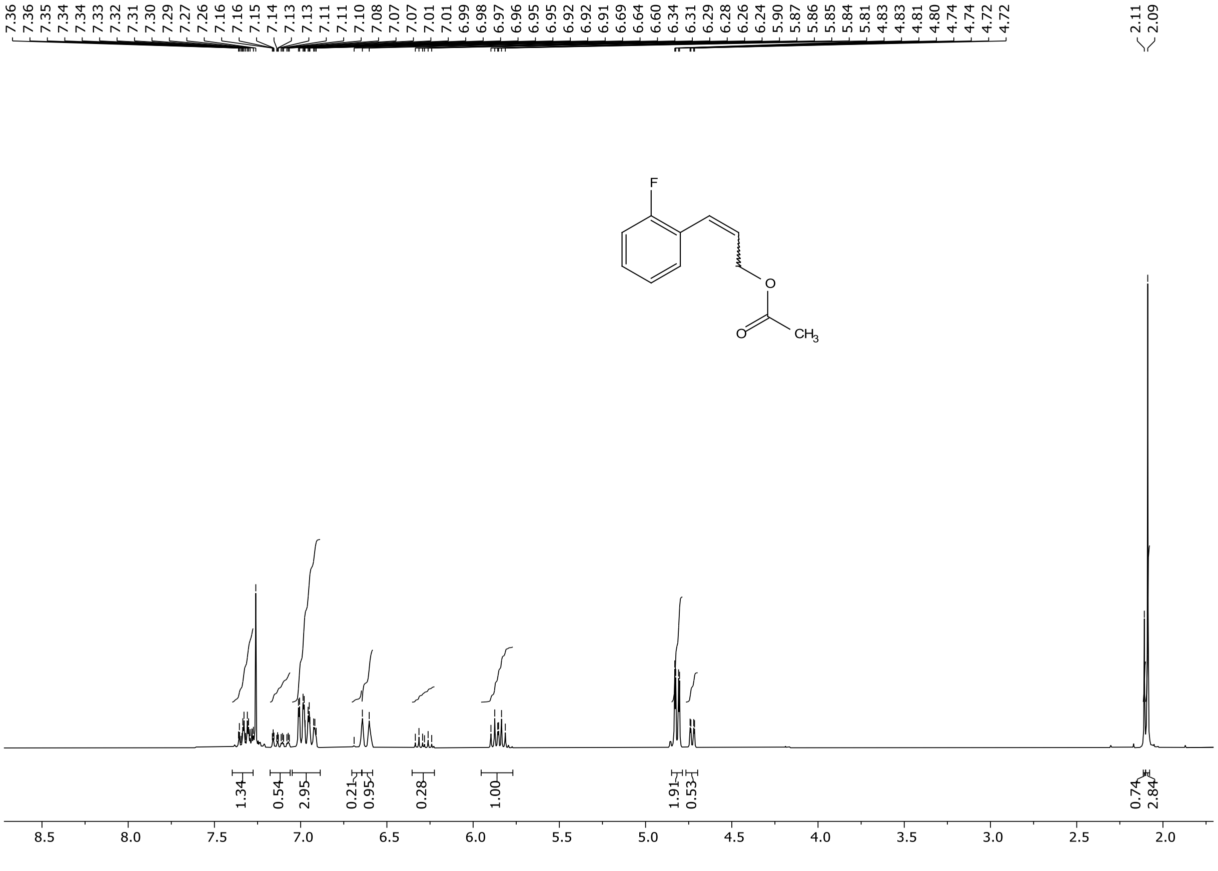

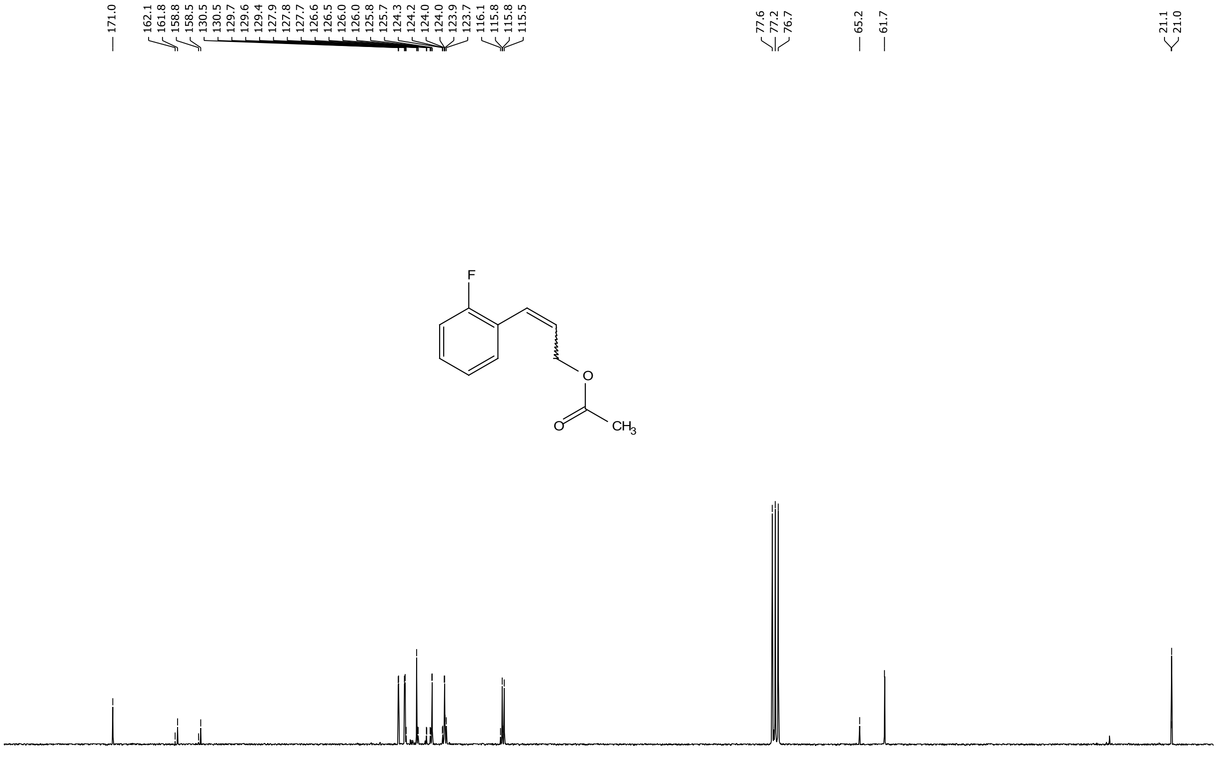

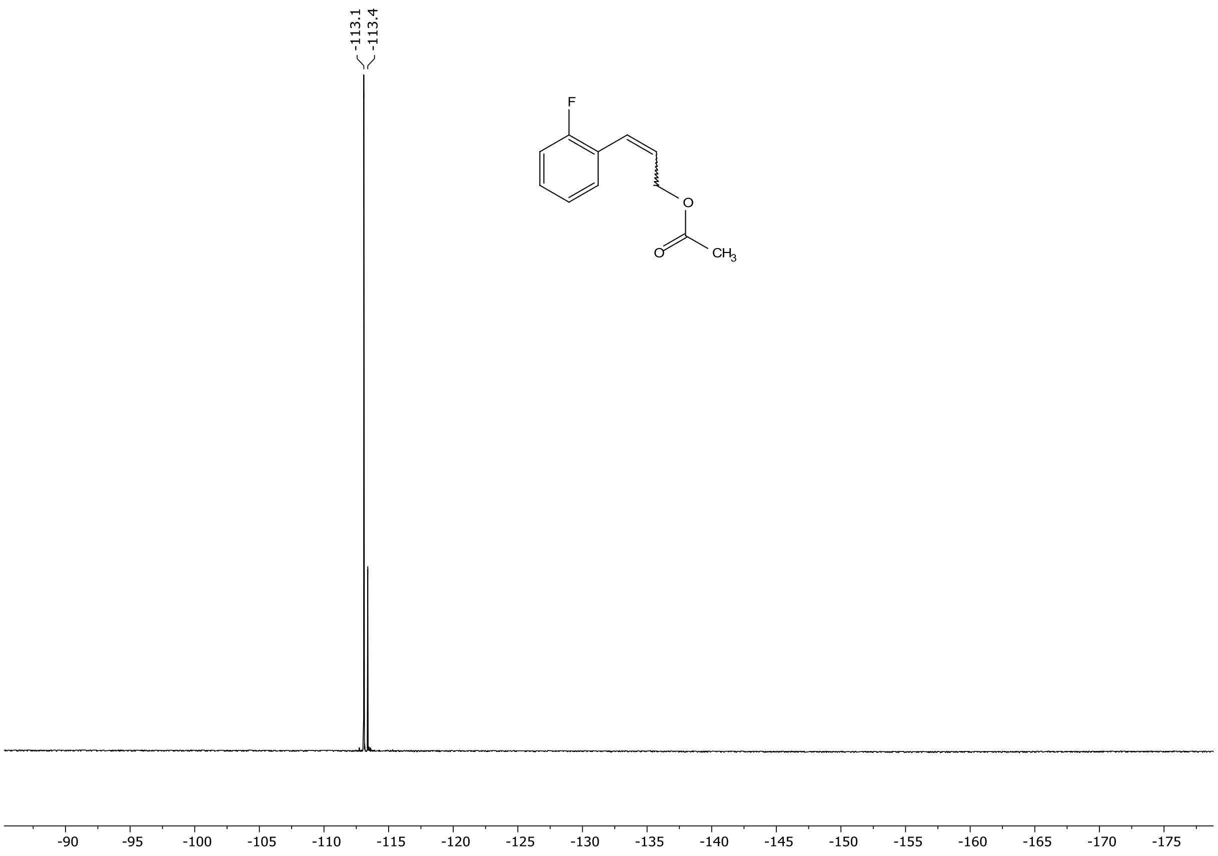
-113.1
-113.4
F
O
O
CH3
-90
-95
-100
-105
-110
-115
-120
-125
-130
-135
-140
-145
-150
-155
-160
-165
-170
-175

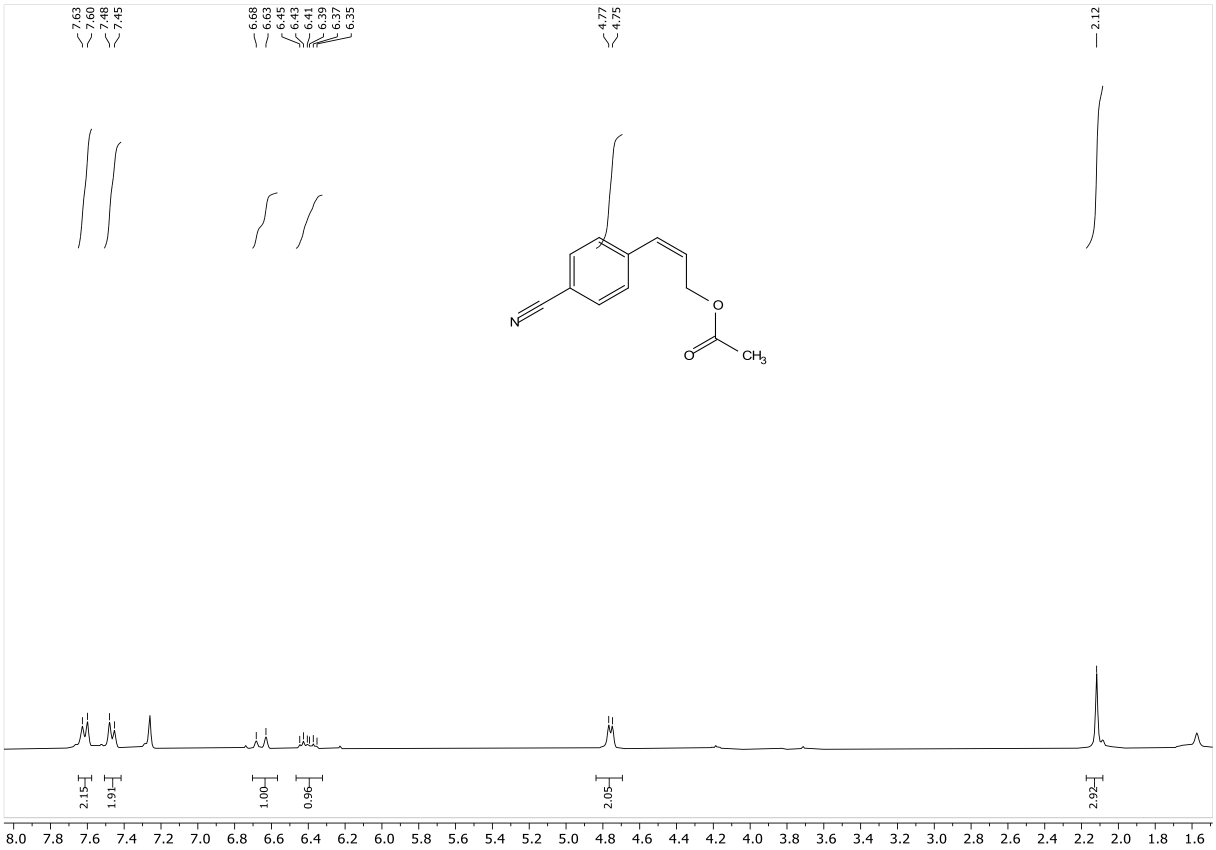
7.63
7.60
7.48
7.45
6.68
6.63
6.45
6.43
6.41
6.39
6.37
6.35
4.77
4.75
2.12
N
O
O
CH3
2.15
1.91
1.00
0.96
2.05
2.92
8.0 7.8 7.6 7.4 7.2 7.0 6.8 6.6 6.4 6.2 6.0 5.8 5.6 5.4 5.2 5.0 4.8 4.6 4.4 4.2 4.0 3.8 3.6 3.4 3.2 3.0 2.8 2.6 2.4 2.2 2.0 1.8 1.6

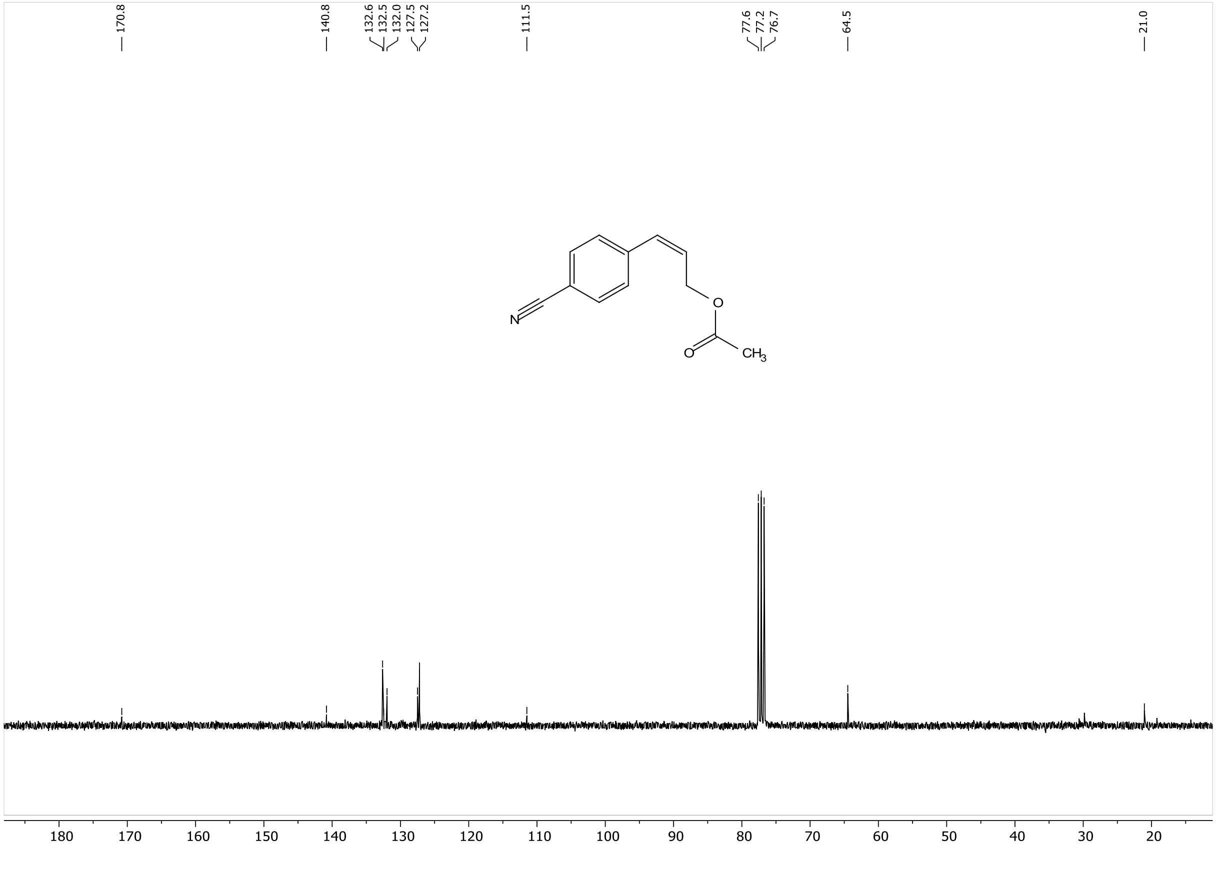
170.8
140.8
132.6
132.5
132.0
127.5
127.2
111.5
77.6
77.2
76.7
64.5
21.0
N
O
O
CH3
180 170 160 150 140 130 120 110 100 90 80 70 60 50 40 30 20

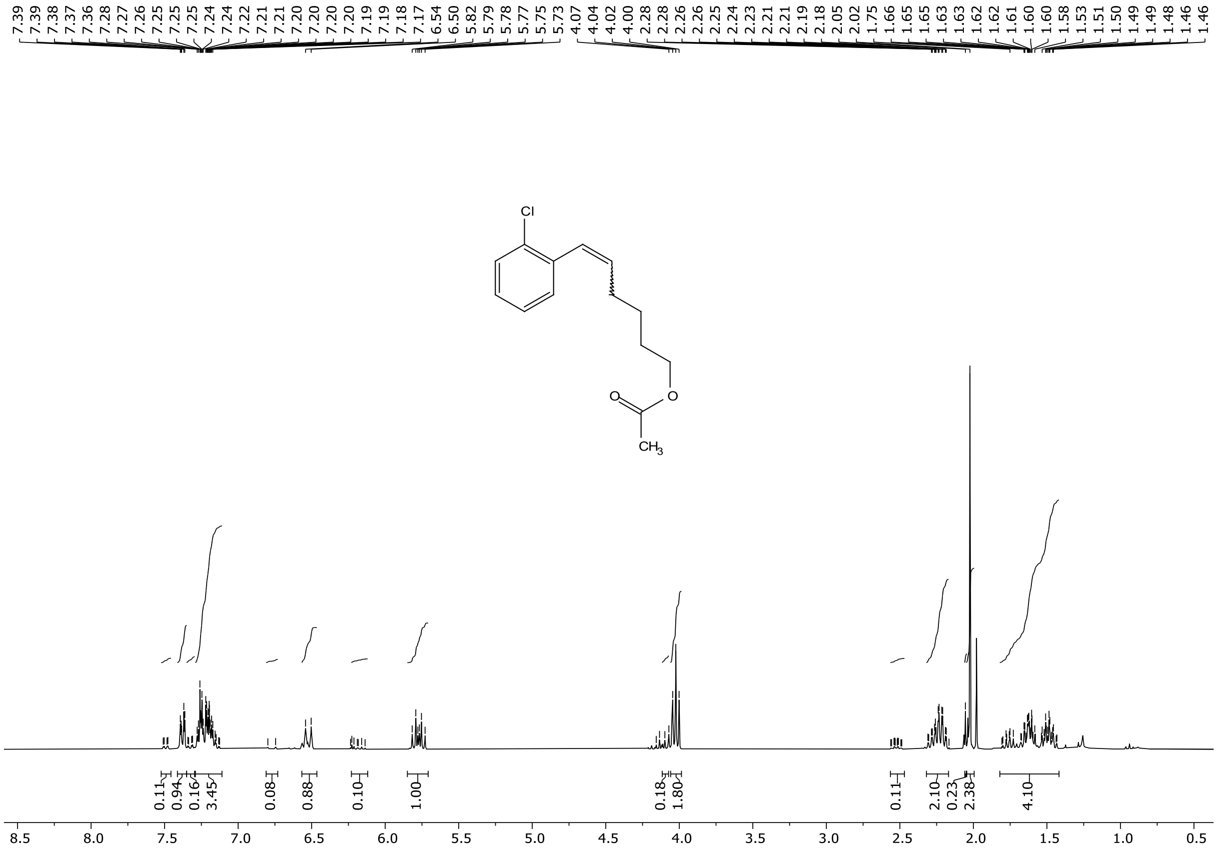

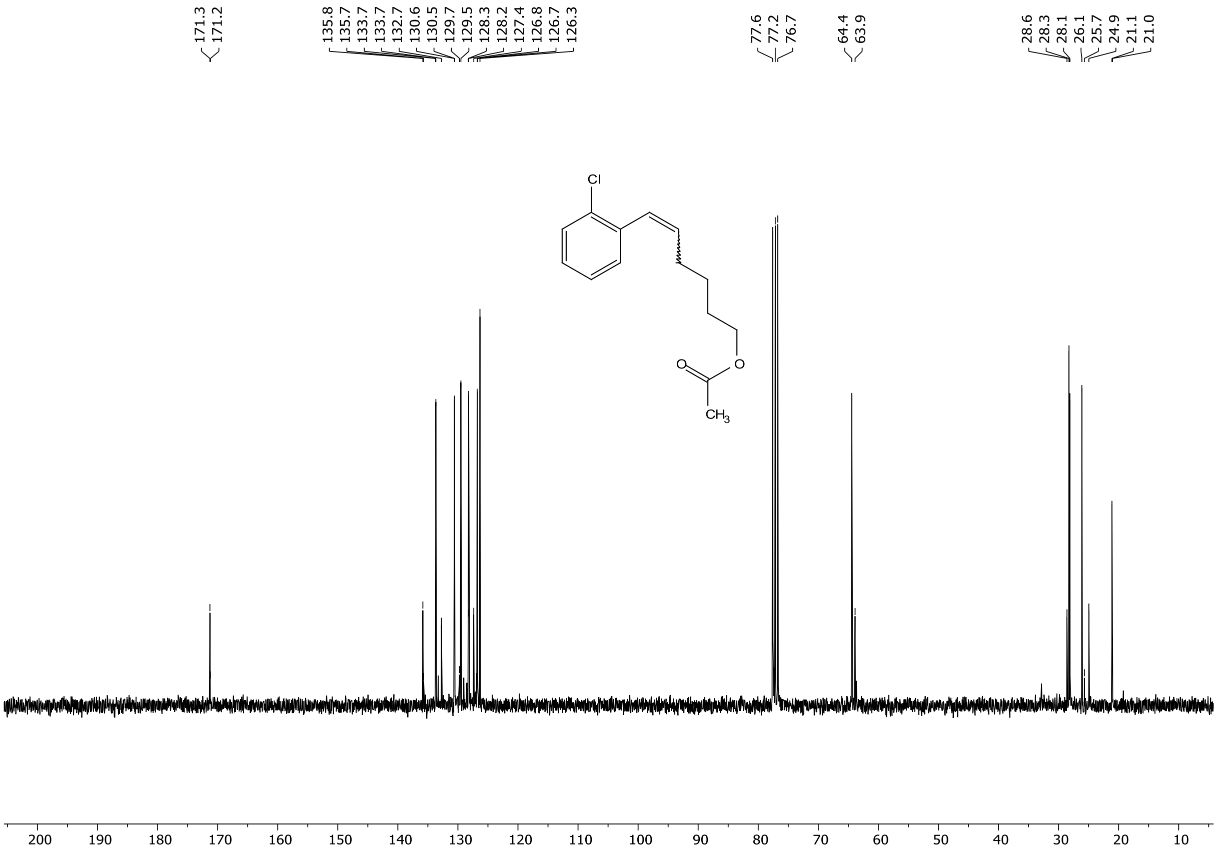

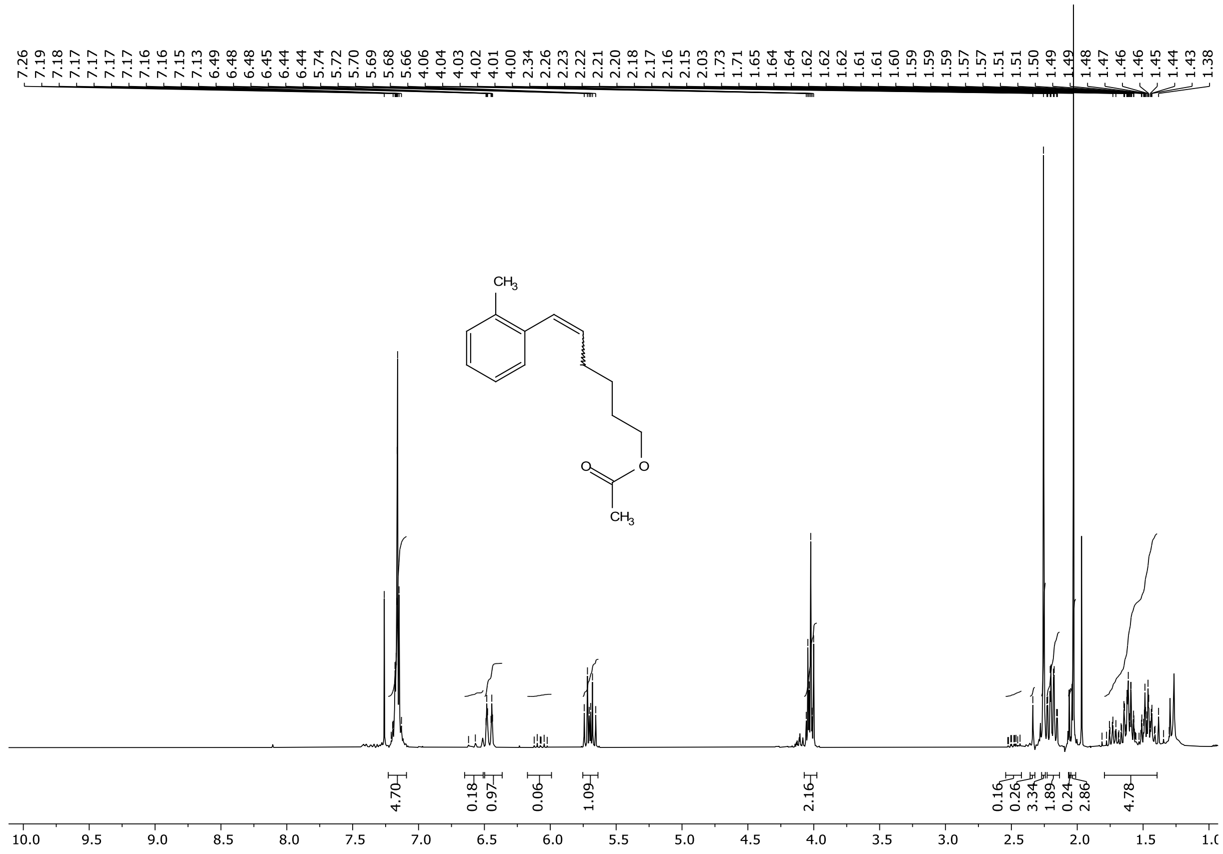

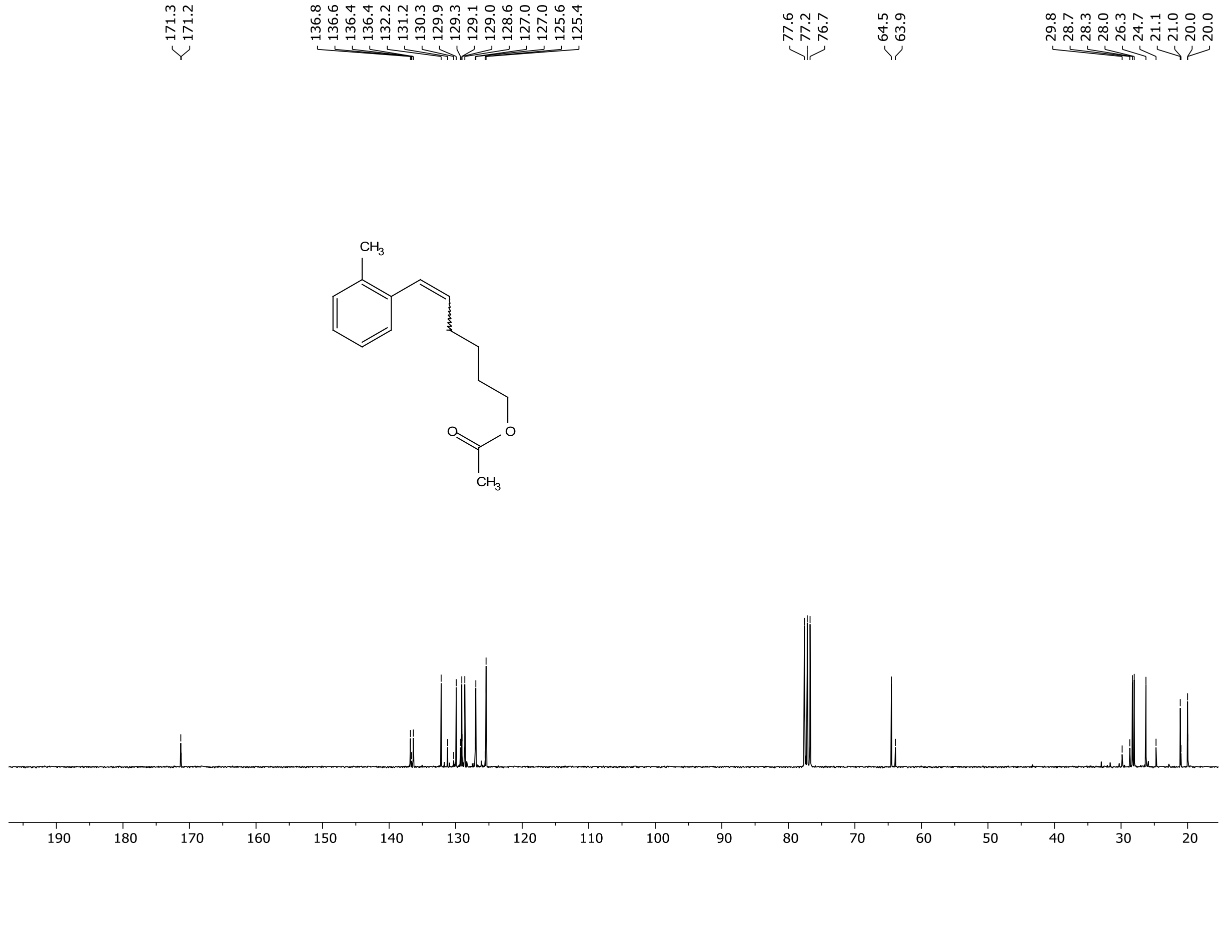

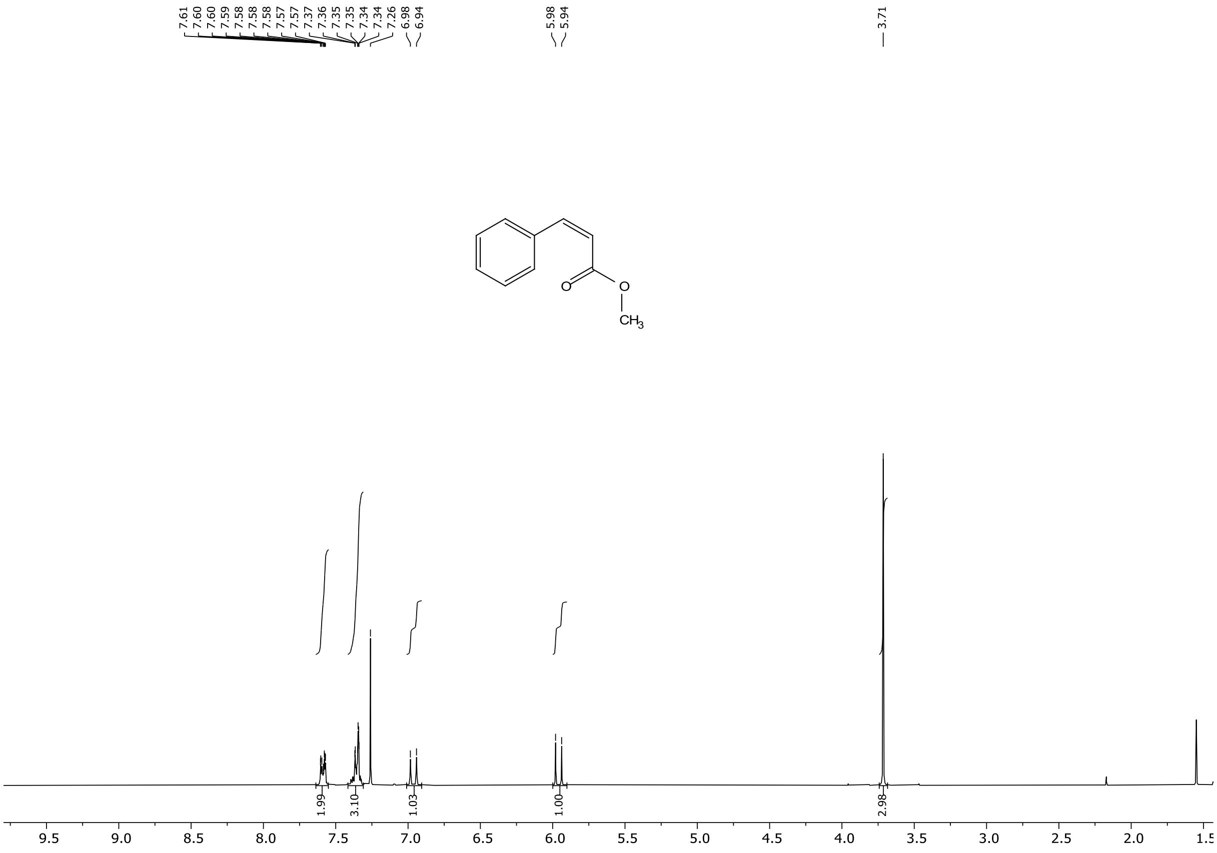
7.61 7.60 7.60 7.59 7.58 7.58 7.58 7.57 7.57 7.37 7.36 7.35 7.35 7.34 7.34 7.26 6.98 6.94
5.98 5.94
3.71
O
O
CH3
1.99
3.10
1.03
1.00
2.98
9.5 9.0 8.5 8.0 7.5 7.0 6.5 6.0 5.5 5.0 4.5 4.0 3.5 3.0 2.5 2.0

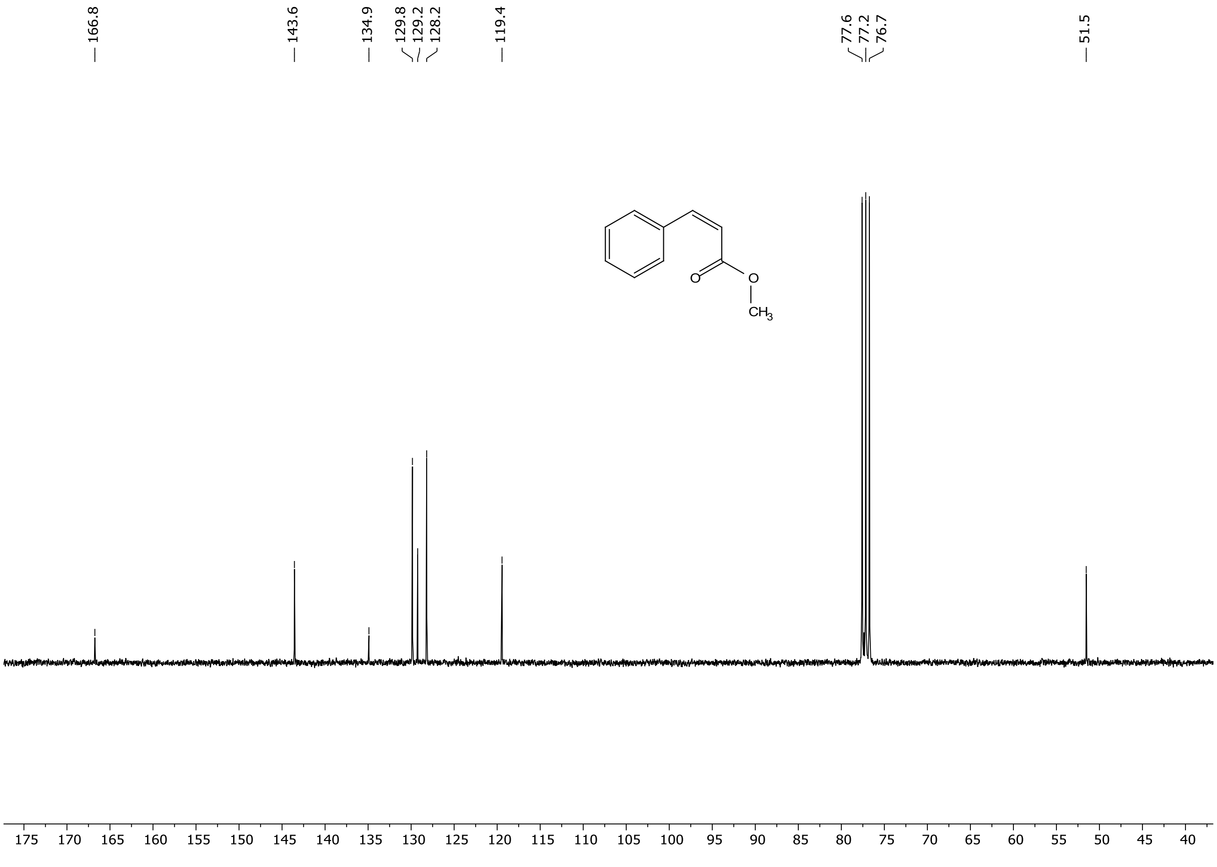
166.8
143.6
134.9
129.8 129.2 128.2
119.4
77.6 77.2 76.7
51.5
O
O
CH3
175 170 165 160 155 150 145 140 135 130 125 120 115 110 105 100 95 90 85 80 75 70 65 60 55 50 45 40

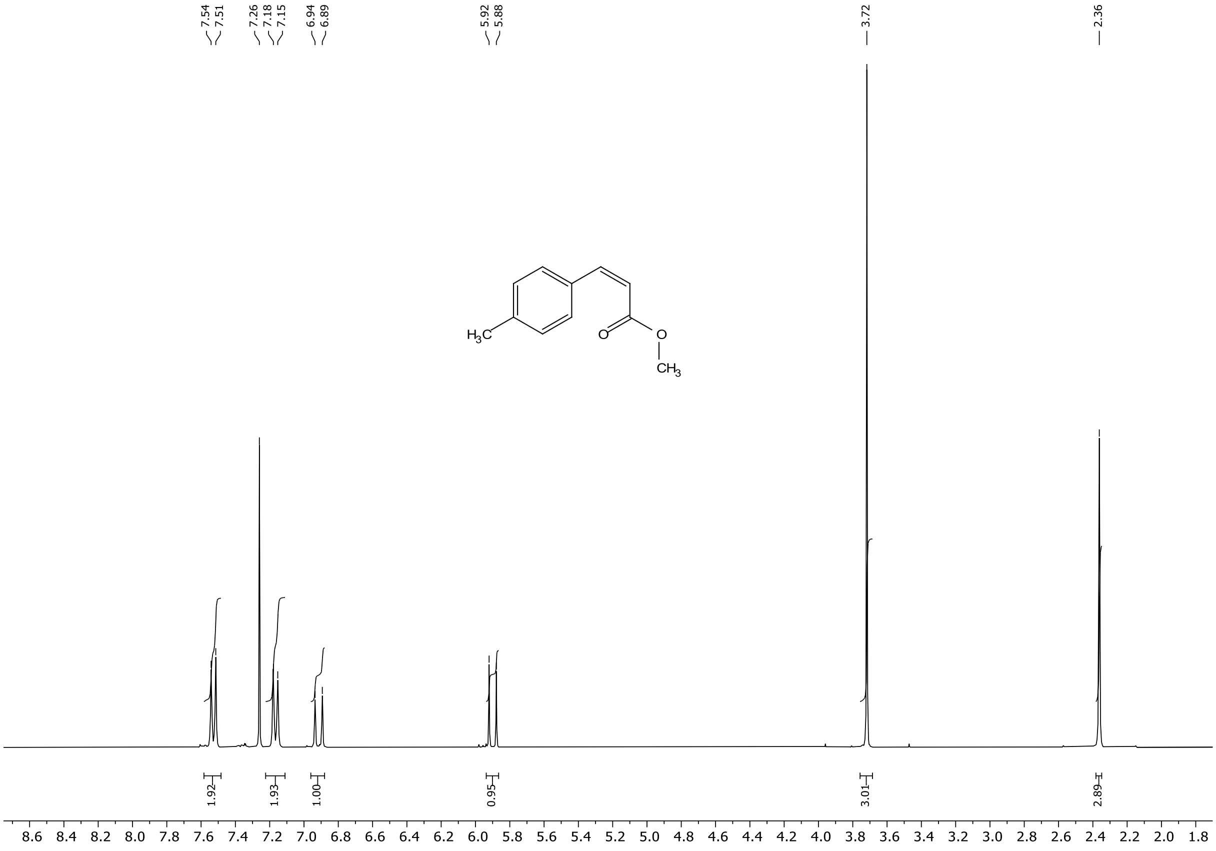
7.54
7.51
7.26
7.18
7.15
6.94
6.89
5.92
5.88
3.72
2.36
H3C
O
O
CH3
1.92
1.93
1.00
0.95
3.01
2.89
8.6 8.4 8.2 8.0 7.8 7.6 7.4 7.2 7.0 6.8 6.6 6.4 6.2 6.0 5.8 5.6 5.4 5.2 5.0 4.8 4.6 4.4 4.2 4.0 3.8 3.6 3.4 3.2 3.0 2.8 2.6 2.4 2.2 2.0 1.8

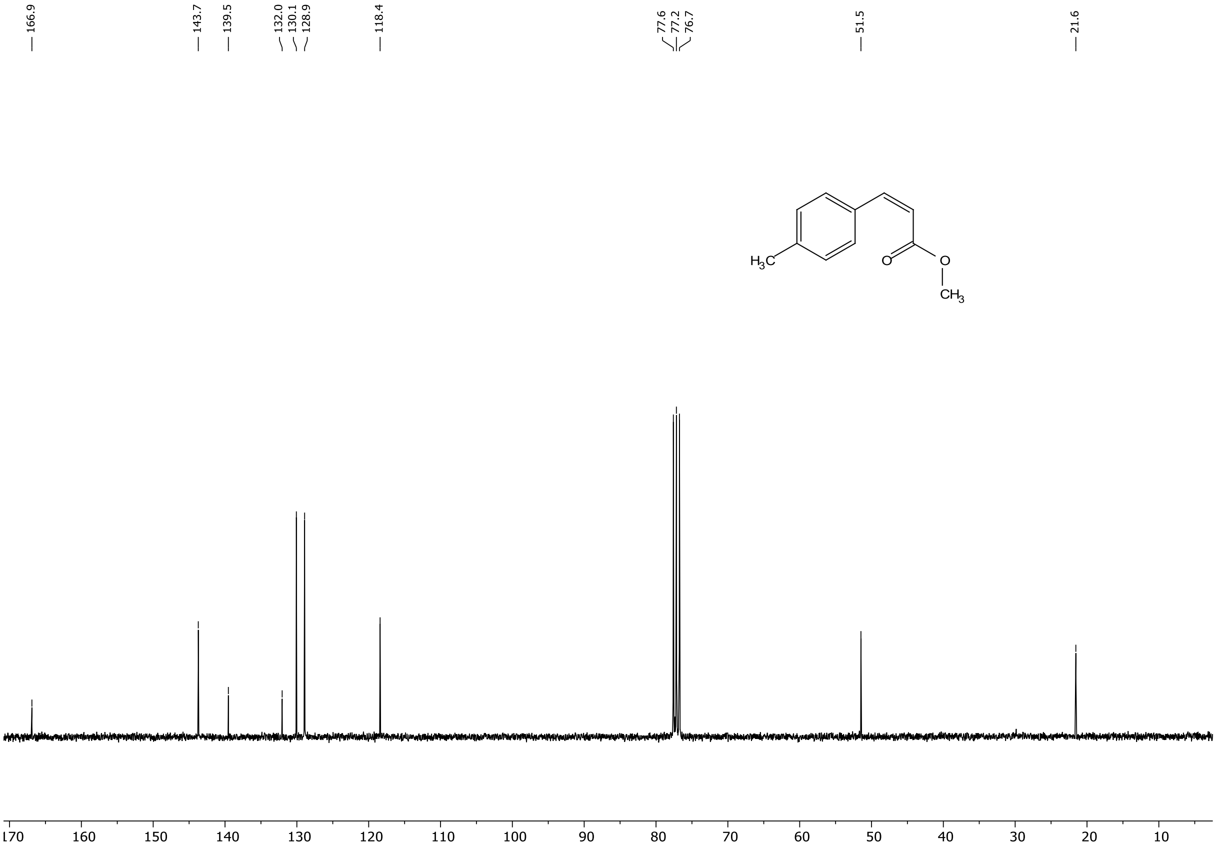
166.9
143.7
139.5
132.0
130.1
128.9
118.4
77.6
77.2
76.7
51.5
21.6
H3C
O
O
CH3
170 160 150 140 130 120 110 100 90 80 70 60 50 40 30 20 10

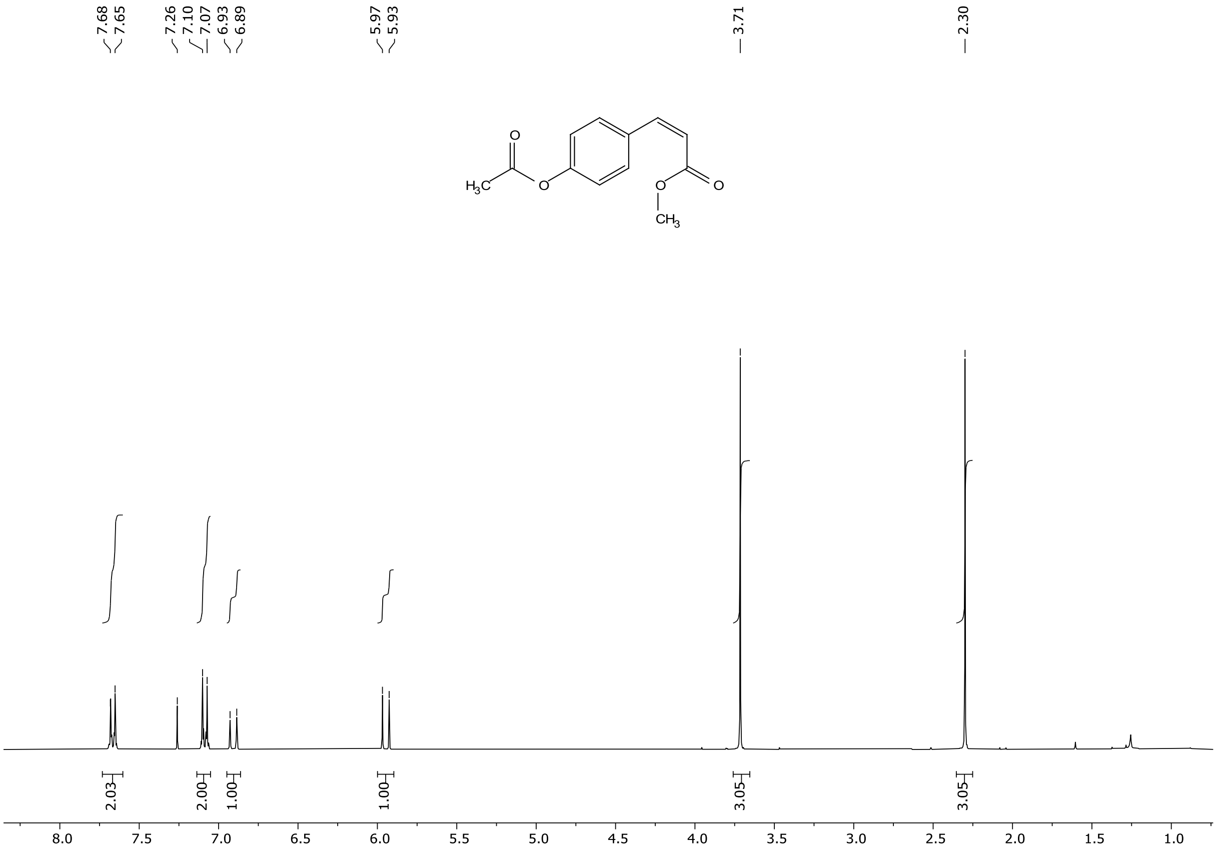
7.68
7.65
7.26
7.10
7.07
6.93
6.89
5.97
5.93
3.71
2.30
O
H3C
O
O
O
CH3
2.03
2.00
1.00
1.00
3.05
3.05
8.0 7.5 7.0 6.5 6.0 5.5 5.0 4.5 4.0 3.5 3.0 2.5 2.0 1.5 1.0

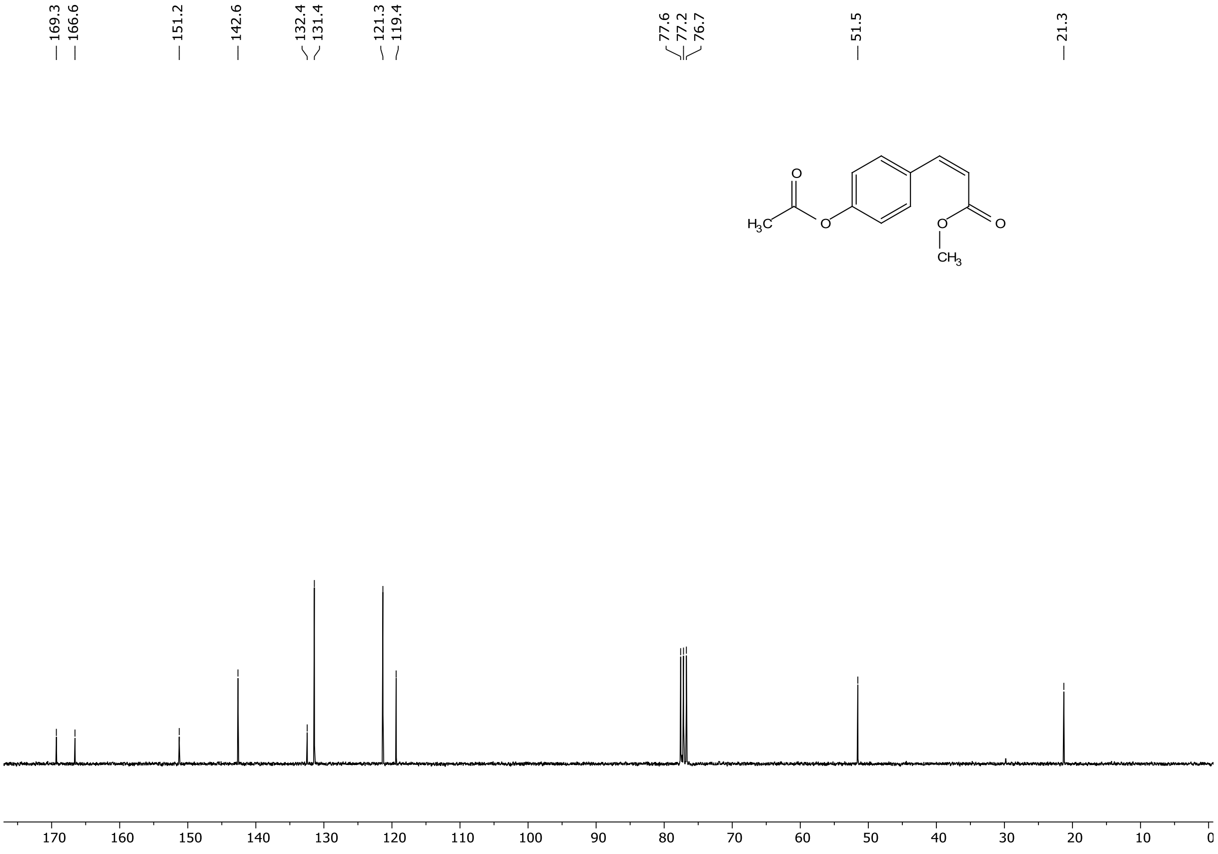
169.3
166.6
151.2
142.6
132.4
131.4
121.3
119.4
77.6
77.2
76.7
51.5
21.3
O
H3C
O
O
O
CH3
170 160 150 140 130 120 110 100 90 80 70 60 50 40 30 20 10 0

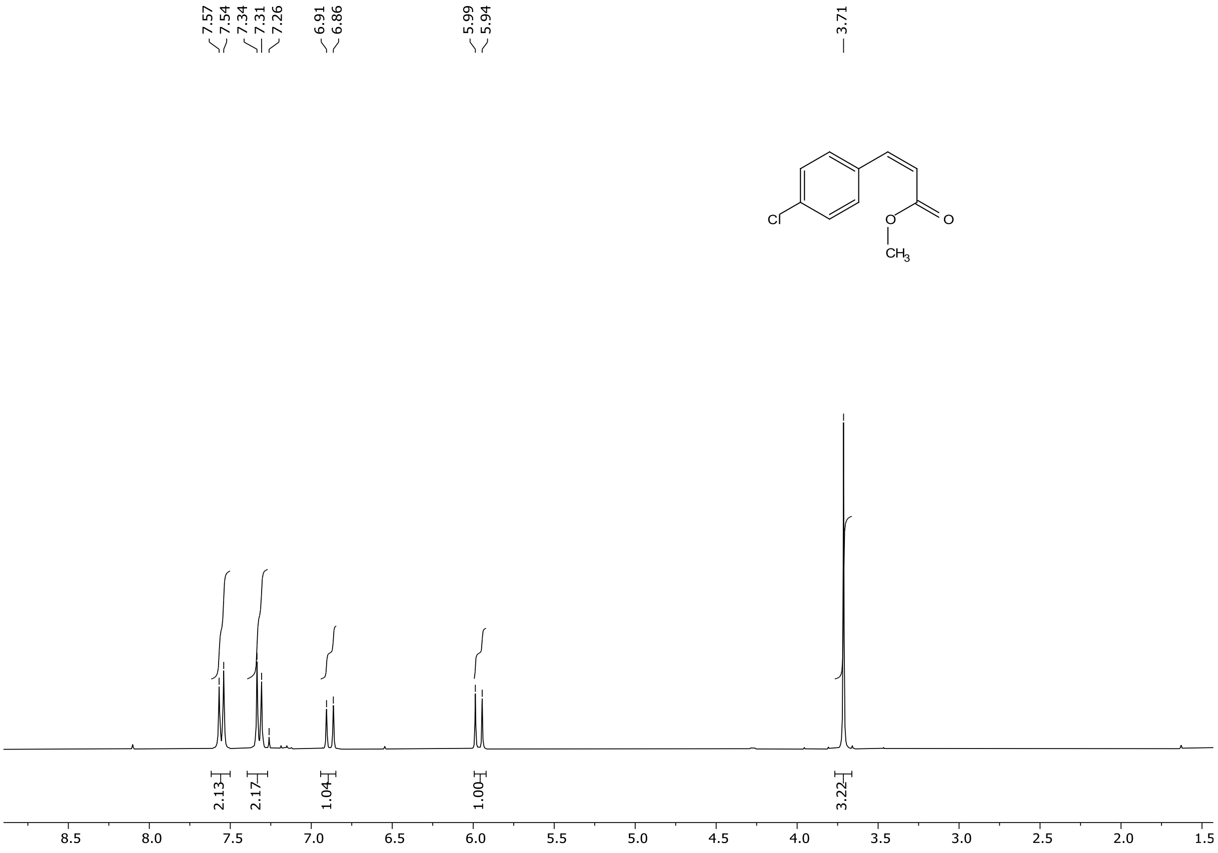
7.57
7.54
7.34
7.31
7.26
6.91
6.86
5.99
5.94
3.71
Cl
O
O
CH3
2.13
2.17
1.04
1.00
3.22

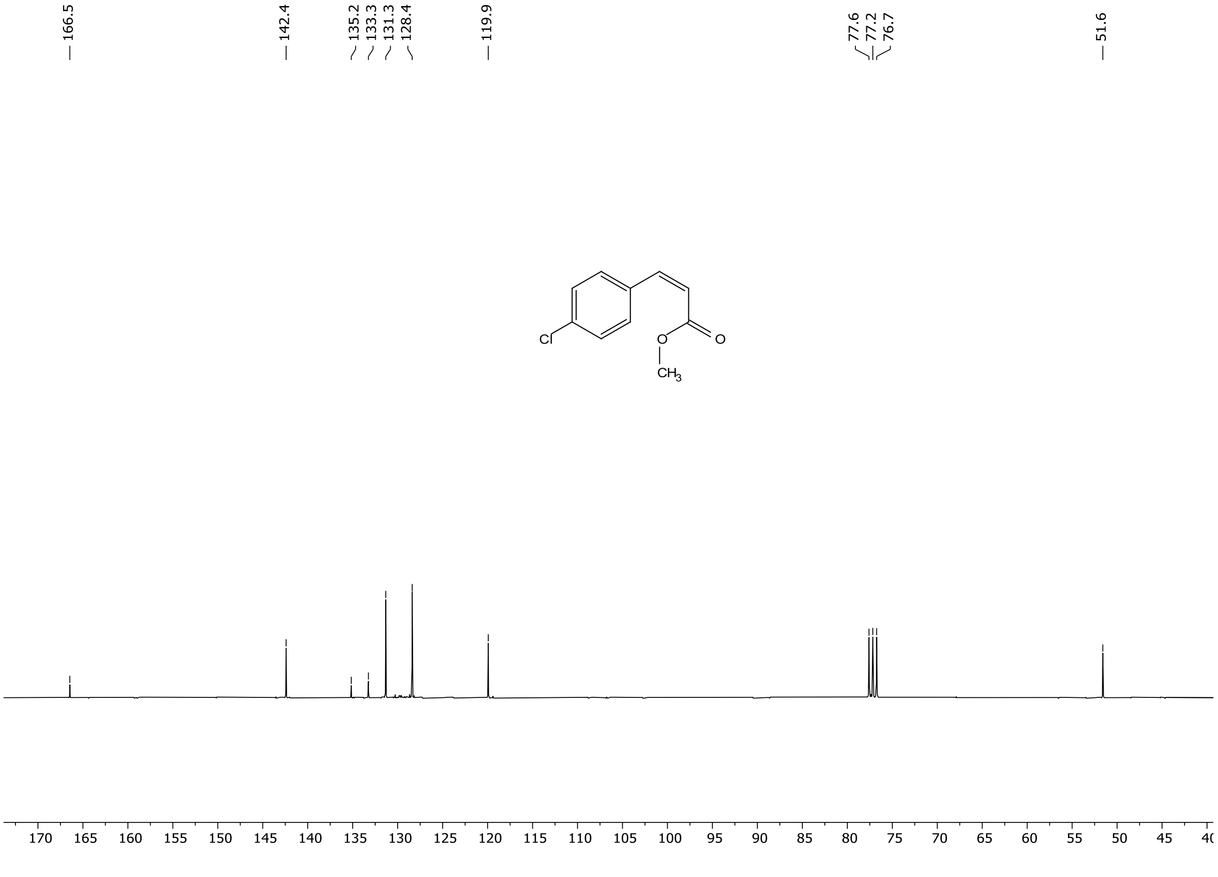
166.5
142.4
135.2
133.3
131.3
128.4
119.9
77.6
77.2
76.7
51.6
Cl
O
O
CH3

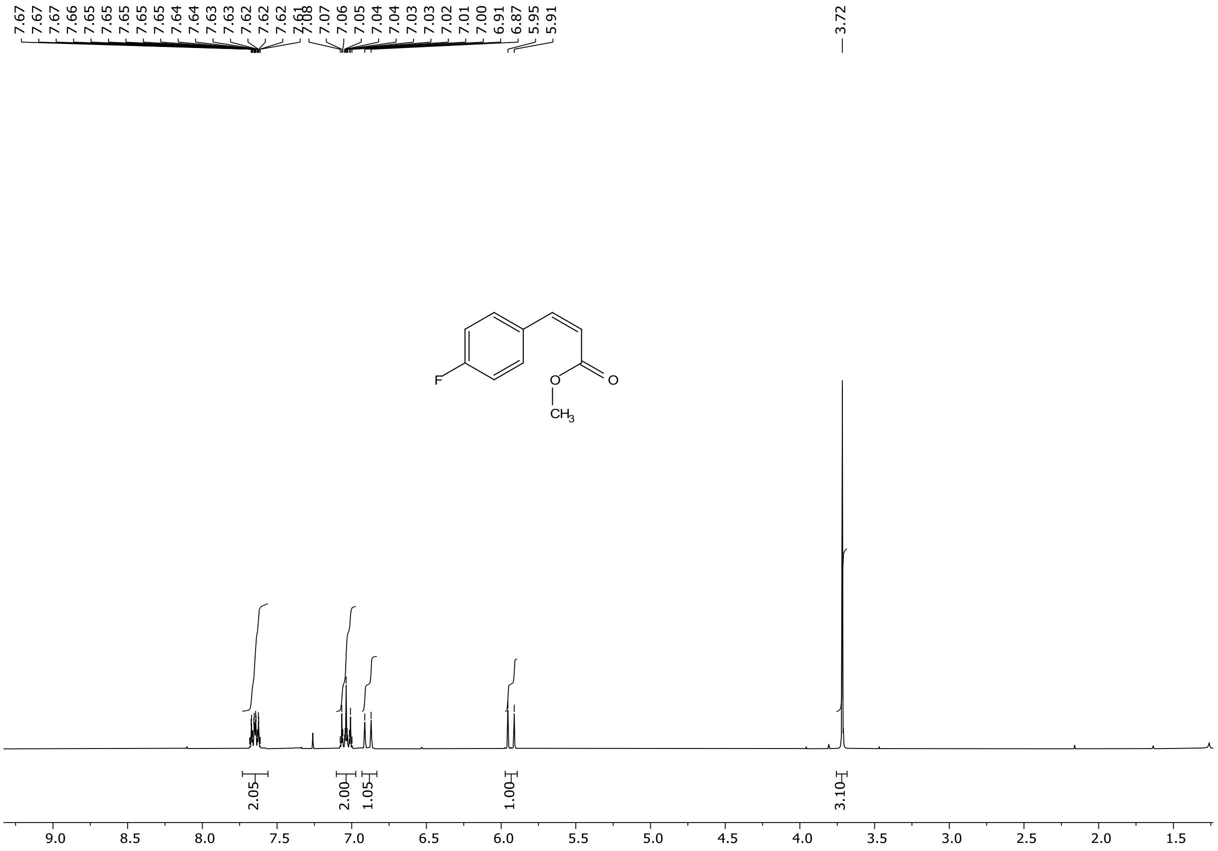
7.67
7.67
7.66
7.65
7.65
7.65
7.65
7.64
7.64
7.63
7.63
7.62
7.62
7.62
7.61
7.08
7.07
7.06
7.05
7.04
7.04
7.03
7.03
7.02
7.01
7.00
6.91
6.87
5.95
5.91
3.72
F
O
O
CH_3
2.05
2.00
1.05
1.00
3.10
9.0 8.5 8.0 7.5 7.0 6.5 6.0 5.5 5.0 4.5 4.0 3.5 3.0 2.5 2.0 1.5

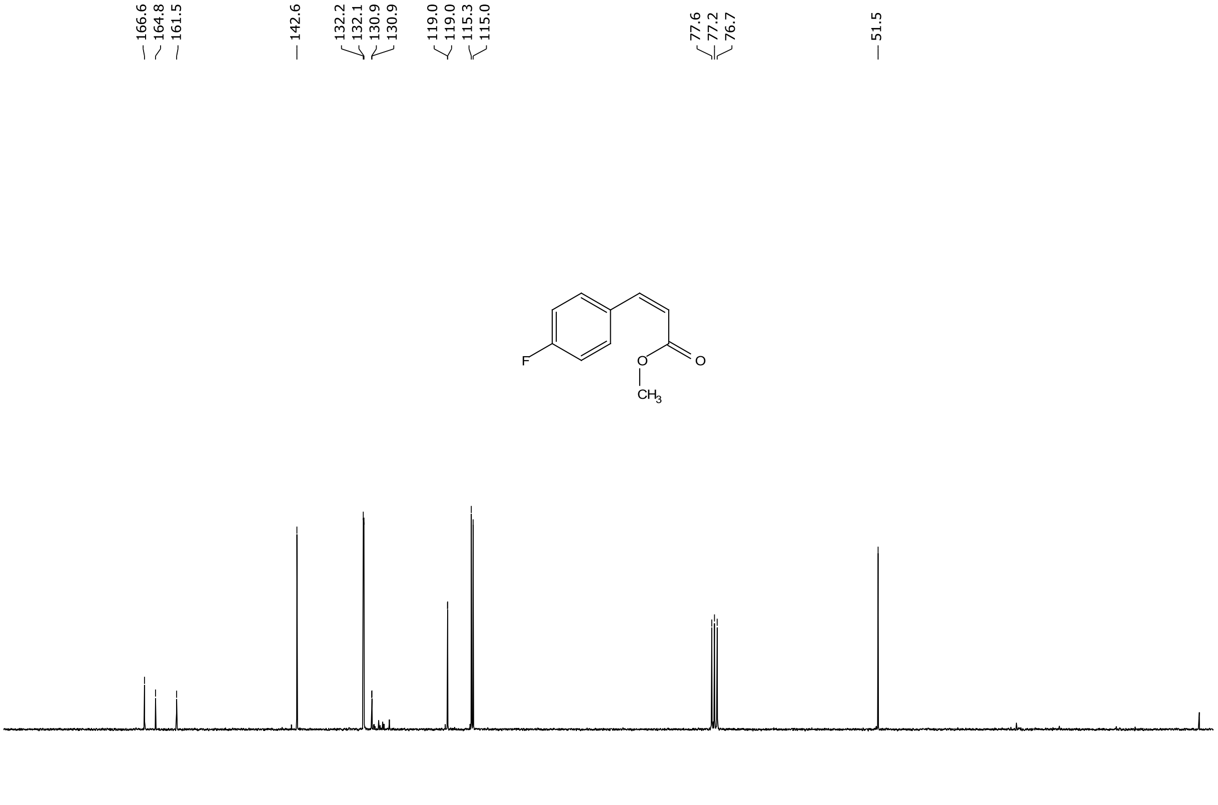
166.6
164.8
161.5
142.6
132.2
132.1
130.9
130.9
119.0
119.0
115.3
115.0
77.6
77.2
76.7
51.5
F
O
O
CH_3
180 170 160 150 140 130 120 110 100 90 80 70 60 50 40 30 20 10 0

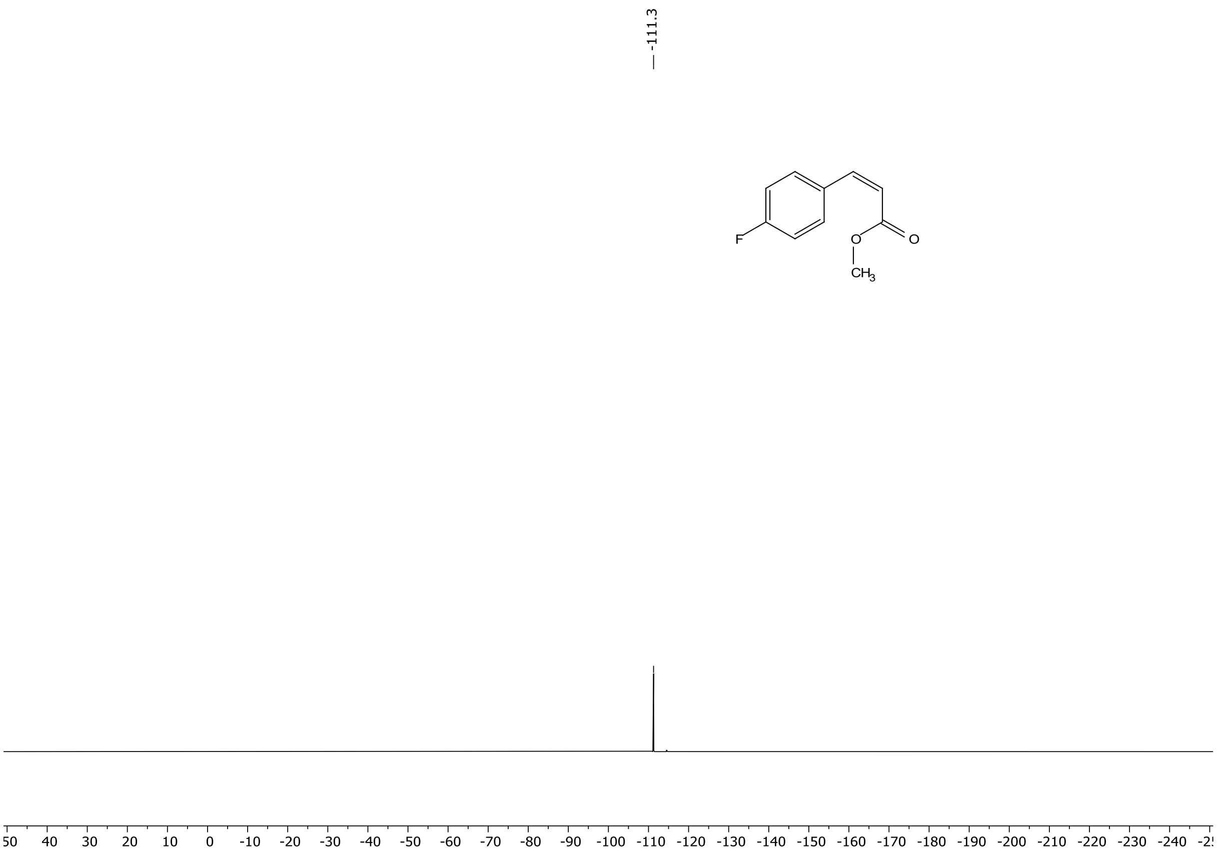
-111.3
F
O
O
CH3
50 40 30 20 10 0 -10 -20 -30 -40 -50 -60 -70 -80 -90 -100 -110 -120 -130 -140 -150 -160 -170 -180 -190 -200 -210 -220 -230 -240

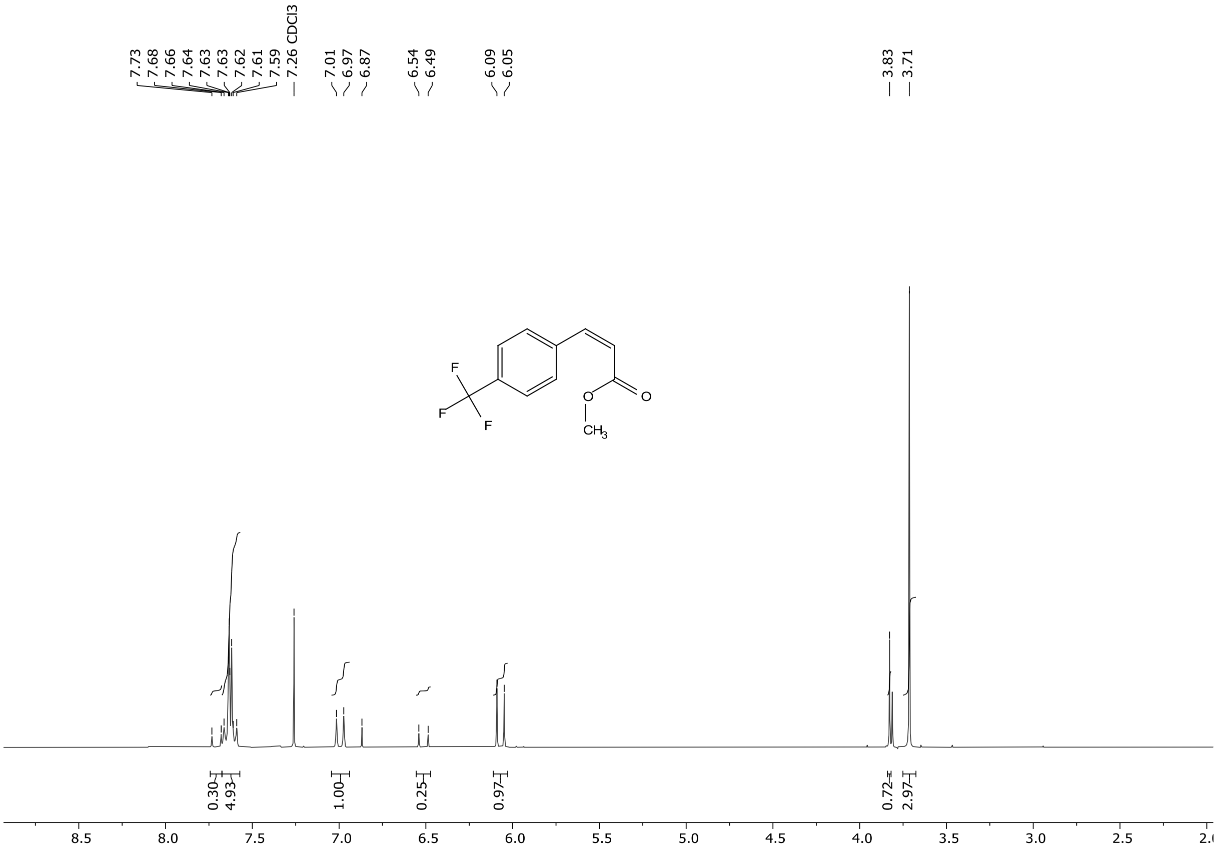
7.73
7.68
7.66
7.64
7.63
7.63
7.62
7.61
7.59
7.26 CDCl3
7.01
6.97
6.87
6.54
6.49
6.09
6.05
3.83
3.71
F
F
F
O
O
CH3
0.30
4.93
1.00
0.25
0.97
0.72
2.97
8.5
8.0
7.5
7.0
6.5
6.0
5.5
5.0
4.5
4.0
3.5
3.0
2.5

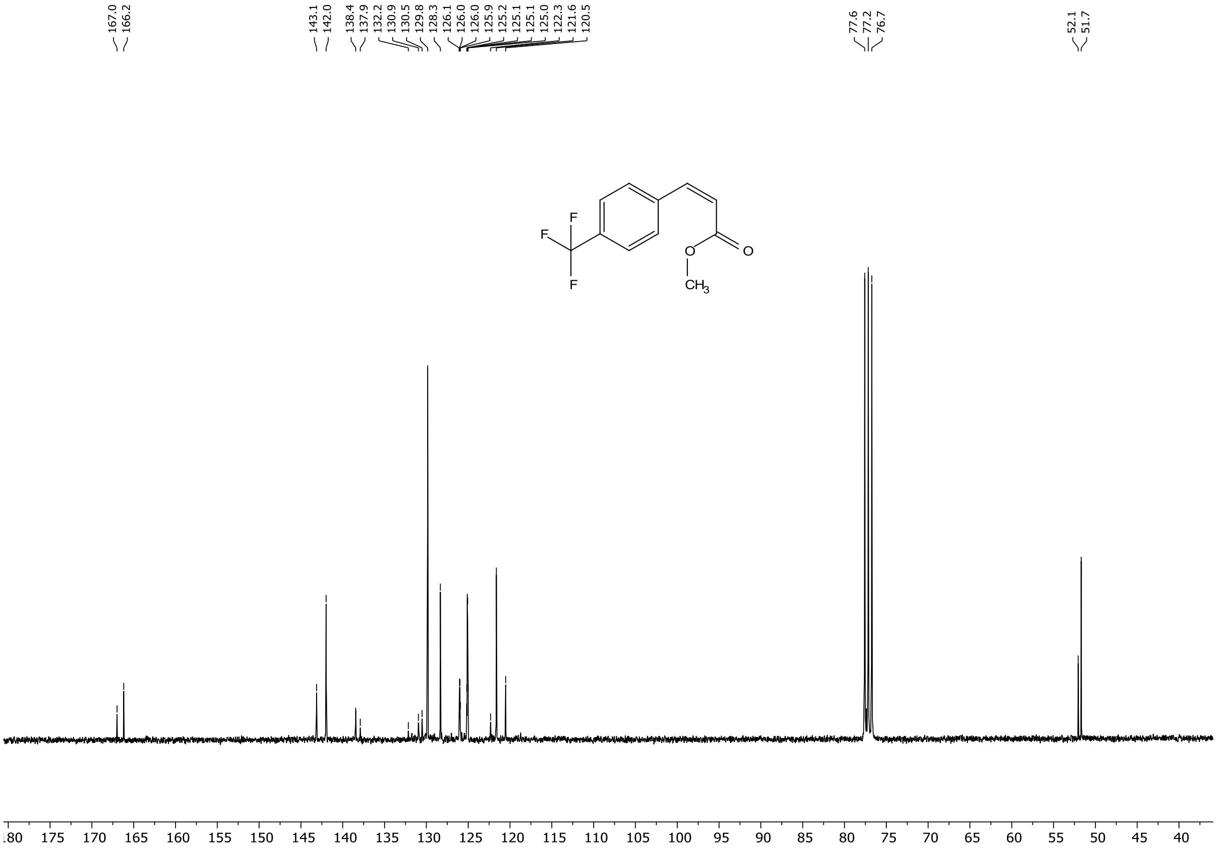
167.0
166.2
143.1
142.0
138.4
137.9
132.2
130.9
130.5
129.8
128.3
126.1
126.0
126.0
125.9
125.2
125.1
125.1
125.0
122.3
121.6
120.5
77.6
77.2
76.7
52.1
51.7
F
F
F
O
O
CH3
175
170
165
160
155
150
145
140
135
130
125
120
115
110
105
100
95
90
85
80
75
70
65
60
55
50
45
40

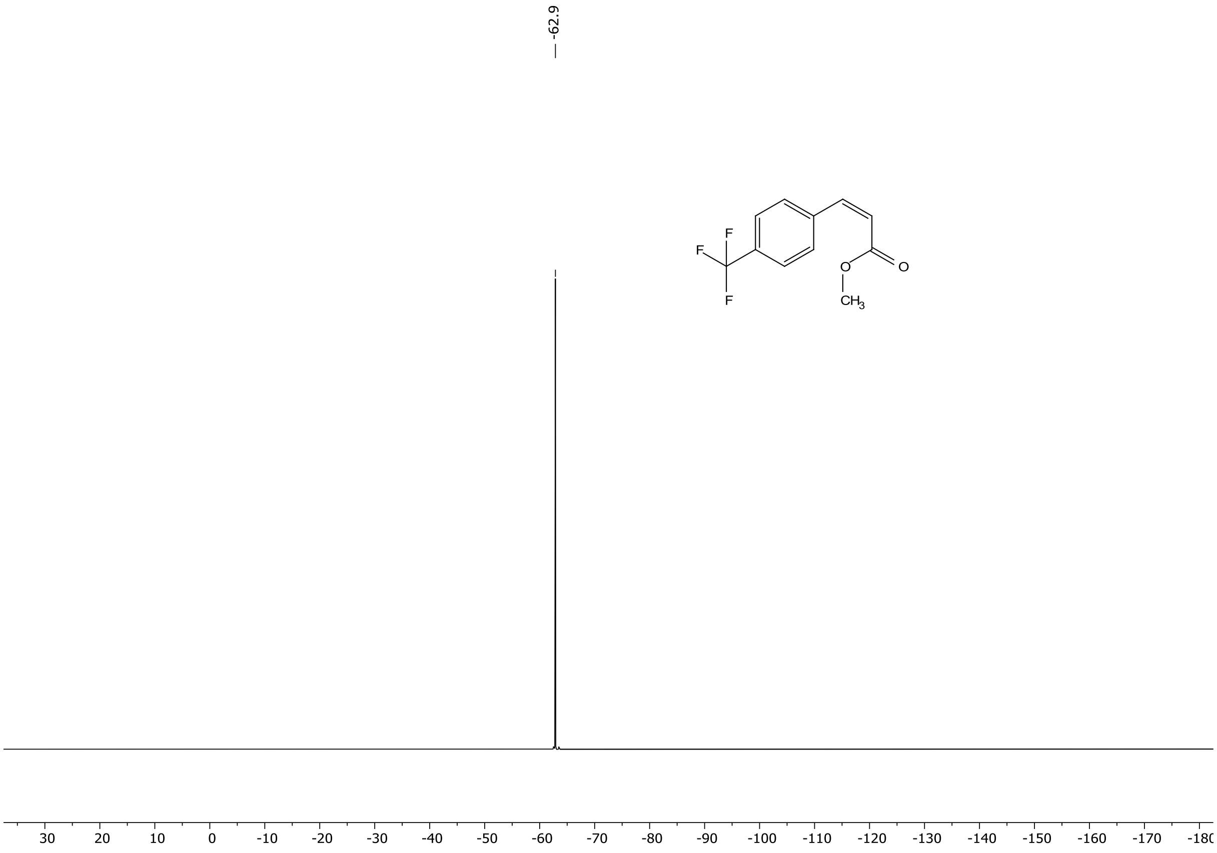
-62.9
F
F
F
O
O
CH3
30 20 10 0 -10 -20 -30 -40 -50 -60 -70 -80 -90 -100 -110 -120 -130 -140 -150 -160 -170 -180

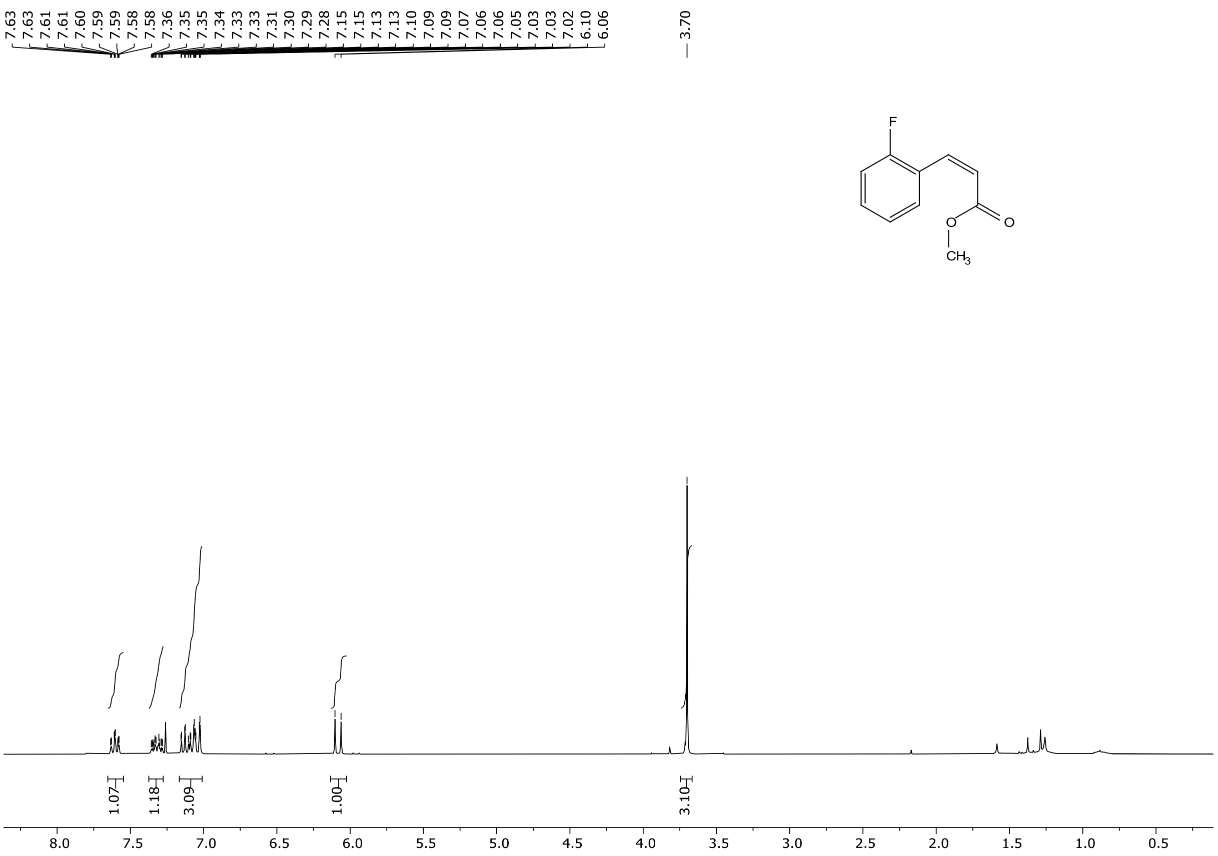
7.63 7.63 7.61 7.60 7.59 7.59 7.58 7.58 7.36 7.35 7.35 7.34 7.33 7.33 7.31 7.30 7.29 7.28 7.15 7.15 7.13 7.13 7.10 7.09 7.09 7.07 7.06 7.06 7.05 7.03 7.03 7.02 6.10 6.06
3.70
F
O
O
CH3
1.07
1.18
3.09
1.00
3.10
8.0 7.5 7.0 6.5 6.0 5.5 5.0 4.5 4.0 3.5 3.0 2.5 2.0 1.5 1.0 0.5

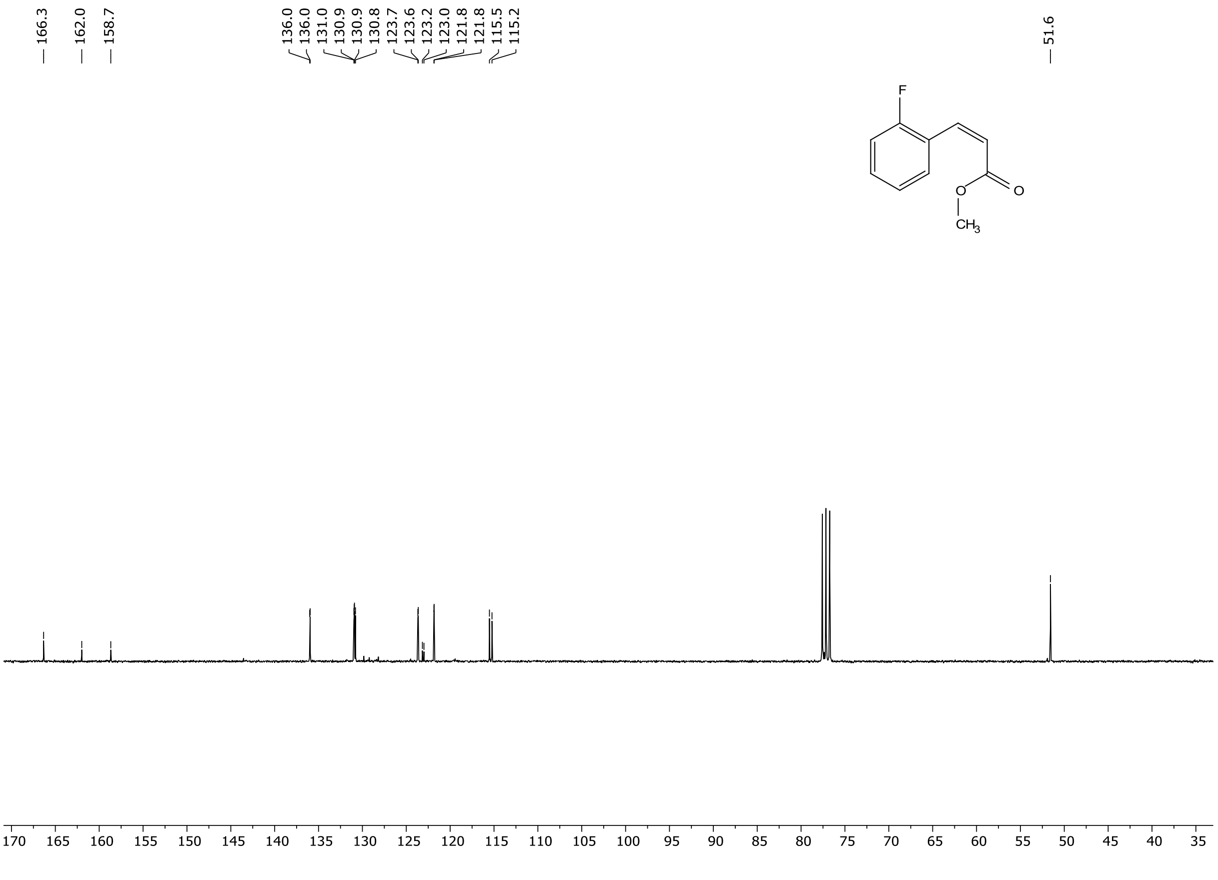
166.3
162.0
158.7
136.0 136.0 131.0 130.9 130.9 130.8 123.7 123.6 123.2 123.0 121.8 121.8 115.5 115.2
51.6
F
O
O
CH3
170 165 160 155 150 145 140 135 130 125 120 115 110 105 100 95 90 85 80 75 70 65 60 55 50 45 40 35

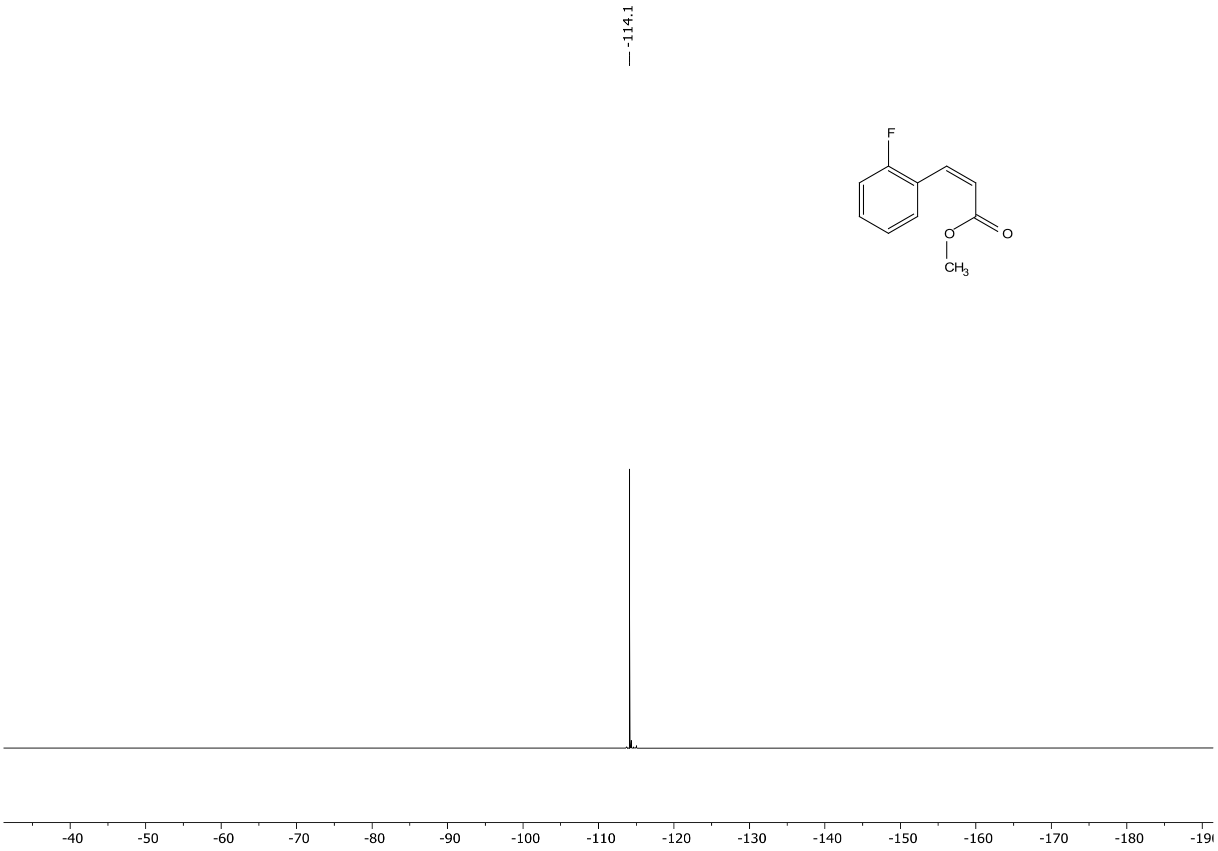

-114.1
F
O
O
CH3
-40
-50
-60
-70
-80
-90
-100
-110
-120
-130
-140
-150
-160
-170
-180

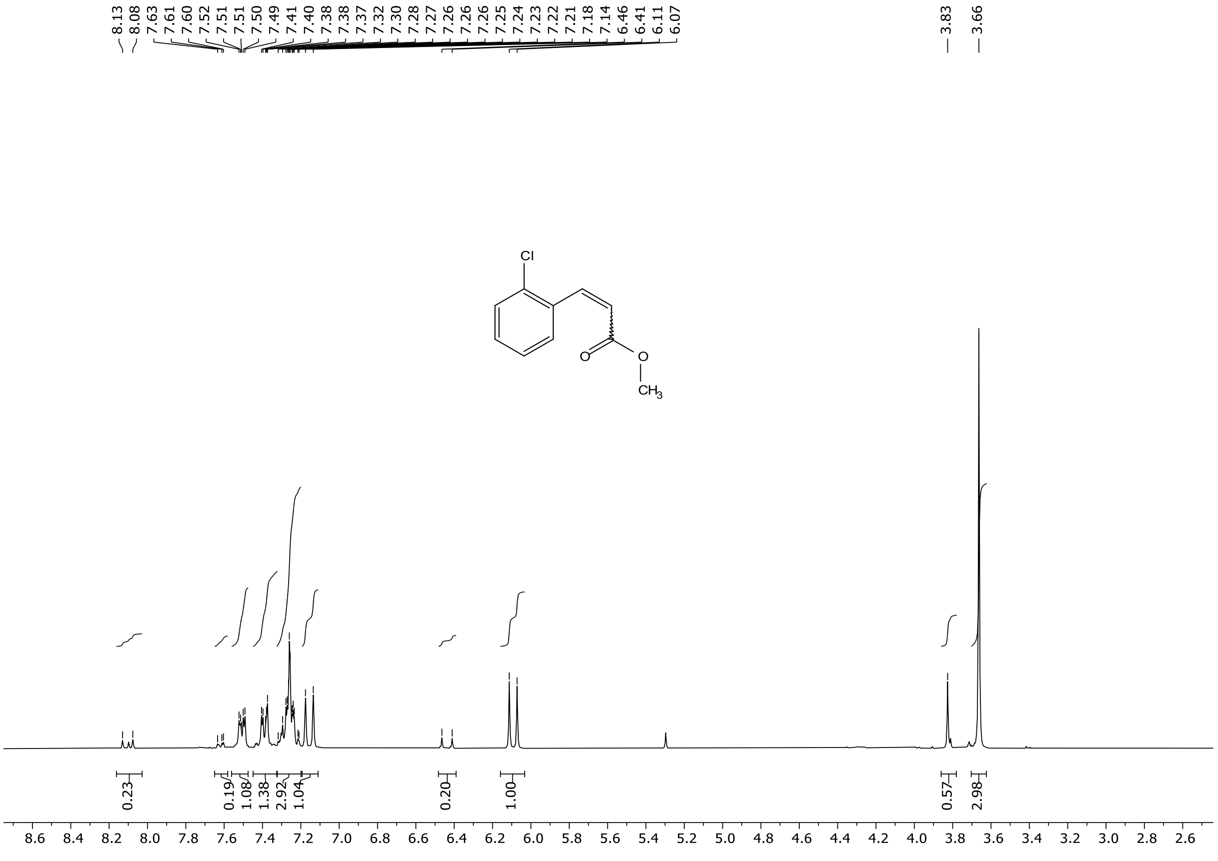
8.13
8.08
7.63
7.61
7.60
7.52
7.51
7.51
7.50
7.49
7.41
7.40
7.38
7.38
7.37
7.32
7.30
7.28
7.27
7.26
7.26
7.26
7.25
7.24
7.23
7.22
7.21
7.18
7.14
6.46
6.41
6.11
6.07
3.83
3.66
Cl
O
O
CH3
0.23
0.19
1.08
1.38
2.92
1.04
0.20
1.00
0.57
2.98

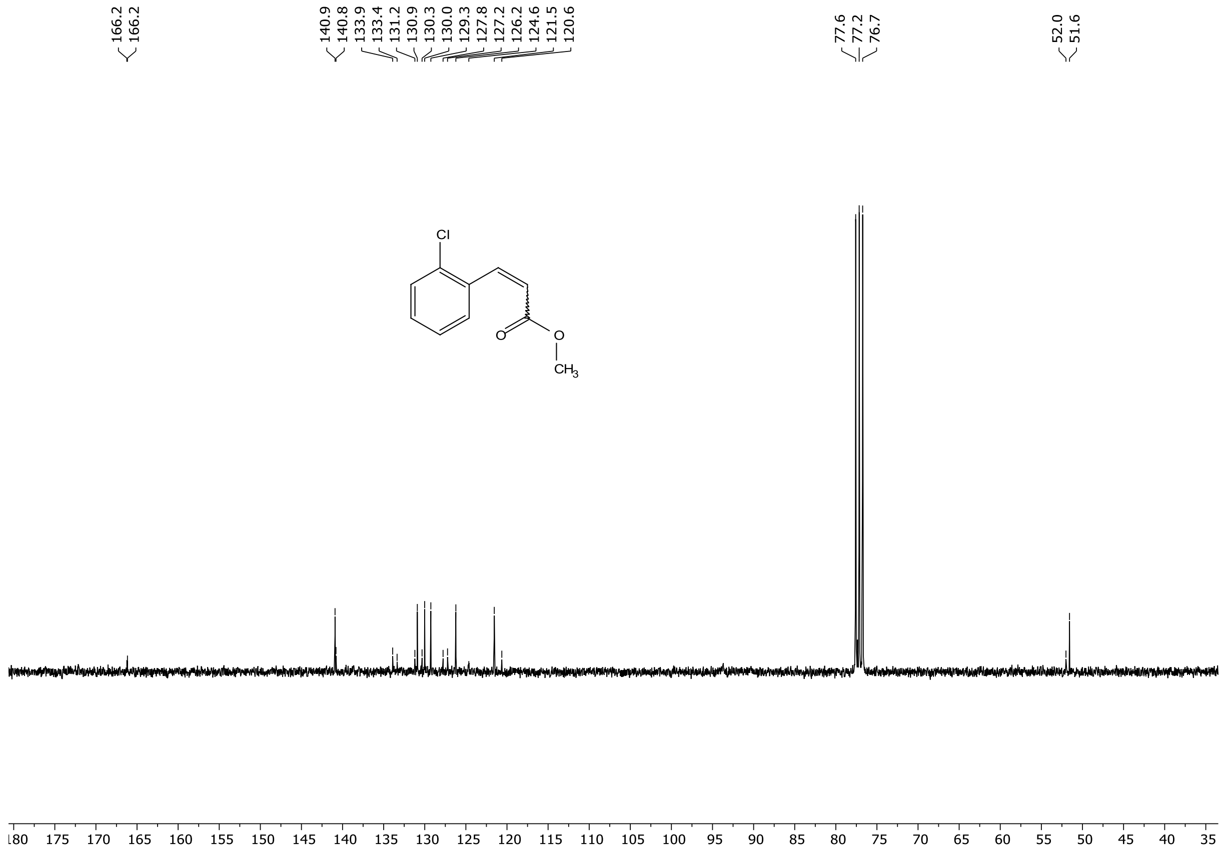
166.2
166.2
140.9
140.8
133.9
133.4
131.2
130.9
130.3
130.0
129.3
127.8
127.2
126.2
124.6
121.5
120.6
77.6
77.2
76.7
52.0
51.6
Cl
O
O
CH3

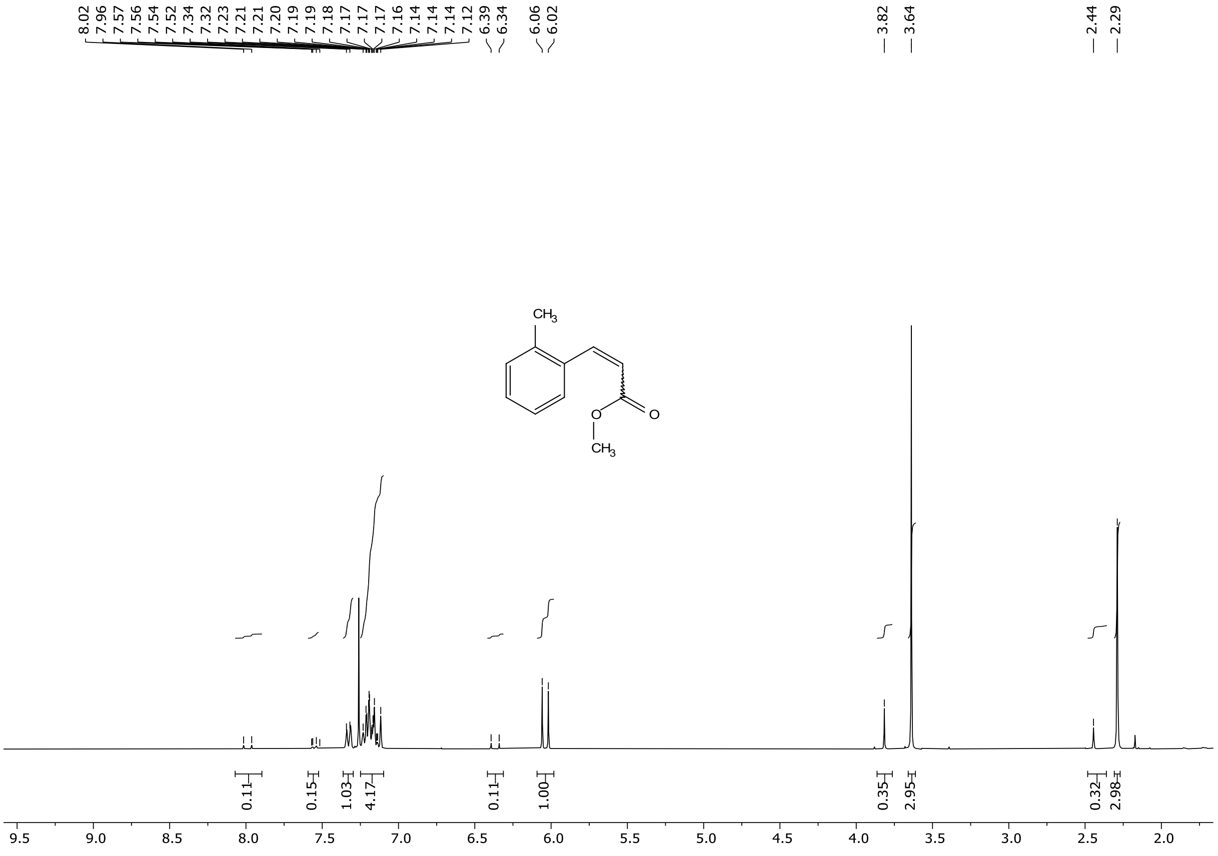
8.02 7.96 7.57 7.56 7.54 7.52 7.34 7.32 7.23 7.21 7.21 7.20 7.19 7.19 7.18 7.17 7.17 7.17 7.16 7.14 7.14 7.14 7.12 6.39 6.34 6.06 6.02
3.82 3.64
2.44 2.29
CH3
O
O
CH3
0.11 0.15 1.03 4.17 0.11 1.00 0.35 2.95 0.32 2.98
9.5 9.0 8.5 8.0 7.5 7.0 6.5 6.0 5.5 5.0 4.5 4.0 3.5 3.0 2.5 2.0

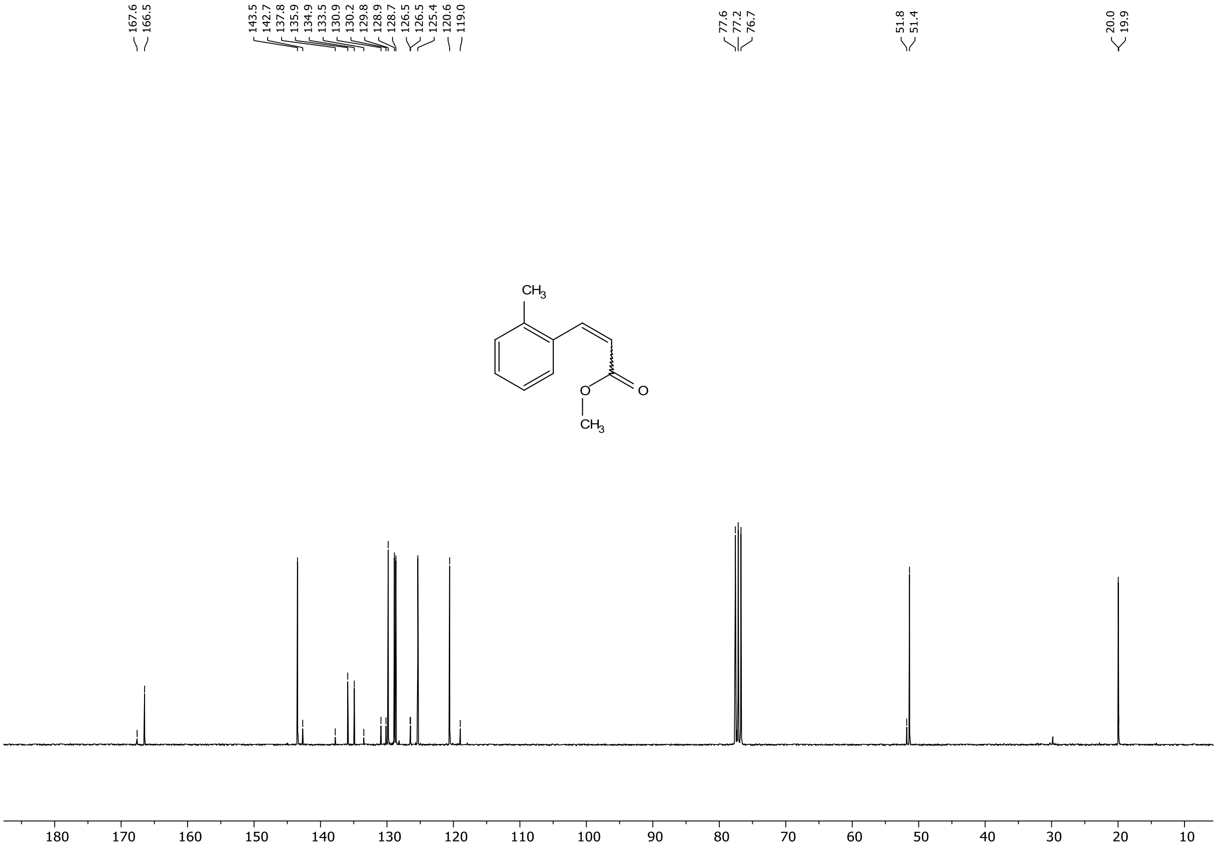
167.6 166.5
143.5 142.7 137.8 135.9 134.9 133.5 130.9 130.2 129.8 128.9 128.7 126.5 126.5 125.4 120.6 119.0
77.6 77.2 76.7
51.8 51.4
20.0 19.9
CH3
O
O
CH3
180 170 160 150 140 130 120 110 100 90 80 70 60 50 40 30 20 10

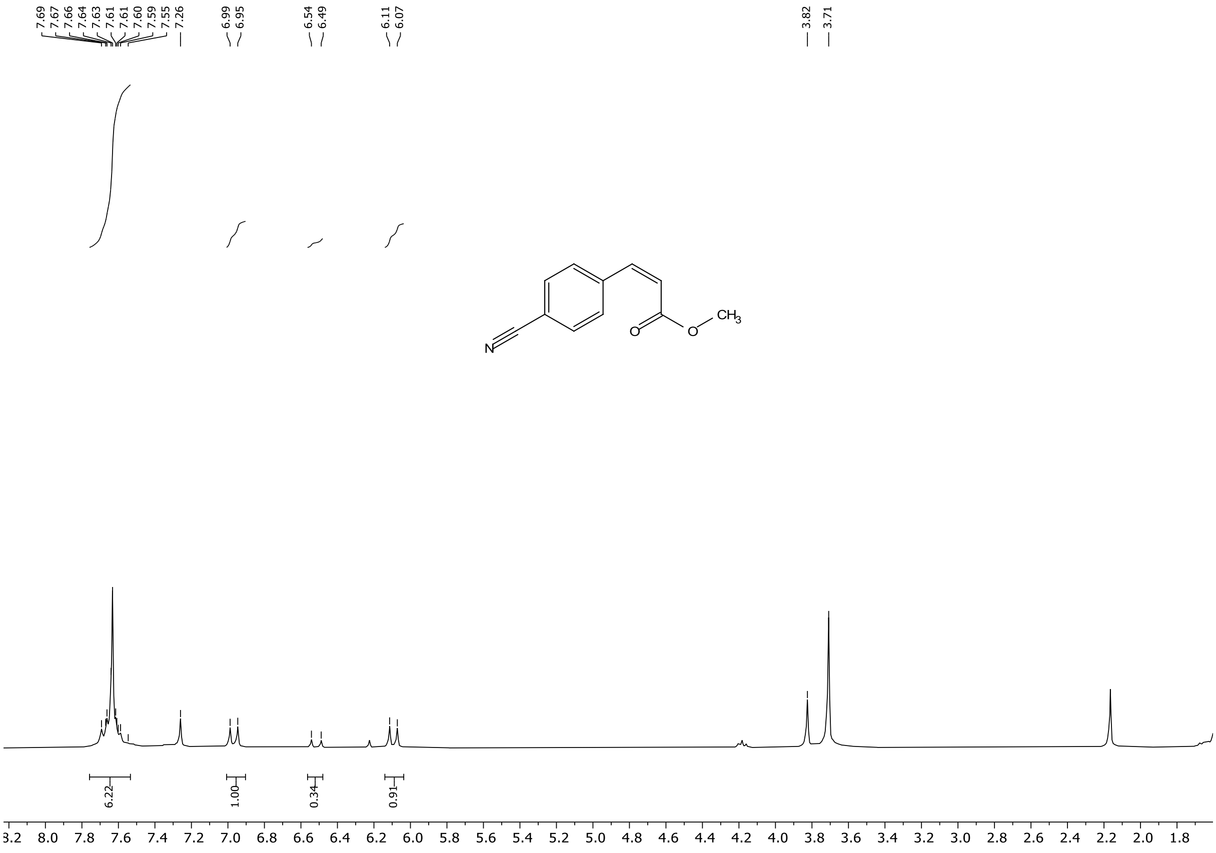
7.69
7.67
7.66
7.64
7.63
7.61
7.61
7.60
7.59
7.55
7.26
6.99
6.95
6.54
6.49
6.11
6.07
3.82
3.71
CH3
N
O
O
6.22
1.00
0.34
0.91
8.2 8.0 7.8 7.6 7.4 7.2 7.0 6.8 6.6 6.4 6.2 6.0 5.8 5.6 5.4 5.2 5.0 4.8 4.6 4.4 4.2 4.0 3.8 3.6 3.4 3.2 3.0 2.8 2.6 2.4 2.2 2.0 1.8

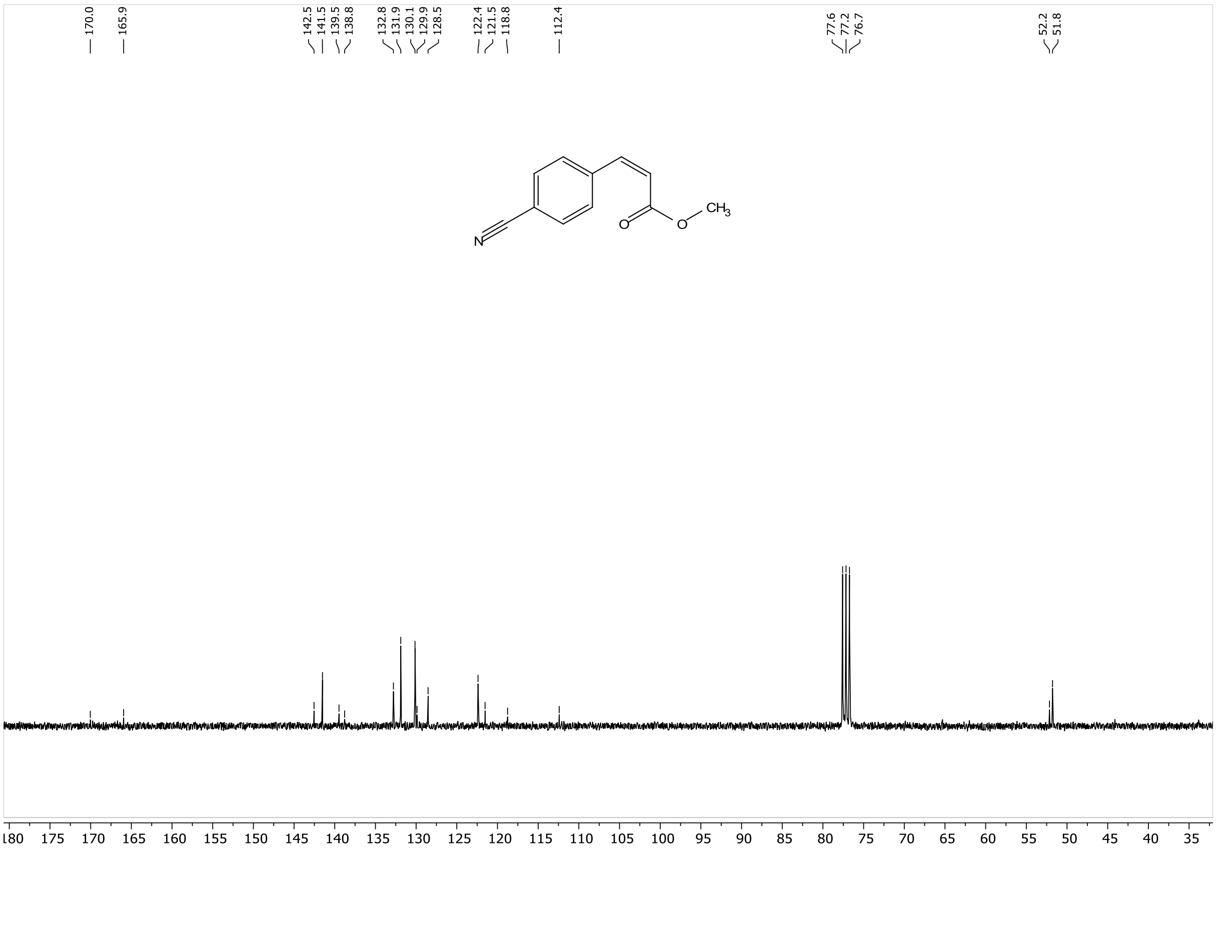
170.0
165.9
142.5
141.5
139.5
138.8
132.8
131.9
130.1
129.9
128.5
122.4
121.5
118.8
112.4
77.6
77.2
76.7
52.2
51.8
CH3
N
O
O
180 175 170 165 160 155 150 145 140 135 130 125 120 115 110 105 100 95 90 85 80 75 70 65 60 55 50 45 40 35

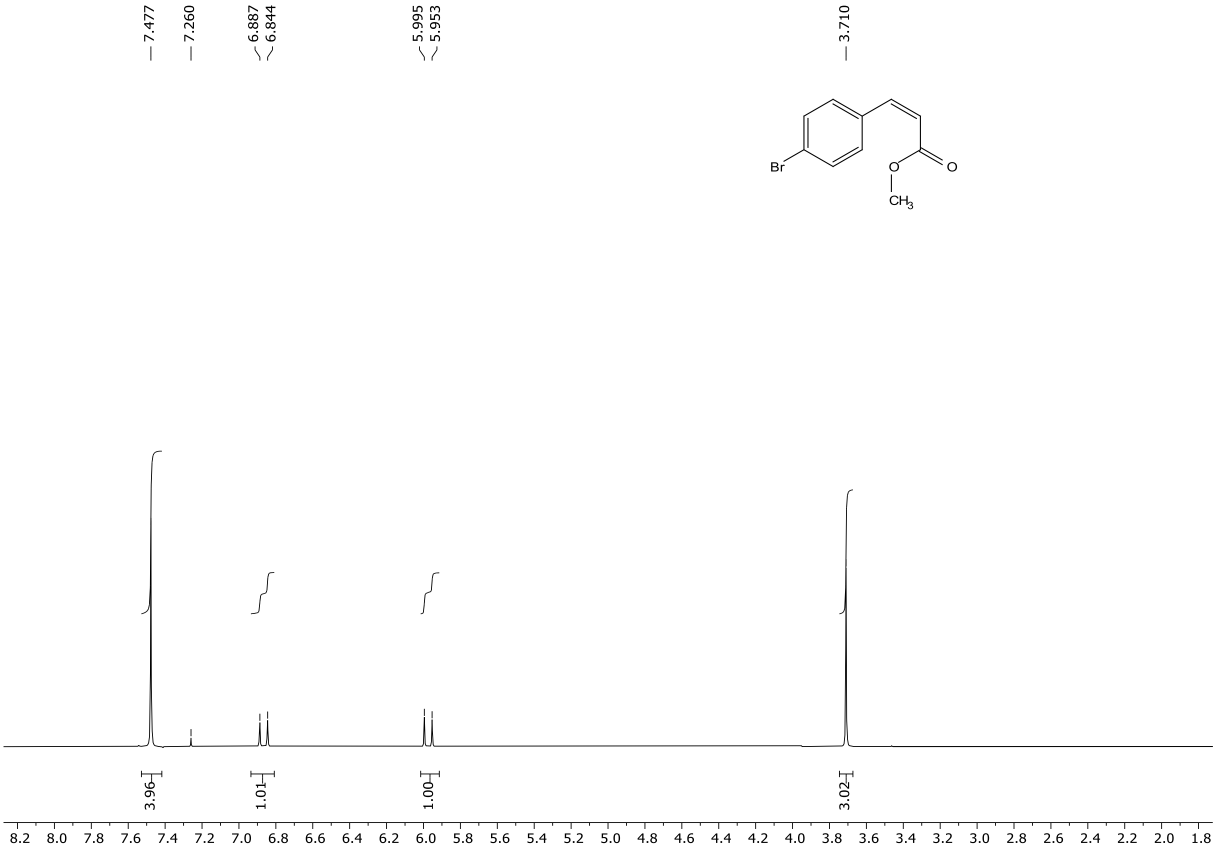

7.477
7.260
6.887
6.844
5.995
5.953
3.710
Br
O
O
CH3
3.96
1.01
1.00
3.02
8.2 8.0 7.8 7.6 7.4 7.2 7.0 6.8 6.6 6.4 6.2 6.0 5.8 5.6 5.4 5.2 5.0 4.8 4.6 4.4 4.2 4.0 3.8 3.6 3.4 3.2 3.0 2.8 2.6 2.4 2.2 2.0 1.8

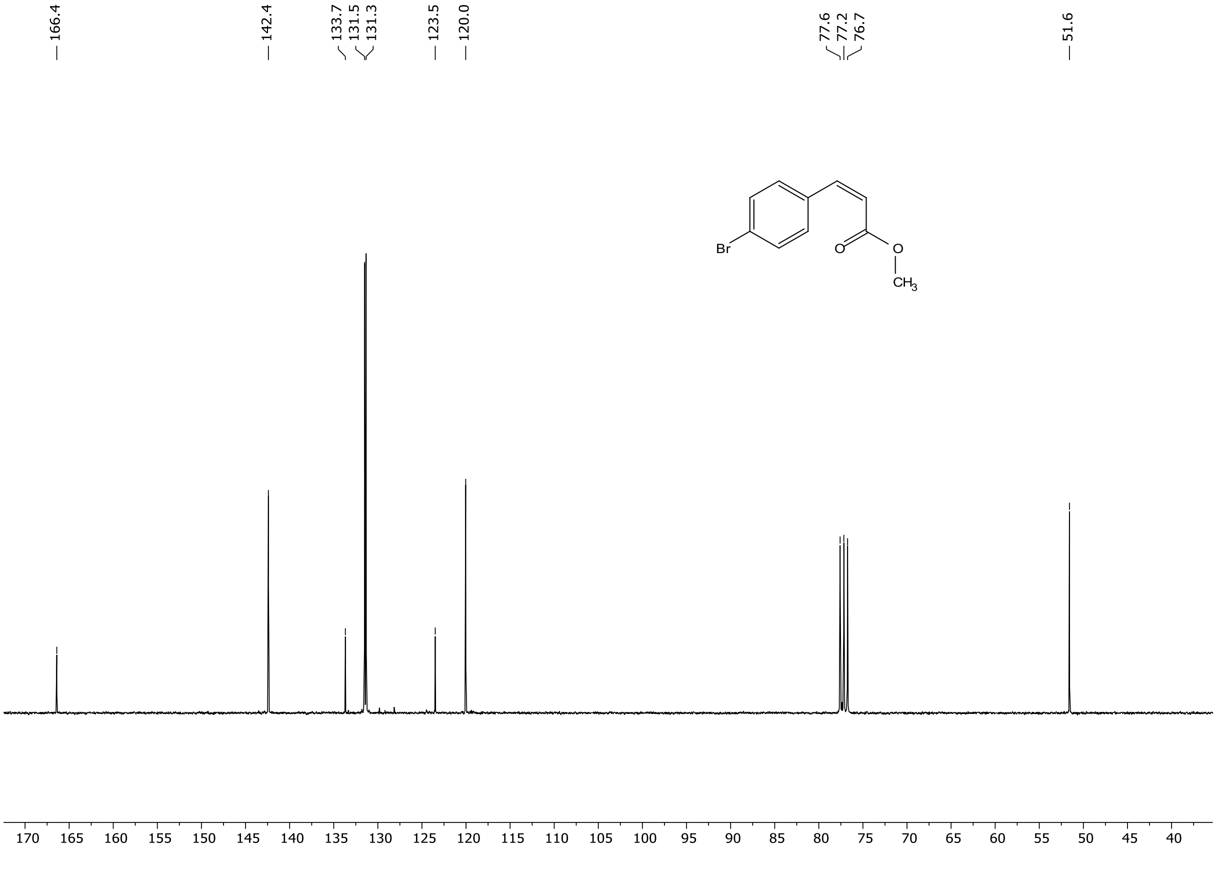

166.4
142.4
133.7
131.5
131.3
123.5
120.0
77.6
77.2
76.7
51.6
Br
O
O
CH3
170 165 160 155 150 145 140 135 130 125 120 115 110 105 100 95 90 85 80 75 70 65 60 55 50 45 40

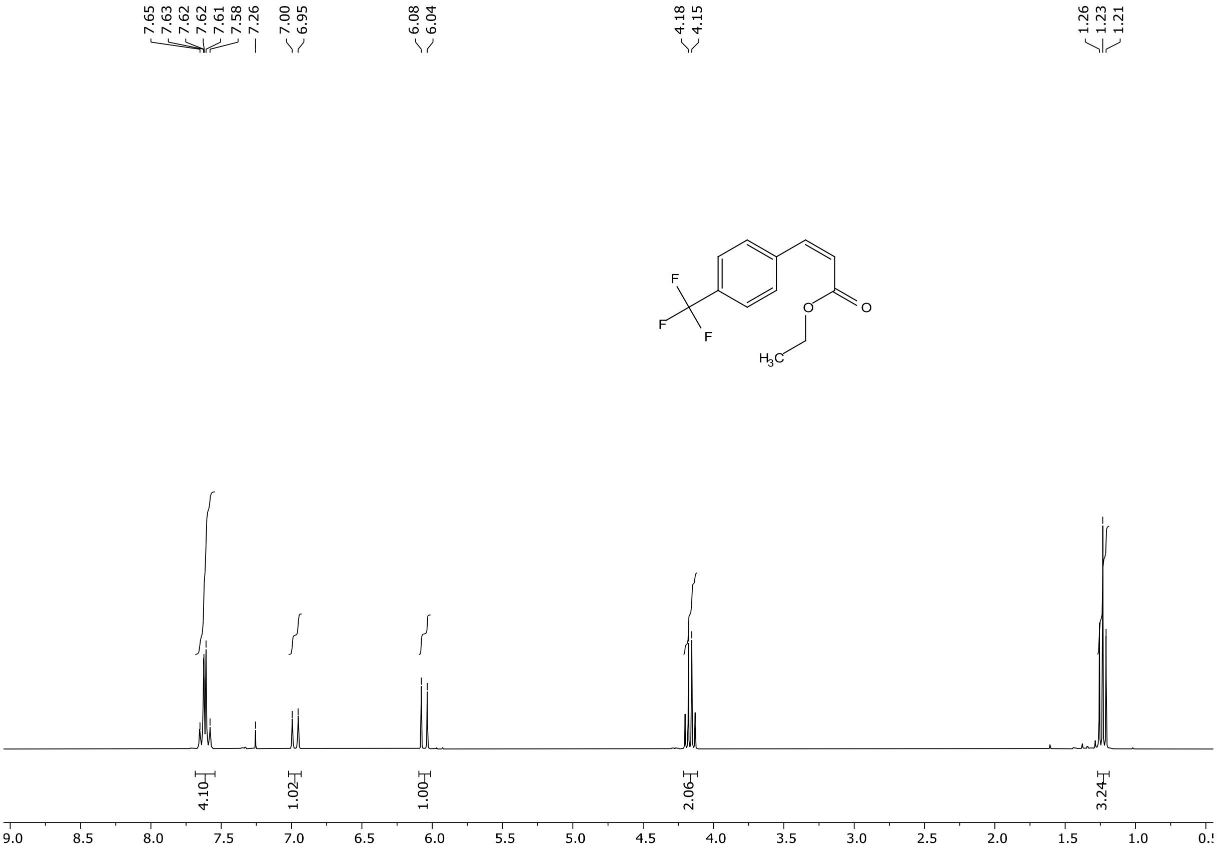
7.65
7.63
7.62
7.62
7.61
7.58
7.26
7.00
6.95
6.08
6.04
4.18
4.15
1.26
1.23
1.21
F
F
F
O
O
H3C
4.10
1.02
1.00
2.06
3.24
9.0
8.5
8.0
7.5
7.0
6.5
6.0
5.5
5.0
4.5
4.0
3.5
3.0
2.5
2.0
1.5
1.0

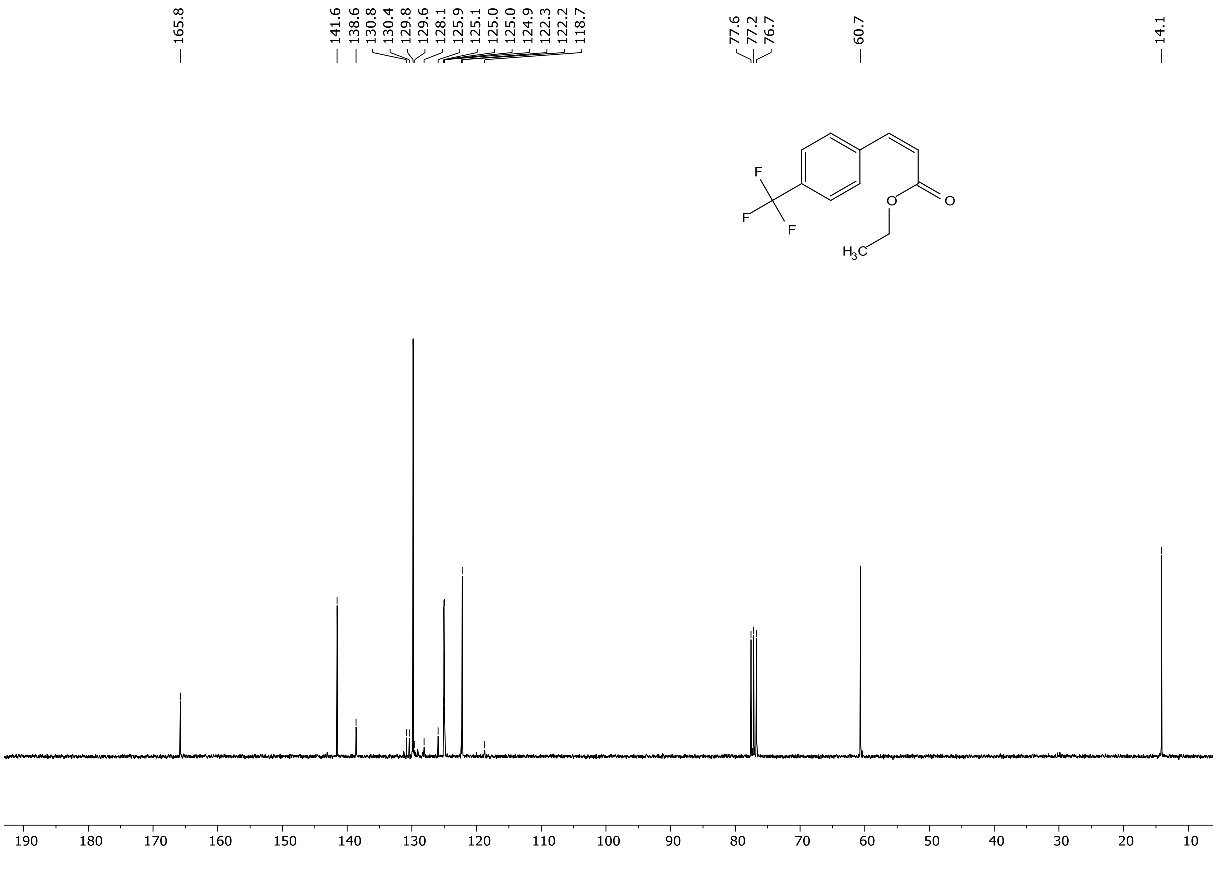
165.8
141.6
138.6
130.8
130.4
129.8
129.6
128.1
125.9
125.1
125.0
125.0
124.9
122.3
122.2
118.7
77.6
77.2
76.7
60.7
14.1
F
F
F
O
O
H3C
190
180
170
160
150
140
130
120
110
100
90
80
70
60
50
40
30
20
10

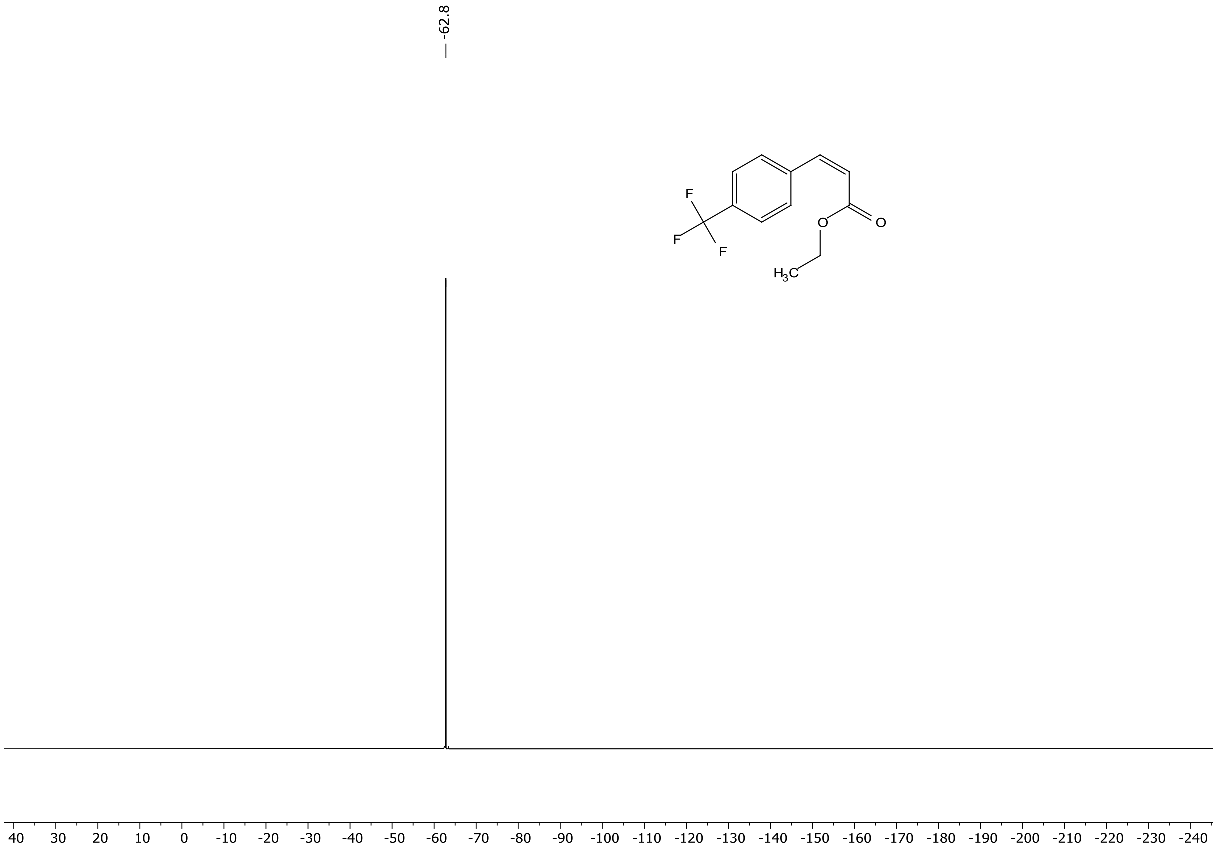
-62.8
F
F
F
O
O
H3C
40 30 20 10 0 -10 -20 -30 -40 -50 -60 -70 -80 -90 -100 -110 -120 -130 -140 -150 -160 -170 -180 -190 -200 -210 -220 -230 -240

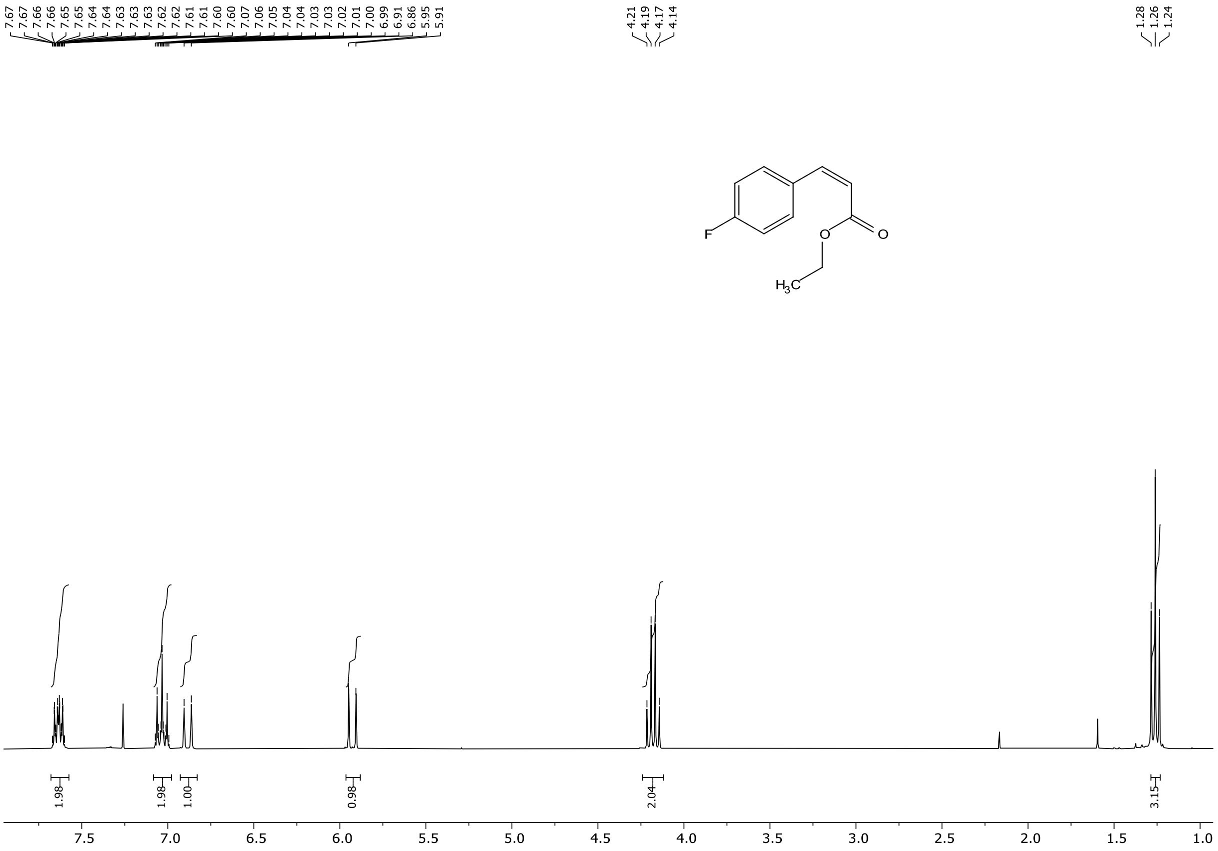
7.67
7.67
7.66
7.66
7.65
7.65
7.64
7.64
7.63
7.63
7.63
7.62
7.62
7.61
7.61
7.60
7.60
7.07
7.06
7.05
7.04
7.04
7.03
7.03
7.02
7.01
7.00
6.99
6.91
6.86
5.95
5.91
4.21
4.19
4.17
4.14
1.28
1.26
1.24
F
O
O
H3C
1.98
1.98
1.00
0.98
2.04
3.15
7.5
7.0
6.5
6.0
5.5
5.0
4.5
4.0
3.5
3.0
2.5
2.0
1.5
1.0

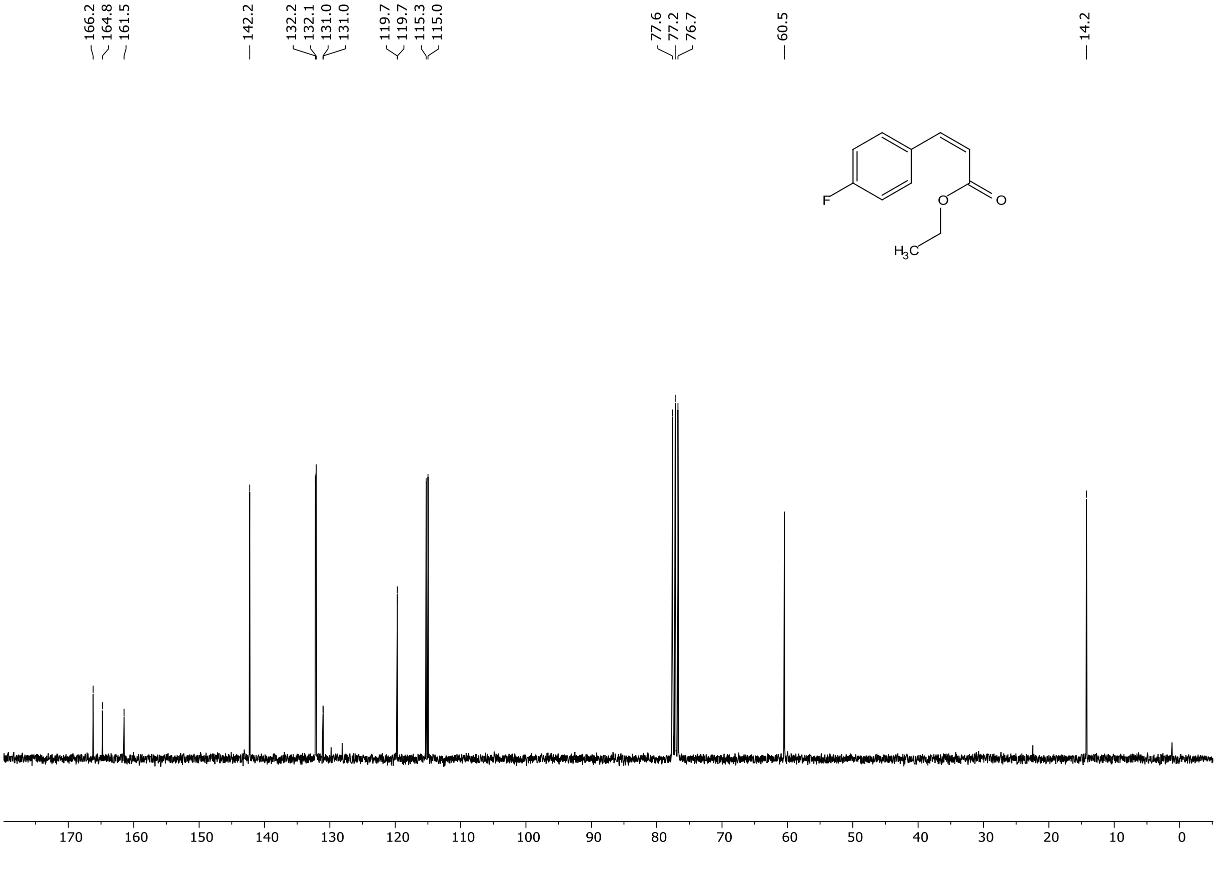
166.2
164.8
161.5
142.2
132.2
132.1
131.0
131.0
119.7
119.7
115.3
115.0
77.6
77.2
76.7
60.5
14.2
F
O
O
H3C
170
160
150
140
130
120
110
100
90
80
70
60
50
40
30
20
10
0

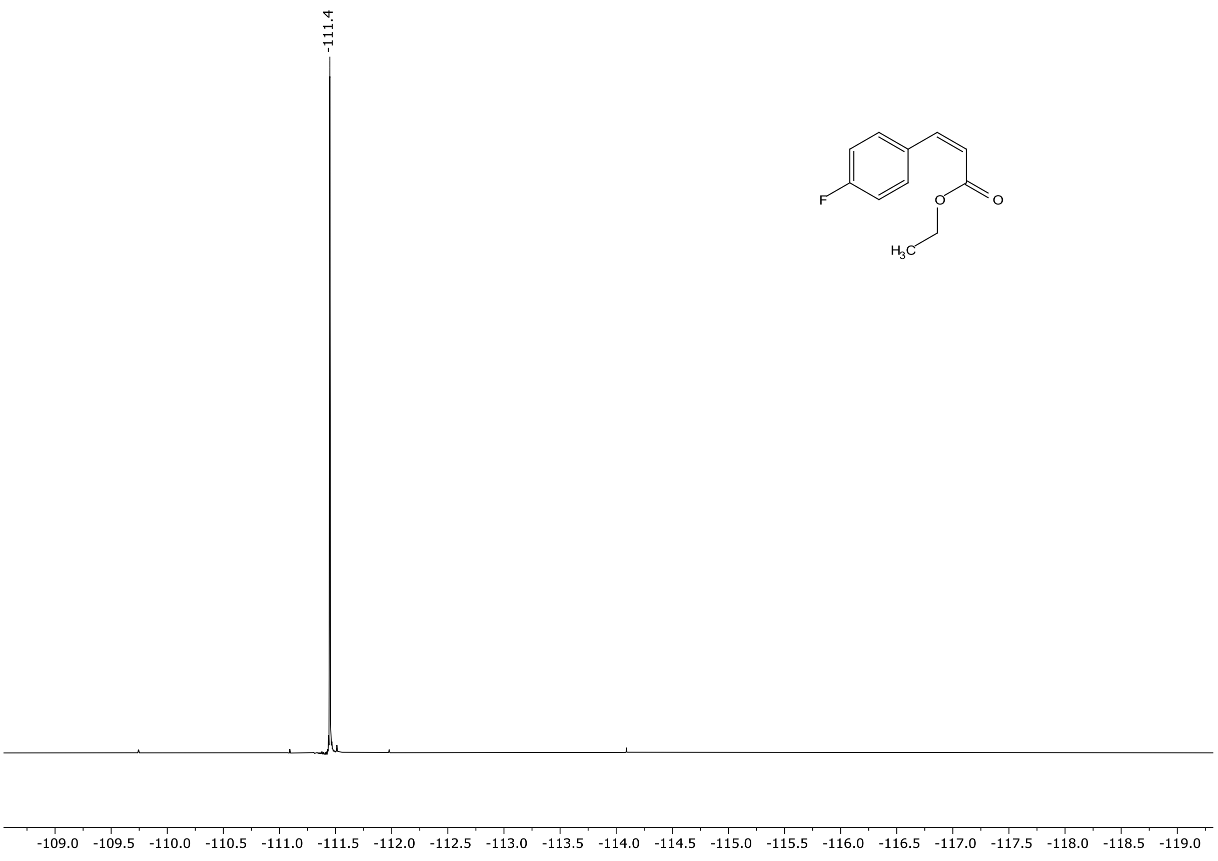
F
O
O
H3C
-111.4
-109.0 -109.5 -110.0 -110.5 -111.0 -111.5 -112.0 -112.5 -113.0 -113.5 -114.0 -114.5 -115.0 -115.5 -116.0 -116.5 -117.0 -117.5 -118.0 -118.5 -119.0

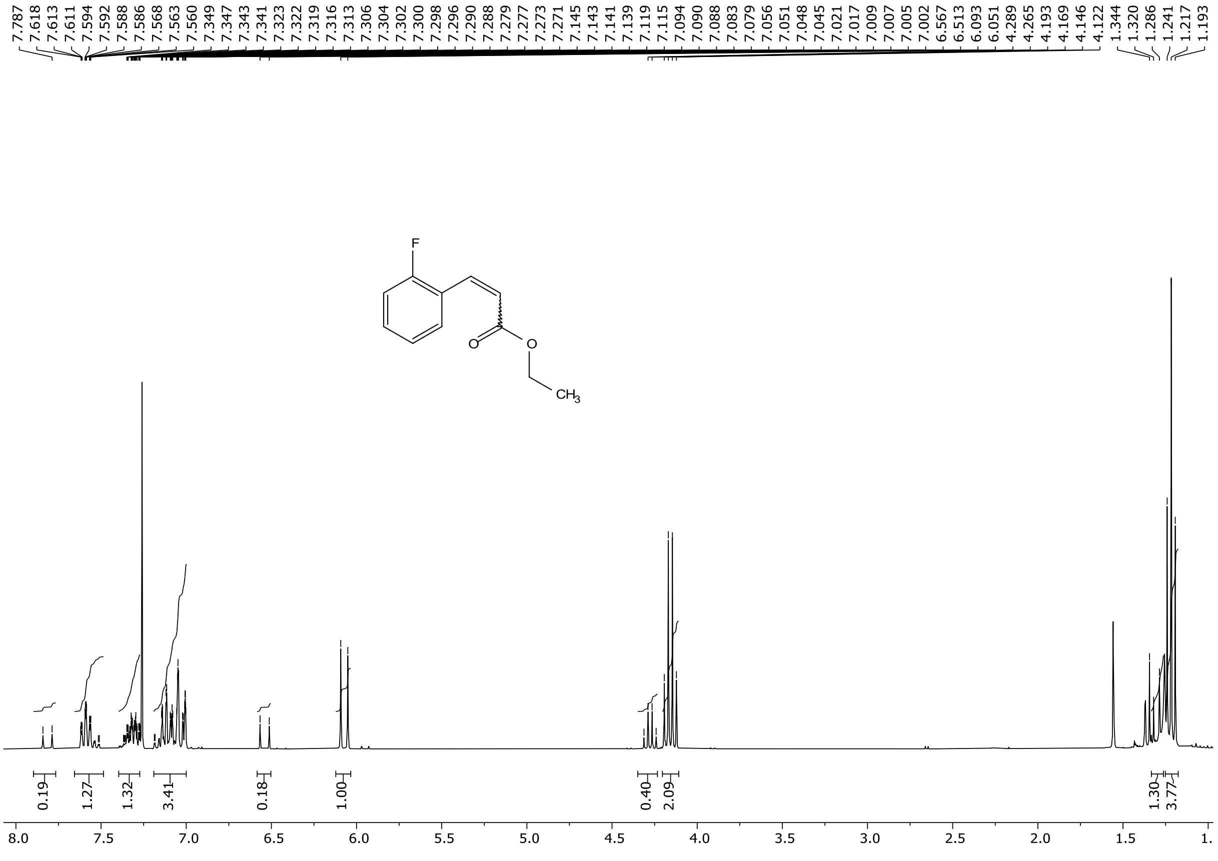
7.787
7.618
7.613
7.611
7.594
7.592
7.588
7.586
7.568
7.563
7.560
7.349
7.347
7.343
7.341
7.323
7.322
7.319
7.316
7.313
7.306
7.304
7.302
7.300
7.298
7.296
7.290
7.288
7.279
7.277
7.273
7.271
7.145
7.143
7.141
7.139
7.119
7.115
7.094
7.090
7.088
7.083
7.079
7.056
7.051
7.048
7.045
7.021
7.017
7.009
7.007
7.005
7.002
6.567
6.513
6.093
6.051
4.289
4.265
4.193
4.169
4.146
4.122
1.344
1.320
1.286
1.241
1.217
1.193
F
O
O
CH3
0.19
1.27
1.32
3.41
0.18
1.00
0.40
2.09
1.30
3.77
8.0 7.5 7.0 6.5 6.0 5.5 5.0 4.5 4.0 3.5 3.0 2.5 2.0 1.5 1.

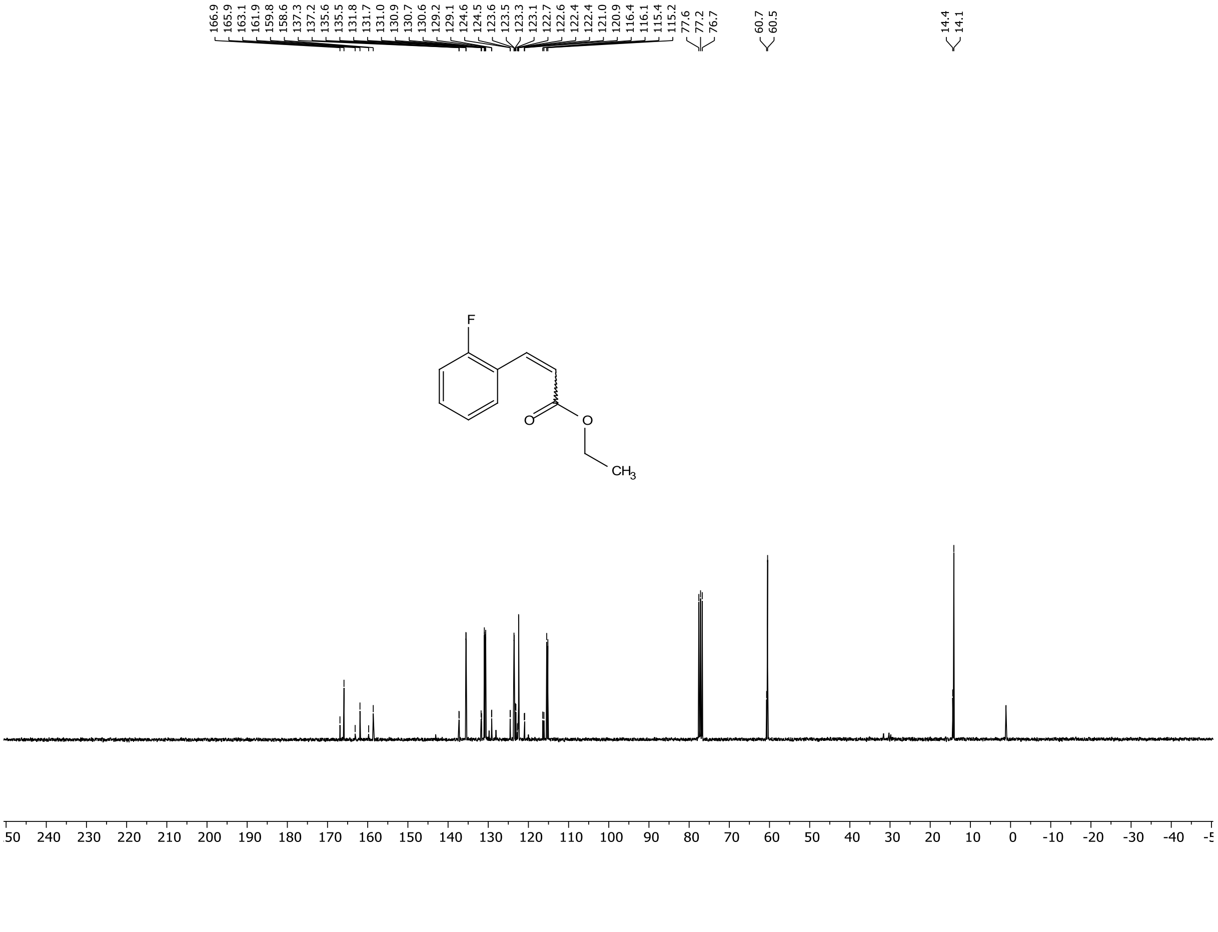
166.9
165.9
163.1
161.9
159.8
158.6
137.3
137.2
135.6
135.5
131.8
131.7
131.0
130.9
130.7
130.6
129.2
129.1
124.6
124.5
123.6
123.5
123.3
123.1
122.7
122.6
122.4
122.4
121.0
120.9
116.4
116.1
115.4
115.2
77.6
77.2
76.7
60.7
60.5
14.4
14.1
F
O
O
CH3
50 240 230 220 210 200 190 180 170 160 150 140 130 120 110 100 90 80 70 60 50 40 30 20 10 0 -10 -20 -30 -40

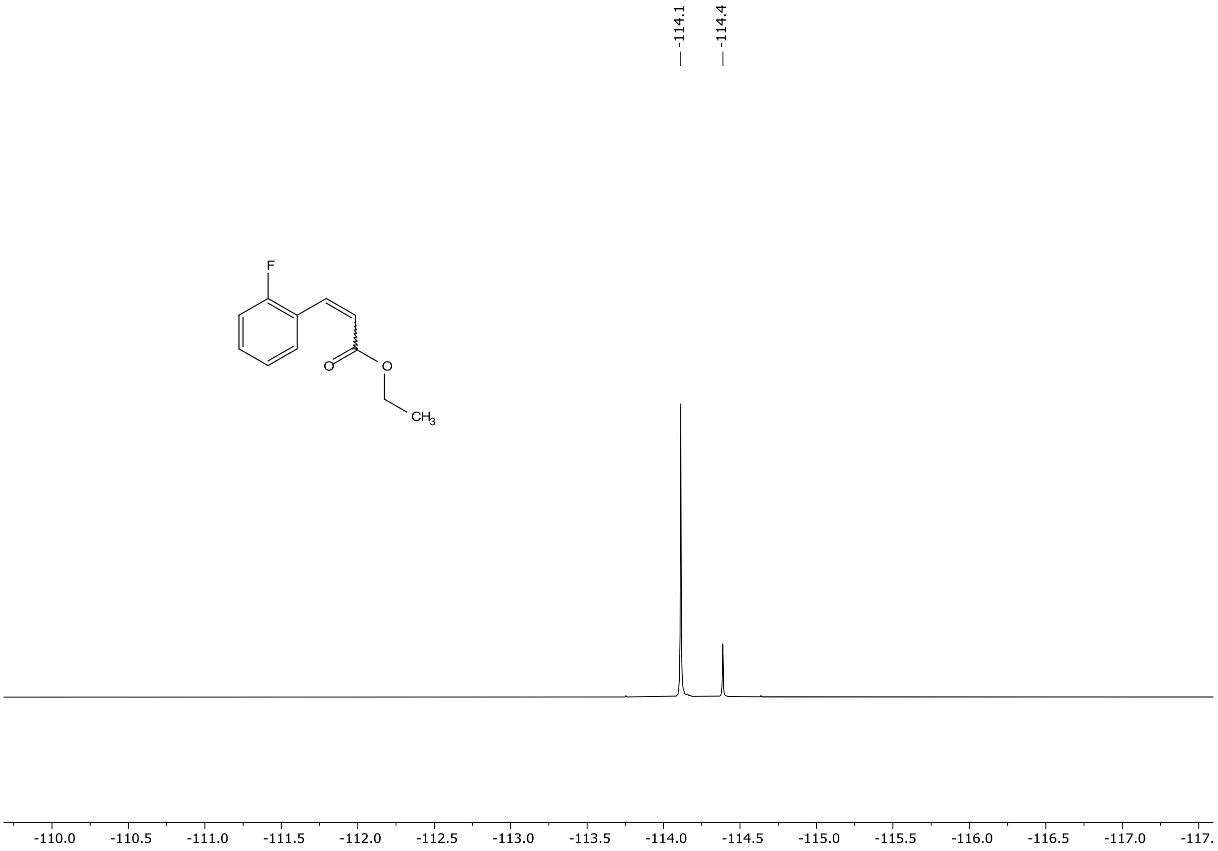
-114.1
-114.4
F
O
O
CH3
-110.0
-110.5
-111.0
-111.5
-112.0
-112.5
-113.0
-113.5
-114.0
-114.5
-115.0
-115.5
-116.0
-116.5
-117.0
-117.

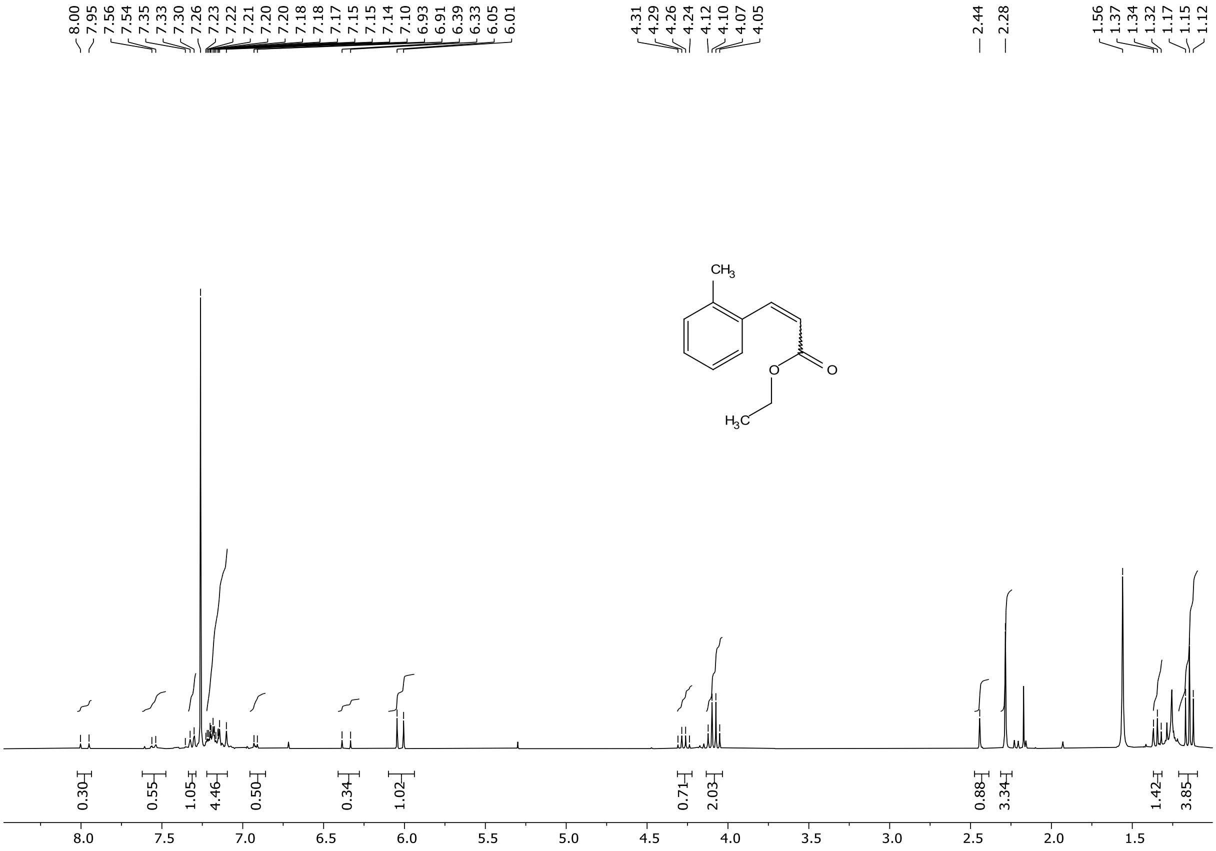
8.00
7.95
7.56
7.54
7.35
7.33
7.30
7.26
7.23
7.22
7.21
7.20
7.20
7.18
7.18
7.17
7.15
7.15
7.14
7.10
6.93
6.91
6.39
6.33
6.05
6.01
4.31
4.29
4.26
4.24
4.12
4.10
4.07
4.05
2.44
2.28
1.56
1.37
1.34
1.32
1.17
1.15
1.12
CH3
O
O
H3C
0.30
0.55
1.05
4.46
0.50
0.34
1.02
0.71
2.03
0.88
3.34
1.42
3.85
8.0
7.5
7.0
6.5
6.0
5.5
5.0
4.5
4.0
3.5
3.0
2.5
2.0
1.5

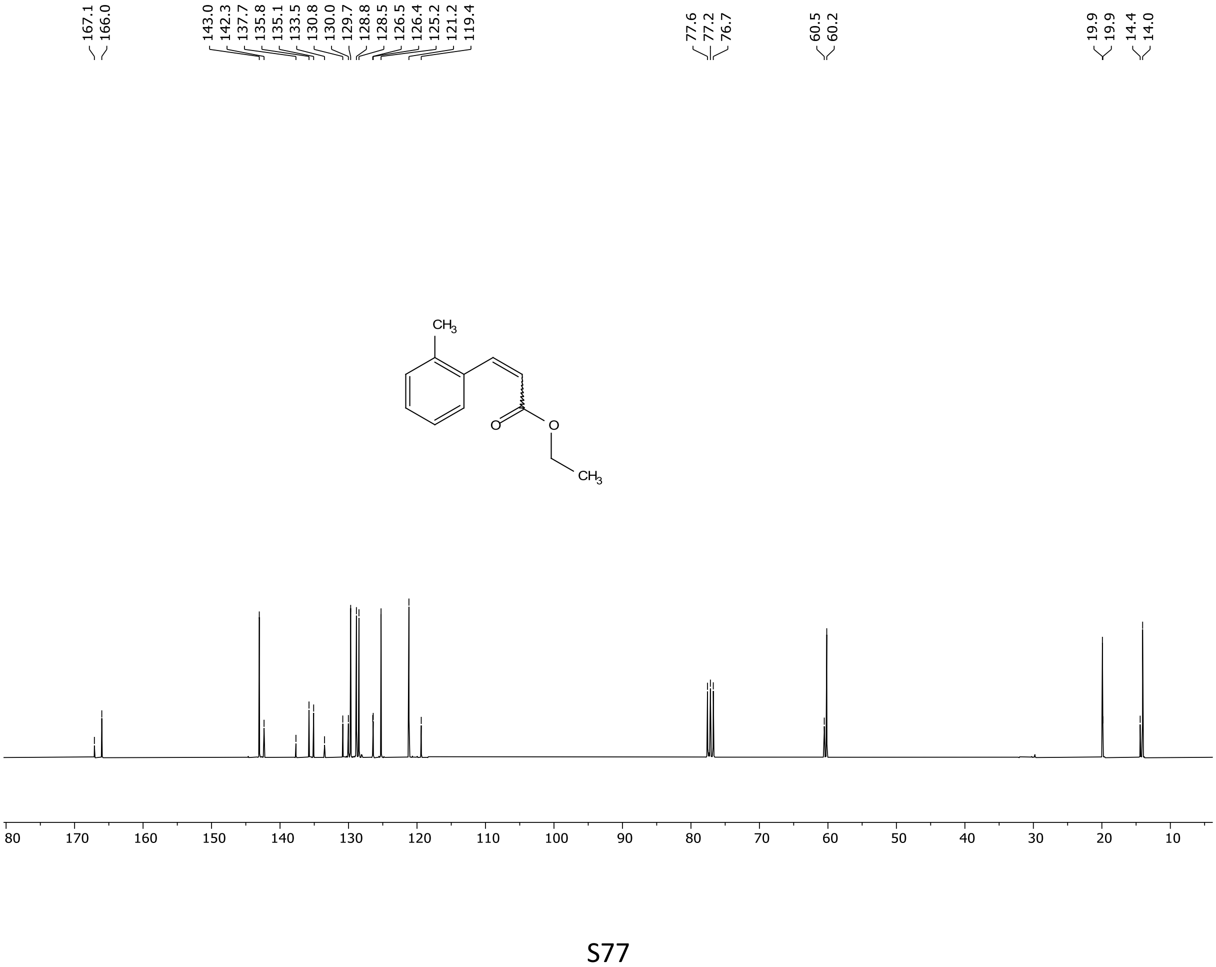
167.1
166.0
143.0
142.3
137.7
135.8
135.1
133.5
130.8
130.0
129.7
128.8
128.5
126.5
126.4
125.2
121.2
119.4
77.6
77.2
76.7
60.5
60.2
19.9
19.9
14.4
14.0
CH3
O
O
CH3
80
170
160
150
140
130
120
110
100
90
80
70
60
50
40
30
20
10

7. References

[1] H. Yang, C. Tian, D. Qiu, H. Tian, G. An, G. Li, *Org. Chem. Front.*, 2019, **6**, 2365–2370.

[2] A. K. Chatterjee, T-LI. Choi, D. P. Sanders, R. H. Grubbs *J. Am. Chem. Soc*. 2003, **125**, 11360–11370

[3] P. Sabila, *Univers. J. Chem*. 2015, **3**, 87-90,

[4] F. Neese, The ORCA Program System. *Wiley Interdiscip.* Rev. Comput. Mol. Sci. 2012, **2**, 73–78. https://doi.org/10.1002/wcms.81.

[5] F. Neese, Software Update: The ORCA Program System, Version 4.0. WIREs Comput. Mol. Sci. 2018, **8**, e1327. https://doi.org/10.1002/wcms.1327.

[6] C. Lee, W. Yang, R. G. Parr, Development of the Colle-Salvetti Correlation-Energy Formula into a Functional of the Electron Density. Phys. Rev. *B* 1988, **37**, 785–789.

[7] A. D. Becke, Density-functional Thermochemistry. III. The Role of Exact Exchange. J. Chem. Phys. 1993, **98,** 5648–5652. https://doi.org/10.1063/1.464913.

[8] D. Andrae, U. Haeussermann, M. Dolg, H. Stoll, H. Preuss, Energy-Adjusted Ab Initio Pseudopotentials for the Second and Third Row Transition Elements. *Theor. Chim. Acta* 1990, **77**, 123–141.

[9] F. Weigend, R. Ahlrichs, Balanced Basis Sets of Split Valence, Triple Zeta Valence and Quadruple Zeta Valence Quality for H to Rn: Design and Assessment of Accuracy. Phys. Chem. Chem. Phys. 2005, **7**, 3297–3305. https://doi.org/10.1039/b508541a.

[10] S. Grimme, J. Antony, S. Ehrlich, H. A. Krieg, Consistent and Accurate Ab Initio Parametrization of Density Functional Dispersion Correction (DFT-D) for the 94 Elements H-Pu. *J. Chem. Phys*. 2010, **132**, 154104. https://doi.org/10.1063/1.3382344.

[11] S. Grimme, S. Ehrlich, L. Goerigk, Effect of the Damping Function in Dispersion Corrected Density Functional Theory. *J. Comput. Chem.* 2011, **32**, 1456–1465. https://doi.org/10.1002/jcc.21759.

[12] F. Weigend, Accurate Coulomb-Fitting Basis Sets for H to Rn. *Phys. Chem. Chem. Phys*. 2006, **8**, 1057–1065. https://doi.org/10.1039/b515623h.

[13] A.-R. Allouche, A. Gabedit Graphical User Interface for Computational Chemistry Softwares. *J. Comput. Chem.* 2011, **32**, 174–182. https://doi.org/10.1002/jcc.21600.
